# Mobility data resolution needed to inform predictive models of spatial epidemic spread from mobile phone data

**DOI:** 10.1371/journal.pcbi.1014427

**Published:** 2026-07-02

**Authors:** Giulia Pullano, Shweta Bansal, Stefania Rubrichi, Vittoria Colizza

**Affiliations:** 1 Department of Biology, Georgetown University, Washington, District of Columbia, United States of America; 2 Orange Research - SENSE, Châtillon, France; 3 Sorbonne Université, INSERM, Sorbonne Université Modeling Outbreaks Center (SUMOC), Institut Pierre Louis d’Epidémiologie et de Santé Publique, Paris, France; Tianjin Medical University, CHINA

## Abstract

Human mobility fundamentally shapes the spatial spread of infectious diseases, yet the level of detail required from mobility data to accurately inform epidemic models remains unclear. Mobile phone records offer unprecedented resolution on population movements, but little attention has been devoted however to determining (i) which aspects of mobility are epidemiologically relevant and (ii) what level of data resolution is necessary to capture spatial invasion dynamics. Using mobile phone records from 9.5 million users in Senegal (approximately 80% of the population), we systematically compare three approaches to aggregating mobility data for epidemic modeling. These approaches span a range of resolutions: high-resolution tracking of all individual displacements between consecutive visited locations (HR), medium-resolution accounting for time spent in all visited locations (MR), and low-resolution identification of the most-visited location (LR). We incorporate these mobility representations into a metapopulation epidemic model that explicitly accounts for transmission from residents, visitors, and returning travelers, and simulate diseases with varying transmissibility corresponding to controlled epidemic conditions, seasonal influenza–like transmission, and highly transmissible pathogens. We find that preserving all observed displacements in individual trajectories does not necessarily improve the epidemiological relevance of mobility in pathogens spatial transmission. Instead, displacement-based networks fragment long-range trips and underestimate key spatial connections relevant for disease spread. In contrast, approaches that capture where individuals spend most of their time (such as home, work, or school) more accurately reproduce spatial invasion patterns. Accounting for additional daily activities beyond these primary locations provides little additional epidemiological information. Our results suggest that lower-resolution mobility indicators capturing time spent at key locations are sufficient to inform predictive epidemic models. These findings have important implications for both epidemic modeling and data governance, indicating that mobile phone data can be aggregated to reduce privacy issues while still providing the essential information needed to model spatial disease transmission.

## Author summary

Human mobility patterns play a crucial role in the spatial spread of infectious diseases, as pathogens move between communities through the movements of infected individuals. During the COVID-19 pandemic, mobile phone companies shared unprecedented amounts of mobility data to support epidemic modeling. However, important questions remain: which aspects of human mobility are truly necessary to capture spatial disease transmission, and how detailed must mobility data be to inform epidemic models? Using mobile phone data from 9.5 million users in Senegal, we systematically compared three ways of summarizing individual mobility patterns for epidemic modeling. These approaches ranged from high-resolution tracking of every individual displacement to simpler representations that identify only the locations where people spend most of their time, such as home, work, or school. We found that tracking every movement does not improve epidemic predictions. Instead, the most informative mobility patterns for modeling spatial disease spread are the ones connecting locations where individuals spend sustained periods of time. Approaches that capture the connections between these key locations reproduce epidemic invasion patterns more accurately than those that record every displacement. These results suggest that highly detailed mobility data may not be necessary for epidemic modeling. Instead, aggregated mobility indicators capturing time spent at major activity locations can provide sufficient information to model spatial transmission. This finding has important implications for both epidemic modeling and data sharing practices, as it indicates that useful mobility data can be shared at lower resolution, reducing potential privacy risks while maintaining scientific value.

## Introduction

Human mobility plays a fundamental role in the spatial spread of infectious diseases [1–3]. While many factors may contribute to epidemic dynamics – including environmental conditions, population susceptibility, pathogen transmissibility, and the built environment – human movement provides the primary mechanism through which pathogens propagate across geographic space. This mechanism explains the spatial invasion patterns observed across diseases such as influenza [4–6], COVID-19 [7–9], and SARS [10–12]. From daily commuting to international travel, the connections people create through movement provide the pathways along which infections spread between communities [13–15], offering novel non-local opportunities for exposure and transmission. Understanding these mobility patterns is therefore essential for anticipating epidemic trajectories and designing effective predictive models and interventions.

For several decades, epidemiological models have relied on traditional sources of mobility information [16], including census records [17], transportation statistics [5], commuting flows [13], and international air travel data [18]. However, these traditional sources often report data at coarse spatial and temporal resolutions and only in specific settings, limiting our ability to detect rapid behavioral shifts during public health emergencies [19].

The proliferation of mobile phones in the last decades has fundamentally transformed the observation of human mobility. By 2025, approximately 5.8 billion people, 71% of the global population, carried mobile devices that continuously generate geolocated data [20], by providing unprecedented insight into human behavior at population scale [16,21,22]. Unlike traditional data sources that capture only specific movement types, mobile phone data comprehensively record all trips, regardless of purpose, distance, or transportation mode, spanning spatial scales from local neighborhood movements to long-distance domestic travel [23]. This detection capability, combined with continuous tracking and high spatial-temporal resolution, has made mobile phones an extraordinarily rich source of mobility information and have opened new possibilities for studying human behavior and modeling disease spread.

Mobile phone data have indeed been widely adopted in spatial epidemic modeling. Theoretical and methodological studies have demonstrated their usefulness for quantifying mobility networks and connectivity patterns [24–27], and they have been applied to a variety of infectious diseases including malaria [25,28], cholera [29,30], schistosomiasis [31], dengue [32], HIV [33,34], 2013–2016 Ebola outbreak [35,36]. The COVID-19 pandemic marked a major turning point in the use of mobility data for public health [37,38]. Facing an unprecedented global emergency, telecommunications companies and technology platforms shared mobility data at unprecedented scales, enabling researchers to track population movements and support public health responses in near real-time [8,39–44].

Despite these advances, two key questions remain unresolved: (i) which characteristics of the mobility process are epidemiologically relevant for spatial disease spread, and (ii) what level of data resolution is actually necessary to capture invasion dynamics? Addressing these questions is essential for developing accurate epidemic models capable of informing public health decision-making. In the post-COVID-19 landscape, access to individual-level trajectories has become increasingly restricted, while aggregated mobility indicators remain the standard approach adopted by telecommunications. Identifying the minimum mobility information required for accurate epidemic prediction would allow data providers to generate indicators that are scientifically meaningful while avoiding the need to share highly detailed individual trajectories.

Integrating mobile phone data into predictive epidemic models requires translating individual-level trajectories into location-level coupling forces, quantitative measures representing the probability of disease transmission between connected places. This process involves two key choices: which locations should be connected (network structure), and how strongly they should be coupled (edge weights). Different aggregation methods encode different assumptions about which aspects of mobility are epidemiologically relevant. Some preserve complete movement sequences, tracking the order of displacements between locations; others focus on time allocation, recording how long individuals spend in different places; and others identify only primary destinations such as home, work, school as the main sites of transmission risk. Despite the widespread adoption of mobile phone data in epidemic modeling, these aggregation choices have not been systematically assessed because mobility data are typically pre-aggregated by data providers and researchers have rarely access to individual trajectories. Determining which mobility features drive disease spread, and at what resolution they must be measured, is therefore both a scientific and an ethical challenge. If highly detailed mobility information provides little improvement over simpler aggregations, unnecessary privacy risks may be introduced without substantial epidemiological benefit. Conversely, overly coarse aggregation may obscure key transmission pathways and reduce predictive accuracy.

Here, we investigate how the level of detail used to describe human mobility influences predictions of spatial epidemic spread. We compare alternative ways of summarizing population movement that capture different degrees of behavioral detail and evaluate how these representations affect predictions of when and where epidemics spread across space. By identifying which aspects of mobility information are essential for capturing spatial disease dynamics, our study clarifies what type of mobility data is most informative for epidemic modeling and highlights the trade-offs between epidemiological accuracy and potential privacy risks.

## Results

To assess how mobility data aggregation shapes epidemic predictions, we constructed three mobility networks from individual mobile phone trajectories of 9.5 million users in Senegal, each encoding a decreasing level of movement detail: a high-resolution network (HR), a medium-resolution network (MR), and a low-resolution network (LR). These networks were integrated into a metapopulation epidemic modeling framework and used to simulate outbreaks across a range of transmission scenarios. We then compared the resulting epidemic arrival times and transmission pathways to evaluate which aspects of mobility are epidemiologically informative.

**Fig 1:**
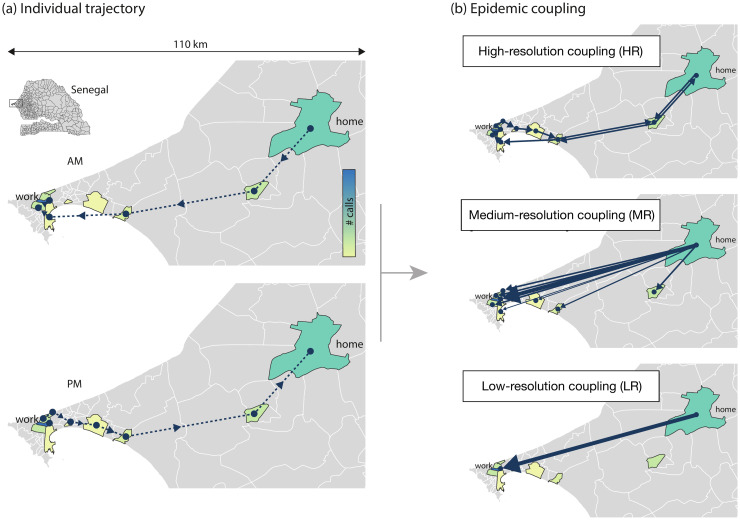
Mobility resolutions. **(a)** Individual trajectory showing mobile phone user movements throughout the day from home to work in the morning (top) and from work to home in the afternoon (bottom), with color indicating number of activities (calls, messages) in each municipality. **(b)** Resulting epidemiological coupling networks for three aggregation methods: HR) High-resolution method linking all consecutive call locations; MR) Medium-resolution method linking home to all visited locations weighted by time spent; LR) Low-resolution method linking home only to the most-visited location. The maps were generated in Python using administrative boundary shapefiles from the Global Administrative Areas database (GADM), available at https://gadm.org.

To evaluate how different levels of mobility data aggregation influence epidemic predictions, we compare three coupling representations derived from mobile phone data (Fig 1): high-resolution tracking of all individual displacements and connecting consecutive visited locations (HR) [45], medium-resolution coupling linking home to all visited locations and weighted by the time individuals spend in these locations (MR) [29], and low-resolution coupling linking home to the most-visited location only (LR) [25]. We first examine the structural differences between the resulting mobility networks, focusing on coupling probabilities, connectivity patterns, and the presence of long-range links. We then quantify how these differences affect simulated epidemic dynamics using a metapopulation model under three transmissibility regimes: low transmission (R₀ = 1.1), representing controlled epidemic conditions such as SARS-CoV-2 under strong interventions; moderate transmission (R₀ = 1.5), consistent with seasonal influenza–like dynamics; and high transmission (R₀ = 3.0), representing highly transmissible pathogens such as early SARS-CoV-2 or Ebola during intense community transmission. Epidemic outcomes are compared in terms of spatial invasion patterns, arrival times across municipalities, and transmission pathways, allowing us to assess how mobility data resolution shapes predicted epidemic spread.

### Location and commuting-based matrices better represent epidemic-relevant mobility

**Fig 2:**
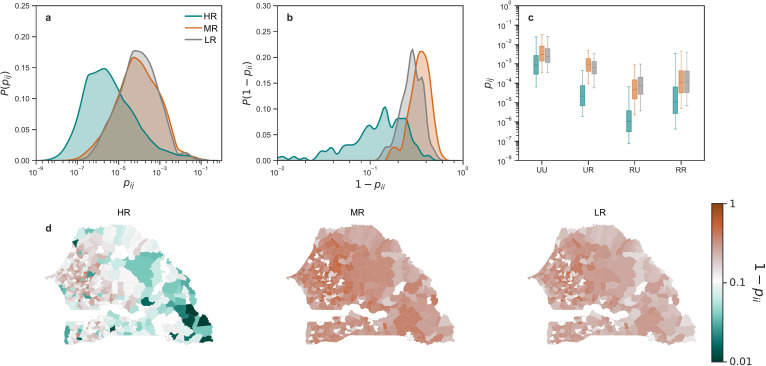
Coupling forces. **a)** Coupling probability distribution in HR, MR, and LR. Distribution includes matrices for all months in 2013. **b)** Outgoing probability (1 - $p_{ii}$) distribution in HR, MR, and LR. Distribution includes matrices for all months in 2013 **c)** Coupling probability stratified by municipality type. Box plots show the 95% reference range across all months for links connecting Urban-Urban (UU), Urban-Rural (UR), Rural-Urban (RU), and Rural-Rural (RR) municipalities. **d)** Spatial distribution of outgoing probability. Maps show the outgoing probability for each municipality in HR, MR, and LR for January 2013. The maps were generated in Python using administrative boundary shapefiles from the Global Administrative Areas database (GADM), available at https://gadm.org.

The three aggregation methods at different spatio-temporal resolutions (HR, MR, LR) yielded 12 daily average directed networks per method, one for each month. The resulting networks exhibit different topologies depending on the aggregation process. Multiple Regression Quadratic Assignment Procedure (MRQAP) revealed high correlations among all methods (regression coefficient $r_{LR,MR}=\text{1}$, $r_{HR,MR}=\text{0}.\text{9}\text{9}$, $r_{HR,LR}=\text{0}.\text{9}\text{9}$). Despite these high correlations, the median coupling probability in HR is approximately one order of magnitude lower than in MR and LR (1.5 × 10 ⁻ ⁴ in LR, 1.0 × 10 ⁻ ⁴ in MR, 8.4 × 10 ⁻ ⁶ in HR, Fig 2a). Largest differences are on links that connect Urban/Rural municipalities (Fig 2c).

Conversely, outgoing probabilities are higher in HR compared with MR and LR (0.86 in HR, 0.62 in MR, 0.69 in LR; Fig 2b). Differences are mainly driven by rural areas in the southwest of Senegal (Fig 2d). Because HR is constructed from consecutive mobile phone events, periods in which individuals remain in the same location generate self-loops, substantially reducing the probability of moving. Trips are partitioned into multiple short consecutive segments, causing the corresponding movement probability to be distributed across several intermediate links rather than concentrated on a single origin–destination connection, as occurs in MR and LR. Because of these two effects, coupling probabilities in HR are smaller and diagonal elements in the HR matrices are larger, whereas non-diagonal elements are smaller, producing the observed differences between HR and the matrices MR and LR.

**Fig 3:**
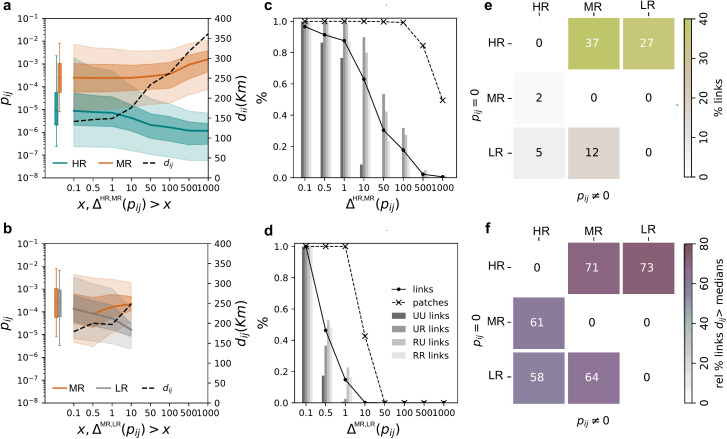
Differences between HR, MR and LR. **a, b)** Coupling probability distributions for common links in all 12 matrices as a function of relative variation threshold **(x)**.Subsets include only links where the relative variation Δ between methods exceeds x. Lines show coupling probabilities for HR and MR (a) or MR and LR **(b)**; dashed lines indicate median geographical distance of links for each subset. **c, d)** Characteristics of link subsets as a function of relative variation threshold (x) for all 12 matrices. Solid line: percentage of links in each subset. Dotted line: percentage of locations (nodes) represented in each subset. Stacked bars: distribution of links by municipality type (UU: Urban-Urban, UR: Urban-Rural, RU: Rural-Urban, RR: Rural-Rural). **e-f)** Matrices showing unique links unique to each method for all 12 matrices. Yellow matrix: percentage of links present in the column method but absent in the row method. Violet matrix: among unique links, percentage connecting municipalities at distances greater than the median.

We then examined differences between coupling matrices by analyzing (i) links shared across aggregation methods and (ii) links present in one aggregation but absent in the others (Fig 3). For links shared between HR and MR, coupling probabilities in HR are systematically lower, with differences increasing with geographical distance (Fig 3a–3c). When considering links with relative variation above a threshold ($\Delta^{HR,MR}\left(p_{ij}\right)>\text{x}$), the most discrepant links show coupling probabilities in HR up to 1,000 times lower than in MR (Fig 3a). These strongly discrepant links predominantly connect rural and urban municipalities at long distances (Fig 3a–3c). The comparison between HR and MR revealed similar patterns. In contrast, links shared between MR and LR show much smaller differences. Coupling probabilities in LR differ by at most one order of magnitude from MR, and these differences remain relatively stable across geographical distances (Fig 3b–3d). Also in this case, the largest differences involve links connecting rural areas (Fig 3d). We then examined links present in only one aggregation. 37% of links in MR are absent from HR (Fig 3e, yellow matrix) and 71% of these unique MR links connect municipalities separated by distances greater than the median (Fig 3f, violet matrix). Similarly, 27% of links in LR are absent from HR, with 73% corresponding to long-range connections. Conversely, only 12% of links in MR are absent from LR (Fig 3e). These MR-only links represent connections to secondary activity locations with lower mobility flows. This small difference indicates that the additional destinations captured by MR, beyond the primary workplace/school in LR, contribute little to the overall mobility network structure.

Together, these findings demonstrate that by tracking every displacement, HR fragments trips and misses the long-distance connections critical for spatial disease invasion. In contrast, methods that aggregate mobility based on where individuals spend time (MR and LR) naturally capture the long-range locations where sustained exposure may occur.

### Displacement-based coupling both slows and constrains simulated epidemic spread

**Fig 4:**
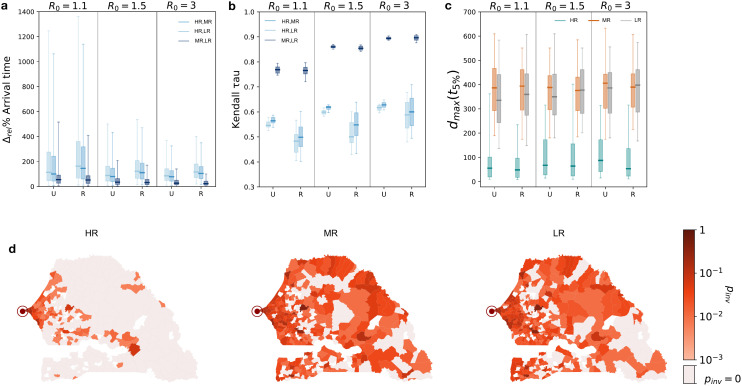
Differences on simulated epidemic outcomes. **a)** Relative variation in arrival times for each municipality between combinations of the three methods. **b)** Kendall tau rank correlation coefficients comparing arrival time orderings between HR vs. MR, HR vs. LR, and MR vs. LR. **c)** Maximum geographical distance reached from the epidemic seed at $t_{\text{5}\%}.$ $t_{\text{5}\%}$ is defined as the time when the 5% of the municipalities have become infected ${(t}_{\text{5}\%})$.Boxplots in panels (a–c) indicate the 95% reference range. **d)** Spatial distribution in map of the invasion ${(p}_{inv})$ at $t_{\text{5}\%\;}$ for the three methods HR, MR, LR, for $R_{\text{0}}=\text{1}.\text{1},\;$ with a municipality of the capital Dakar as seed. The location with the red dot is the epidemic seed. The maps were generated in Python using administrative boundary shapefiles from the Global Administrative Areas database (GADM), available at https://gadm.org.

The lower coupling probabilities observed in HR translated directly into delayed epidemic arrival times. In HR, infections reached municipalities between a few weeks and 410 days after seeding, whereas in MR and LR, arrival times did not exceed 250 days (Fig 4a). The median relative variation in arrival times between MR and LR was near 40 across all R₀ values (Fig 4a), whereas comparisons between HR and either MR or LR showed median variations around 100%.

MR and LR not only produced similar arrival time distributions but also predicted nearly identical spatial invasion sequences, with high rank correlation (Kendall τ = 0.75-0.90; Fig 4b). In contrast, HR showed substantially lower rank correlation with both MR and LR, indicating fundamentally different invasion pathways. The spatial extent of early spread also differed markedly across methods. At t₅% - the time when 5% of the municipalities have been reached by the transmission - the maximum geographical distance reached from the seed was substantially smaller in HR than in MR and LR (Fig 4c), reflecting HR systematic underestimation of long-range transmission. With R₀ = 1.1, the invasion probability of the top 5% infected municipalities in HR clustered tightly around the seed (e.g., near Dakar), whereas MR and LR produced more spatially heterogeneous invasion patterns with early spread to distant locations (Fig 4e).

**Fig 5:**
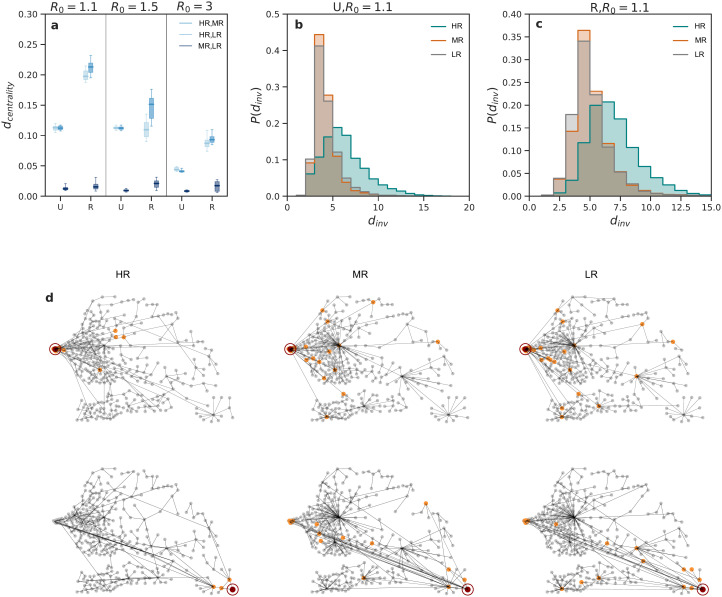
Epidemic invasion trees. Epidemic invasion trees were computed for $R_{\text{0}}=\text{1}.\text{1},\text{1}.\text{5},\text{3}$ considering both an urban (U) and a rural seed (R). **a)** Boxplots show the 95% reference range of the betweenness centrality distance index between invasion trees from each pair of methods. **b-c)** Distribution of the invasion distances across the three methods for $R_{\text{0}}=\text{1}.\text{1}$. **d)** The invasion trees for $R_{\text{0}}=\text{1}.\text{1}$. Epidemic seeds are shown in red. Locations directly infected by the seed are in orange, and locations infected by secondary spread are in gray. The maps were generated in Python using administrative boundary shapefiles from the Global Administrative Areas database (GADM), available at https://gadm.org.

Beyond arrival times, the spatial structure of epidemic invasion differed across aggregation methods. We analyzed invasion trees, representing the most likely transmission pathways through which the epidemic spreads from seed locations, for two contrasting scenarios: seeding from Dakar (the capital of Senegal) and from a rural municipality in Saraya (the rural and most distant location from Dakar, in the top 2% for variation in outgoing probability between HR and MR). MR and LR produced strikingly similar invasion trees, with betweenness centrality distances below 0.05, indicating nearly identical hierarchical spread patterns (Fig 5a). In contrast, HR differed substantially from both MR and LR (betweenness distances 0.05-0.25), revealing fundamentally different transmission pathways. In HR, the distribution exhibits a long tail extending to $d_{inv}$ = 15–20, indicating that some municipalities are reached only through many sequential transmission steps from one location to another (Fig 5b–5c). In contrast, MR and LR show more compact distributions with maximum invasion distances of approximately 10 steps, reflecting spatial spread through long-range mobility connections.

**Fig 6:**
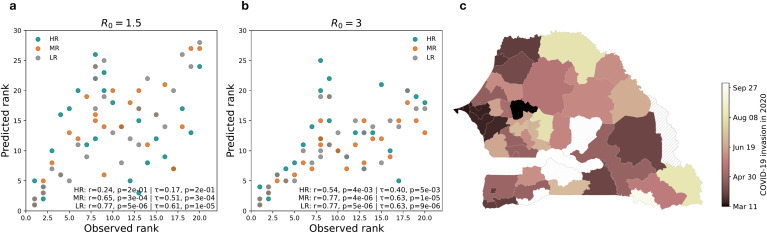
Comparison of observed and predicted COVID-19 invasion during the 2020 first wave. **(a)** Scatter plot comparing the ranking of observed early COVID-19 arrival times in 2020 with model-predicted arrival times across departments, for the HR, MR, and LR methods. Model predictions correspond to the scenario with R0 = 1.5 and Dakar as the seed location. **(b)** Same as panel **(a)**, but for the scenario with R0 = 3 and Dakar as the seed location. Spearman’s (r) and Kendall’s (τ) correlation coefficients, together with their associated *p*-values, are reported in both panels. **(c)** Spatial distribution of the COVID-19 invasion in Senegal in 2020, shown as department-level arrival times. Dakar and Touba colored in black are the first ones to report confirmed COVID-19 cases on March, 11, 2020. The maps were generated in Python using administrative boundary shapefiles from the Global Administrative Areas database (GADM), available at https://gadm.org.

To empirically ground our theoretical findings, we analyzed the invasion dynamics of the early COVID-19 pandemic in Senegal in 2020. The epidemic exhibited rapid long-range jumps between major urban hubs rather than gradual geographic diffusion (Fig 6c). The first confirmed cases were reported in Dakar, the capital and location of the international airport where the virus was first introduced. The virus was also detected in Touba, a major religious and economic hub located several hundred kilometers from Dakar. The rapid spread across distant locations highlights the importance of long-range mobility connections in shaping the early spatial spread of the epidemic. We compared the observed invasion order across departments with the arrival times predicted by the three mobility representations. Because early estimates of COVID-19 transmissibility varied widely (approximately R₀ = 1.5–3.5), we evaluated model predictions across this plausible range. The heterogeneous invasion pattern observed in Senegal is well reproduced by the MR and LR models but not by the HR model. For R₀ = 1.5, the predicted and observed arrival rankings are strongly correlated for MR and LR (Spearman rank correlations of r = 0.65 and 0.77, respectively), whereas HR shows weak and non-significant agreement (r = 0.24; Fig 6a). Consistent results are obtained using Kendall’s τ (τ = 0.51 and 0.61 for MR and LR respectively, versus non-significant association for HR). At the higher end of the estimated range (R₀ = 3), MR and LR continued to reproduce the observed invasion ordering (Spearman’s r = 0.77 for both models), while HR showed weaker agreement (r = 0.54; Fig 6b). The consistent performance of MR and LR across this empirically plausible range of R₀ values indicates that mobility representations capturing where individuals spend time better reproduce the spatial ordering of epidemic invasion. These findings demonstrate that intermediate-range and long-range mobility coupling, rather than purely local displacement transmission, were the dominant drivers of the heterogeneous spatial spread observed during the early COVID-19 epidemic in Senegal.

Finally, to test the robustness of our findings, we performed a sensitivity analysis by introducing time-corrected versions of the three coupling matrices: HR’, MR’, and LR’. Those matrices are designed to reduce the bias introduced by heterogeneity in users’ calling activity. HR’ redefines displacement weight as the time elapsed between consecutive moves; MR’ replaces call counts with the effective time spent in each visited location; and LR’ applies the same correction to the most visited location matrix, ensuring coupling strengths reflect time allocation rather than call frequency (see S1 Text for details).

While MR’ and LR’ showed minimal differences from their original counterparts in terms of epidemic outcomes, HR ‘demonstrated substantial improvements over HR, producing spatial diffusion patterns more similar to MR and LR (Fig O in S1 Text – Fig S in S1 Text). However, HR’ still exhibited limitations, with invasion distance distributions remaining higher than in MR and LR due to approximately 40% of epidemiologically relevant links present in MR and LR being absent in HR’ (Fig O in S1 Text). This suggests that even refined displacement-based approaches may not adequately capture epidemiological couplings between locations for diseases where daily mobility patterns drive transmission.

## Discussion

We systematically evaluated how different levels of mobile phone data aggregation affects predictions of spatial epidemic spread. Using mobile phone data from Senegal, we compared three approaches to constructing coupling matrices from individual trajectories: high-resolution tracking of all displacements (HR) [45], medium-resolution representations based on time spent across visited locations (MR) [29], and low-resolution identification of the most-visited location (LR) [25]. Our results show that increasing mobility resolution does not necessarily improve epidemic predictions. Instead, approaches that capture where individuals spend most of their time produce more realistic spatial invasion patterns than those that track every displacement.

Two main findings emerge from our analysis. First, preserving maximum mobility detail by tracking all displacements (HR), without accounting for time spent in each place, produces biased epidemic predictions, including delayed arrival times, underestimated long-range transmission, and unrealistic radial diffusion patterns, compared with the other approaches (MR, LR) and previous works [27,46]. HR yields coupling probabilities approximately one order of magnitude lower than MR and LR and misses a substantial fraction of long-range connections. These results align with previous work showing that the duration of travel and time spent at destination are key drivers of transmission risk [47,48]. Second, MR and LR produce highly similar and more realistic epidemic patterns, indicating that identifying the locations where individuals spend sustained periods of time—such as homes, workplaces, and schools—is sufficient to capture the mobility structure relevant for spatial disease transmission. Tracking secondary activities beyond these most-visited location provides minimal additional epidemiological value for spatial disease spread. This is consistent with a substantial body of literature demonstrating that commuting fluxes between home and regularly visited locations are primary drivers of spatial epidemic spread, shaping geographic invasion [13,14].

The poor performance of HR stems from a mismatch between mobility representation and epidemiological processes driving spatial transmission. By linking consecutive mobile phone events, HR fragments continuous trips into multiple short segments and fails to identify the true origin–destination structure of mobility, connecting locations where sustained transmission occurs. This fragmentation has two critical consequences: (i) it substantially underestimates long-distance connectivity by breaking journeys into short hops, (ii) it assigns equal weight to each displacement regardless of time spent, failing to distinguish epidemiologically important locations (homes, workplaces, schools) from places individuals merely pass through.

In contrast, MR and LR focus on time allocation, which better reflects exposure opportunities. Time spent in a location is strongly related to exposure opportunity and transmission risk [48], whereas brief passages through intermediate locations contribute little to disease spread. By preserving the directional relationship between residences and destinations, MR and LR also reconstruct the characteristic spatial pathways through which epidemics propagate between communities [10]. Consistent with our simulation results, comparison with the observed heterogenous spatial invasion of COVID-19 in Senegal [49] shows that these representations better reproduce the ordering of epidemic arrival across locations.

Our findings are likely to generalize beyond the specific context analyzed here. Across the range of transmissibility explored (R₀ = 1.1, 1.5, 3.0), representing controlled, moderate, and high transmission scenarios, the relative performance of the mobility representations remained consistent. Differences between methods were most pronounced at low transmissibility (R₀ = 1.1), when spatial heterogeneity plays a greater role in shaping epidemic spread, and diminished at higher transmission levels. This robustness suggests that our conclusions reflect fundamental interactions between mobility structure and transmission processes rather than pathogen-specific characteristics. Although mobility patterns differ across socioeconomic settings, large-scale human movement is consistently characterized by structural regularities that can be captured by gravity- or radiation-type models across both high-income and low- and middle-income countries [14,16]. These regularities reflect common constraints on travel behavior, such as population size, distance, and activity patterns. While the magnitude of mobility flows and the relative importance of commuting versus long-distance travel may vary across contexts, the underlying origin–destination structure of human mobility tends to remain similar. Spatial scale may also influence the magnitude of differences between aggregation methods: at coarser spatial resolutions, averaging across locations may reduce the apparent discrepancies between mobility representations [27]. However, the central finding of our study remains robust: epidemiologically meaningful connectivity is better captured by linking residences to destinations where individuals spend time than by reconstructing sequences of consecutive displacements.

While this study focuses on Senegal, we are aware that mobility patterns differ across socioeconomic contexts, with implications for how aggregation methods may behave. In low-income settings, daily mobility often consists of fewer, shorter trips (mean ≈5 km), whereas in high-income settings mobility typically exhibits more frequent and longer commuting trips (mean ≈10–15 km). That said, a substantial body of literature has established that human mobility is well described by parsimonious generative models, gravity and radiation models, across both high-income and low- and middle-income country (LMIC) settings [16], and Balcan et al. [14] demonstrated that commuting flows can be reliably fitted at a global scale, suggesting that the fundamental structure of daily human circulation between home and regularly visited locations is a general feature of mobility that transcends development contexts. These structural differences, moreover, do not alter our methodological conclusions, because the failure of HR arises from trip fragmentation rather than from any property of trip length itself: by chaining sequentially visited locations, HR severs the epidemiologically meaningful connection between where people reside and where they spend significant time, misrepresenting the coupling forces that drive epidemic invasion regardless of characteristic trip distances. While the specific magnitude of differences between methods may vary with local mobility patterns, infrastructure, and socioeconomic conditions, the core distinction between origin-destination preservation and trip fragmentation should generalize across settings. The spatial scale of analysis may nonetheless affect the generalizability of our quantitative results, as using municipalities as spatial units allows us to capture substantial heterogeneity across locations, whereas coarser aggregations would smooth over these differences and likely attenuate the observed magnitude of discrepancies between methods. Indeed, we expect differences between methods to diminish at coarser scales, where averaging obscures the heterogeneities that distinguish approaches, but empirical evaluation across scales remains an important direction for future work. Validation in diverse geographic and economic contexts would further strengthen the universality of these findings.

Our study has a number of limitations. First, although we compared simulated invasion patterns with observed COVID-19 spatial spread in Senegal, we did not perform a full calibration of the epidemic model to incidence time series or evaluate predictive accuracy across the entire epidemic wave. Our analysis instead focuses on the comparative impact of mobility representations on spatial transmission patterns. Second, mobile phone data capture user activity only when calls or messages occur, which may introduce biases in the reconstruction of mobility trajectories. Although our aggregation methods partially mitigate this issue by focusing on time allocation rather than individual events, variations in phone usage behavior may still influence estimated mobility patterns. The landscape of mobility data has evolved considerably, and a broader range of data sources, including XDR records, GPS traces, WI-FI access logs, and other high-resolution digital traces, now offer complementary approaches that may reduce reliance on event-driven CDR data and further improve the accuracy of mobility estimation. Third, our analysis focuses on mobility-driven transmission and does not explicitly incorporate other factors that may influence spatial spread, such as heterogeneities in contact behavior, local interventions, or environmental conditions. Despite these limitations, our results provide practical guidance for both epidemic modeling and mobility data sharing.

In the post-COVID-19 era, direct access to individual trajectories has become increasingly restricted, while aggregated mobility indicators are more widely available. Our findings challenge the assumption that more granular data yields better epidemic predictions. Instead, aggregated indicators describing time spent at major activity locations appear sufficient for predictive spatial models. This insight supports the development of privacy-preserving data-sharing frameworks that reduce the sensitivity of mobility data while preserving their epidemiological value.

## Methods

### Mobile phone dataset

We analyzed anonymized Call Detail Records (CDRs) provided by Orange Senegal covering January–December 2013. Each CDR corresponds to a call or text and includes a timestamp and the serving cell tower, allowing the user’s location to be inferred at the antenna level. The dataset covers approximately 9.57 million users (≈80% of the national population), mapped to municipalities via 15,999 antennas distributed across the territory. To ensure spatial consistency, we retained only municipalities covered throughout 2013, resulting in 394 municipalities (46 urban and 348 rural). The 46 urban municipalities are located in the following cities: Dakar, Guediawaye, Pikine, Rufisque, Thiès. We kept users active for more than 30 days and excluded extreme activity profiles (>1000 events/week) to reduce biases associated with bots and call centers.

### From individual trajectories to coupling matrices

Coupling matrices quantify the connectivity strength between spatial locations that enables pathogen transmission through human movement. Each element *p*_*ij*_ represents the monthly averaged probability that an individual residing in i travels to location *j* in a given day. Consequently*, p*_*ij*_ captures the opportunity for exposure of individuals residing in location *i* to individuals present in location *j* and potential transmission in *j*. Mobile phone communication events (calls and SMS) provide geo-location data at cell tower resolution. Each event marks the user’s physical presence at a specific time and place, allowing us to track individual daily consecutive displacements and time spent in different locations as a proxy of the number of activities in a location. By aggregating individual mobility patterns across all users, we extracted three coupling matrices at different resolution levels, computing 12 daily average matrices for each month in 2013 at the municipality level for each method. The coupling matrices of the three methods are defined as follows:

High resolution coupling matrix (HR) [45]


$$\begin{array}{c} \overset{HR}{\underset{ij}{p}}=\frac{\sum_{u_{i}}\overset{u}{\underset{i,j}{HR}}}{\sum_{k}\sum_{u_{i}}\overset{u}{\underset{i,j}{HR}}} \end{array}$$

where $\overset{u}{\underset{i,j}{HR}}\;$ is the number of times user *u* moves from location *i* to *j* on consecutive calls. This high-resolution method keeps the temporal sequence of individual displacements. The coupling represents the probability of moving from one place to another, without considering the time spent in each location. Each displacement has the same weight regardless of the time elapsed between consecutive calls.

Medium resolution coupling matrix (MR) [29]


$$\begin{array}{c} \overset{MR}{\underset{ij}{p}}=\frac{\sum_{u_{i}}\overset{u_{i}}{\underset{i,j}{MR}}}{\sum_{k}\sum_{u_{i}}\overset{u_{i}}{\underset{i,k}{MR}}} \end{array}$$

where $\overset{u_{i}}{\underset{i,j}{MR}}\;$ is the number of calls made in *j* by user $u_{i}\;$ living in *i*. Home locations are identified as the location where users made the most calls during nighttime (7 pm- 7 am), a well-established method used in literature [7]. This medium-resolution method loses information about the temporal sequence of displacements but captures the coupling between home and all visited locations. Under the assumption that the call frequency is proportional to the time spent in a location, this method measures probability of being in j given residence in *i.*

Low resolution coupling matrix (LR) [25]


$$\begin{array}{c} \overset{LR}{\underset{ij}{p}}=\frac{\sum_{u_{i}}\overset{u_{i}}{\underset{i,j}{LR}}}{\sum_{k}\sum_{u_{i}}\overset{u_{i}}{\underset{i,k}{LR}}} \end{array}$$

$\text{where }\overset{u_{i}}{\underset{i,j}{LR}}$ is the amount of time user $u_{i}$ spends at his most visited location daily in *j*. The most visited location was defined as where a user made the maximum number of calls during a 24-hour time period. This lowest-resolution method considers only locations where users spend most of their time (home, work or school), neglecting all other less-visited locations. The coupling probability represents the probability of spending most of the time in *j* given the residence in *i.* Since the most visited location often coincides with the workplace, this method approximates commuting fluxes [9].

We also tested refined versions of these matrices to correct for biases in user activity patterns and time representation (see S1 Text for details).

### Epidemic metapopulation model

To quantify the impact of the coupling matrices on the modelled epidemic diffusion, we developed a stochastic, discrete-time and non-Markovian metapopulation model [4,5]. The model accounts for disease transmission arising from spatial co-location of individuals due to daily mobility patterns. The Senegalese population was spatially divided in 394 municipalities, with links between municipalities defined by the time-varying coupling matrices ${\text{p}}_{\text{ij}}$.

The force of infection in location *i* at time *t* is given by:


$$\begin{array}{c} {\;\lambda }_{\text{i}}=\lambda_{\text{ii}}+\sum_{\text{i}\neq \text{j}}\overset{\text{v}}{\underset{\text{ji}}{\lambda }}+\sum_{\text{i}\neq \text{j}}\overset{\text{r}}{\underset{\text{ij}}{\lambda }} \end{array}$$

with


$$\begin{array}{c} {\;\lambda }_{\text{ii}}=\beta \overset{\text{2}}{\underset{\text{ii}}{\text{p}}}\frac{{\text{I}}_{\text{i}}}{\hat{{\text{N}}_{\text{i}}}};\overset{\text{v}}{\underset{\text{ji}}{\;\lambda }}=\beta \;{\text{p}}_{\text{ii}}{\text{p}}_{\text{ji}}\frac{{\text{I}}_{\text{j}}}{\hat{{\text{N}}_{\text{i}}}};\;\overset{\text{r}}{\underset{\text{ij}}{\lambda }}=\beta \;{\text{p}}_{\text{ij}}\frac{\hat{{\text{I}}_{\text{j}}}}{\hat{{\text{N}}_{\text{j}}}} \end{array}$$

$p_{ij}$ is the coupling probability between location *i* and *j* at time *t* and β the transmissibility rate. The time index *t* is omi*tt*ed from the formulas for clarity.

The three terms of the force of infections correspond to three distinct transmission contributions: (${\;\lambda }_{\text{ii}}$) residents who remain in their home municipality and get infected locally; ($\overset{\text{v}}{\underset{\text{ji}}{\;\lambda }}$) infected visitors from other municipalities temporarily present in *i*; and ($\overset{\text{r}}{\underset{\text{ij}}{\lambda }}$) residents of *i* who are visiting location j for the day, get infected in j and return to *i* [50]. All three pathways are implicitly accounted for through the coupling probabilities ${\text{p}}_{\text{ij}}$, consistently with the first-order equilibrium solution derived analytically in Balcan et al. [14].

The effective population at time *t* is:


$$\begin{array}{c} \hat{N_{i}\;}=p_{ii}N_{i}+\sum_{j}p_{ji}N_{j} \end{array}$$

and the effective number of infections in *i* at time *t* is:


$$\begin{array}{c} \hat{I_{i}}=p_{ii}I_{i}+\sum_{j}p_{ji}I_{j\;} \end{array}$$

$\hat{N_{i}}\;and\;\hat{I_{i}}$ are time-dependent quantities recomputed at each discrete time step to reflect the instantaneous mixing configuration of the population across locations.

We explored three epidemic scenarios corresponding to different transmission intensities: (i) low transmissibility (R0 = 1.1), representing controlled epidemic conditions, as observed for SARS-CoV-2 during periods of effective non-pharmaceutical interventions; (ii) medium transmissibility (R0 = 1.5), representing moderate transmissibility, consistent with typical estimates reported for seasonal influenza and the 2009 H1N1 pandemic; (iii) high transmissibility (R0 = 3), compatible with estimates reported for SARS-CoV-2 in susceptible populations and for Ebola virus disease during intense community transmission.

### Comparison between coupling matrices

We compared the three coupling matrices through network analysis and epidemic outcomes. For the network analysis, we performed Multiple Regression Quadratic Assignment Procedure (MRQAP) to test correlation between coupling probabilities [51]. This method is specifically designed for network data where observations are not independent. We examined relative variations in coupling probabilities and outgoing probabilities, stratifying by urban/rural municipality pairs and geographical distance (computed using the Haversine formula). The outgoing probability from location *i* is defined as follows:


$$\begin{array}{c} p_{out}=\sum_{i\neq j}p_{ij}=\text{1}-p_{ii} \end{array}$$

$\text{where }p_{ii}$ is the probability of not moving.

### Comparison between simulated epidemic outcomes

For epidemic outcomes, we tracked two main observables (1) the arrival time t_a_ of the epidemic in each location, defined as the first time that 10 individuals become infected; (2) the epidemic invasion tree [14] which represents the most likely transmission path. For the invasion tree, whenever a disease-free location *i* experienced an infection, we tracked a directed link from the location of origin. For each scenario, for each run, we extracted the invasion path. By cumulating over all runs, we obtained an invasion path where link weights represent the percentage of runs in which that link appeared. The invasion tree was extracted as the maximum spanning tree of this weighted invasion path. To avoid the stochastic fluctuations of the epidemic simulations, we computed invasion trees by doing 1000 runs, and selecting randomly for 50 times 400 runs.

We compared the invasion trees using (a) the betweenness centrality distance [52]; and (b) the invasion distance measured as the number of edges connecting each node with the seed location of the invasion tree.

## Supporting information

S1 Text Supplementary material. Extended methods and analyses.
(DOCX)
