# Supplementary material for: Mobility data resolution needed to inform predictive models of spatial epidemic spread from mobile phone data
Source: PLoS Comput Biol. 2026 Jul 2;22(7):e1014427. doi: 10.1371/journal.pcbi.1014427 (PMC13327251; doi:10.1371/journal.pcbi.1014427)
Supplement: S1 Text — Extended methods and analyses. (DOCX) [file pcbi.1014427.s001.docx]

**S1 Text - Supplementary Material**

**Mobility data resolution needed to inform predictive models of spatial epidemic spread from mobile phone data**

Giulia Pullano^1^, Shweta Bansal^1^, Stefania Rubrichi^2^, Vittoria Colizza^3^

*^1^* Department of Biology, Georgetown University, Washington, District of Columbia, United States of America

*^2^ Orange Research - SENSE, Châtillon, France*

*^3^ Sorbonne Université, INSERM, Institut Pierre Louis d’Epidémiologie et de Santé Publique, Paris, France*

^*^Corresponding author Vittoria Colizza, email: vittoria.colizza@inserm.fr

Table of Contents

[Mobile phone dataset 2](#_Toc220510167)

[Statistical comparison on coupling matrices 2](#_Toc220510168)

[The metapopulation model 5](#_Toc220510169)

[Epidemic simulations 6](#_Toc220510170)

[Complementary analysis 6](#_Toc220510171)

[Sensitivity analysis on HR, MR, LR 10](#_Toc220510172)

[Corrected high-resolution matrix (HR’) 10](#_Toc220510173)

[Normalized high-resolution matrix ($HRnorm$) 11](#_Toc220510174)

[Corrected medium-resolution matrix ($MR')$ 11](#_Toc220510175)

[Corrected low-resolution matrix ($LR')$ 12](#_Toc220510176)

[Sensitivity analysis results (HR’, MR’, LR’) 12](#_Toc220510177)

[Sensitivity analysis results ($HRnorm$) 15](#_Toc220510178)

[References 16](#_Toc220510179)

# **Mobile phone dataset**

The dataset consists of 15,859,942,126 CDRs from Orange Senegal, including both voice calls and SMS exchanges generated whenever a subscriber performs an activity. Each record contains the caller and callee identifiers, timestamp, duration, communication type (national/international, incoming/outgoing), and the identifier of the antenna handling the event. Antennas are heterogeneously distributed across Senegal, covering all 46 urban municipalities and 357 out of 437 rural ones, but active coverage varies over time. Due to temporal fluctuations in active antennas and resulting coverage gaps, only municipalities continuously covered for the entire 2013 period were retained (n=394). User filtering required >30 active days and <1000 events per week; the latter criterion limits artifacts from bots and call centers.

**Statistical comparison on coupling matrices**

**Fig** **A in S1 text** shows that the largest differences occur between HR and MR, and between HR and LR. The relative variation in outgoing probabilities between HR and MR or LR ranges from 1 to 10 (excluding a few outliers), while the relative variation between MR and LR does not exceed 1.


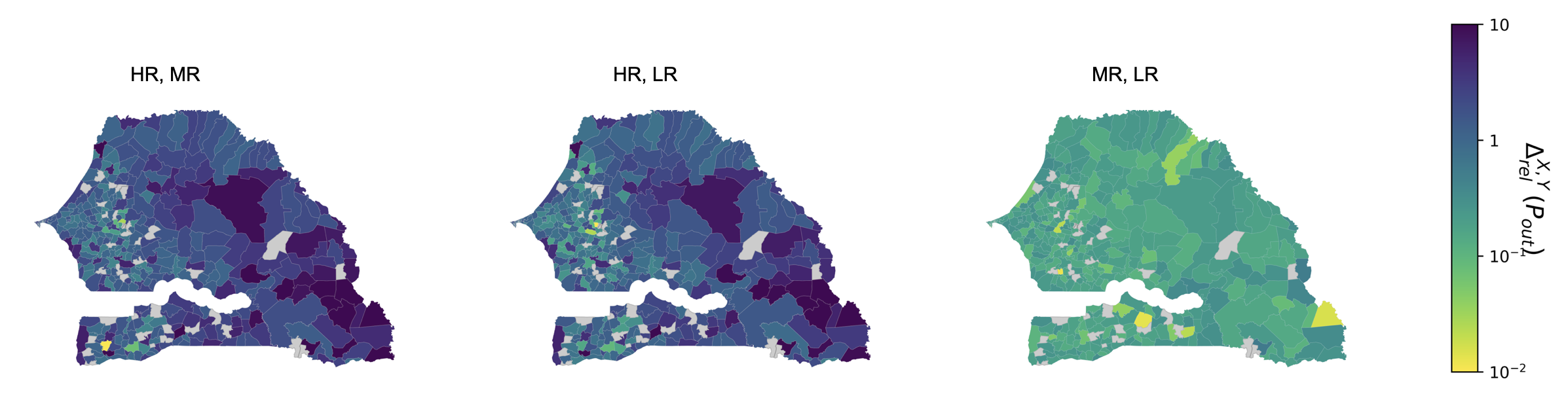


**Fig A in S1 text.** Relative variation of the outgoing probability between HR vs MR, HR vs LR and LR vs MR. The maps were generated in Python using administrative boundary shapefiles from the Global Administrative Areas database (GADM), available at <https://gadm.org>.

We investigated the relationship between coupling probabilities and the geographical distance of connected links. As expected, **Fig B in S1 text** (left) shows that coupling probability decreases with increasing geographical distance in all methods. **Fig B in S1 text** (right) shows that in HR, approximately 70% of links connect municipalities separated by less than 200 km, while in MR and LR, this proportion is around 50%.


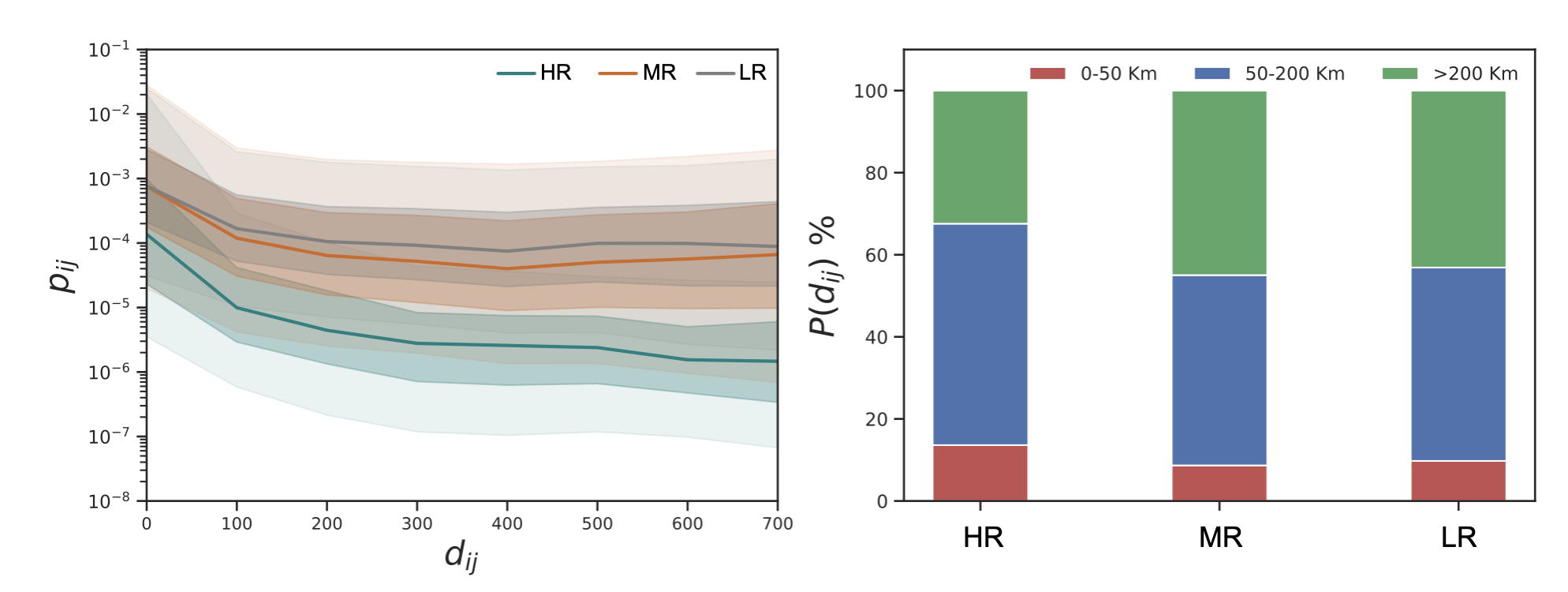


**Fig B in S1 Text**. left) Relation between the coupling probability and the geographical distance of the connected links in HR, MR, LR. right) Percentage of the connected municipalities in HR, MR, LR in a given geographical distance range.

To study the correlations of coupling patterns among the 12-month time periods of the matrices, we implemented a hierarchical clustering. For each month, we constructed the vector of all pairwise coupling probability among the locations. Then, we implemented the Pearson correlations $\rho$ among any two vectors of the 12 monthly periods, and we define a dissimilarity matrix 12 x 12 in which each element $m_{ij}=1-\rho_{ij}.$ The hierarchical clustering is evaluated on the Euclidean distance between any two elements of the matrix.

**Fig C in S1 Text** demonstrates that MR and LR reproduce similar clusters throughout the year, while HR does not show consistent clustering patterns.


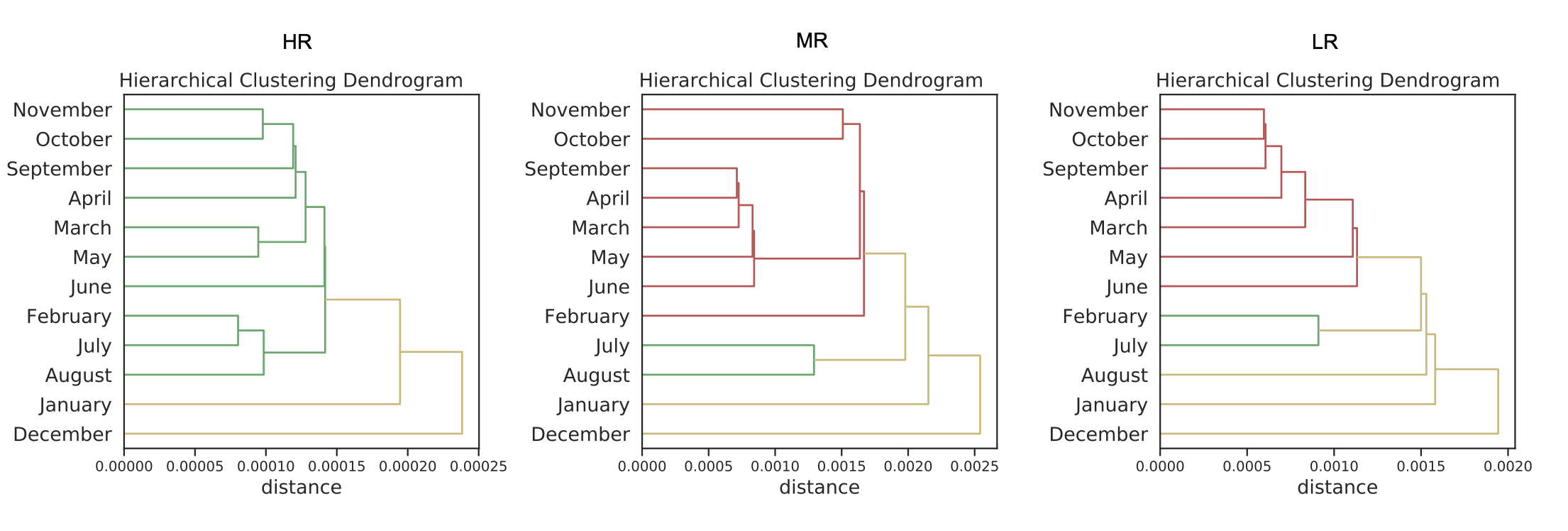


**Fig C in S1 Text.** Hierarchical clustering Dendrogram in HR, MR, LR. Colors represent the resulting clusters across all months.

To complement the MRQAP correlation analysis and further quantify the structural differences between coupling networks constructed with methods HR, MR, and LR, we computed four standard network centrality metrics for all 394 municipalities (**Fig D in S1 Text**). These metrics provide quantitative measures of node importance and network topology that have direct epidemiological interpretation: degree centrality identifies the number of direct pathways for disease introduction; closeness centrality measures how quickly infections can spread from a location to all others; betweenness centrality identifies critical bridge locations whose removal would fragment transmission pathways; and PageRank captures the hierarchical importance of locations considering both direct connections and connections to other important nodes. The centrality analysis reveals the three aggregation methods produce networks with fundamentally different structural properties. Method HR shows lower degree (median=0.50) and closeness centrality (median=0.66) compared to MR (degree median=0.85, closeness median=0.87) and LR (degree median=0.76, closeness median=0.80), indicating a fragmented network with longer effective distances. While median betweenness centrality is comparable across methods (~0.0004), HR exhibits extreme values (maximum ~0.0030) for certain nodes—more than double the maximum observed in MR and LR (maximum ~0.0015)—indicating artificial bottlenecks. PageRank shows similar medians across all methods (~0.0025), but HR displays a dramatically narrower distribution (IQR: 0.0024-0.0026) compared to MR (IQR: 0.0023-0.0027) and LR (IQR: 0.0023-0.0027), indicating failure to capture network hierarchy. In contrast, methods MR and LR show similar centrality distributions with higher degree and closeness values, moderate betweenness without extreme outliers, and heterogeneous PageRank distributions reflecting realistic hierarchical structure.


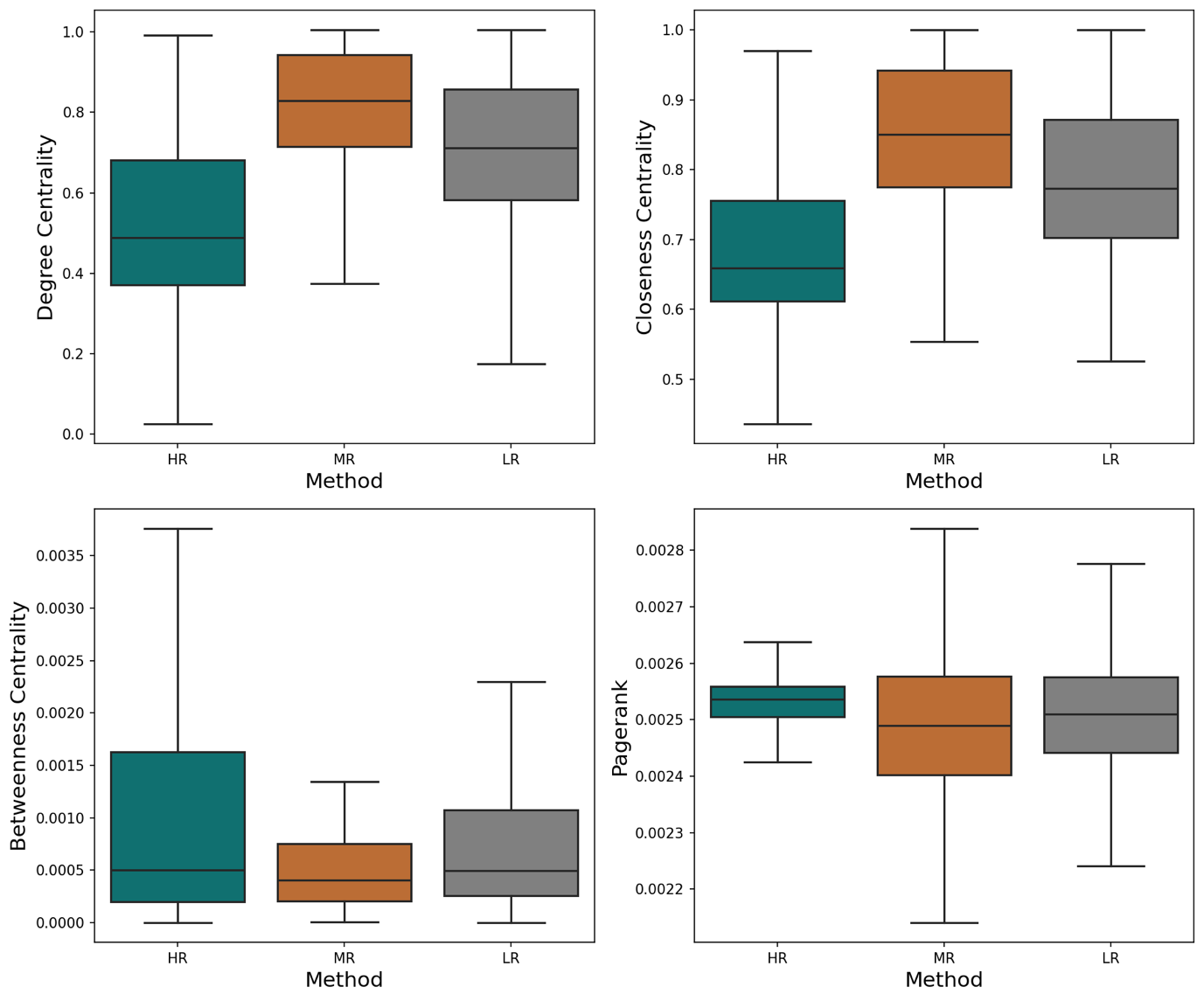


**Fig D in S1 Text.** Network centrality metrics comparison across HR, MR, LR. Box plots showing the network centrality metrics across all 394 municipalities in the daily average coupling networks for each month in 2013. a) Degree centrality. b) Closeness centrality. c) Betweenness centrality. d) PageRank. Box plots indicate median and 95% CI.

As **Fig E in S1 Text** shows, network metrics remained stable throughout the year, demonstrating that our findings are not driven by seasonal variations. Link density varied minimally across months, with HR consistently showing the lowest connectivity (43-48%), MR the highest (68-73%), and LR intermediate values (60-65%), maintaining the hierarchical ordering HR < LR <MR across all months. The degree distributions and self-connection probability ($p_{ii}$) showed consistent patterns month-to-month, with MR and LR maintaining stable $p_{ii}$ values (0.6-0.8) while D exhibited slightly higher and more variable values (0.75-0.95). Most importantly, the order-of-magnitude differences in inter-connection probability ($p_{ij}$) persisted across all months, with HR consistently showing lower coupling probabilities (median ~10^-6^) compared to MR and LR (median ~10^-4^ to 10^-3^), confirming that the differences between methods represent fundamental characteristics of each aggregation approach rather than temporal fluctuations.


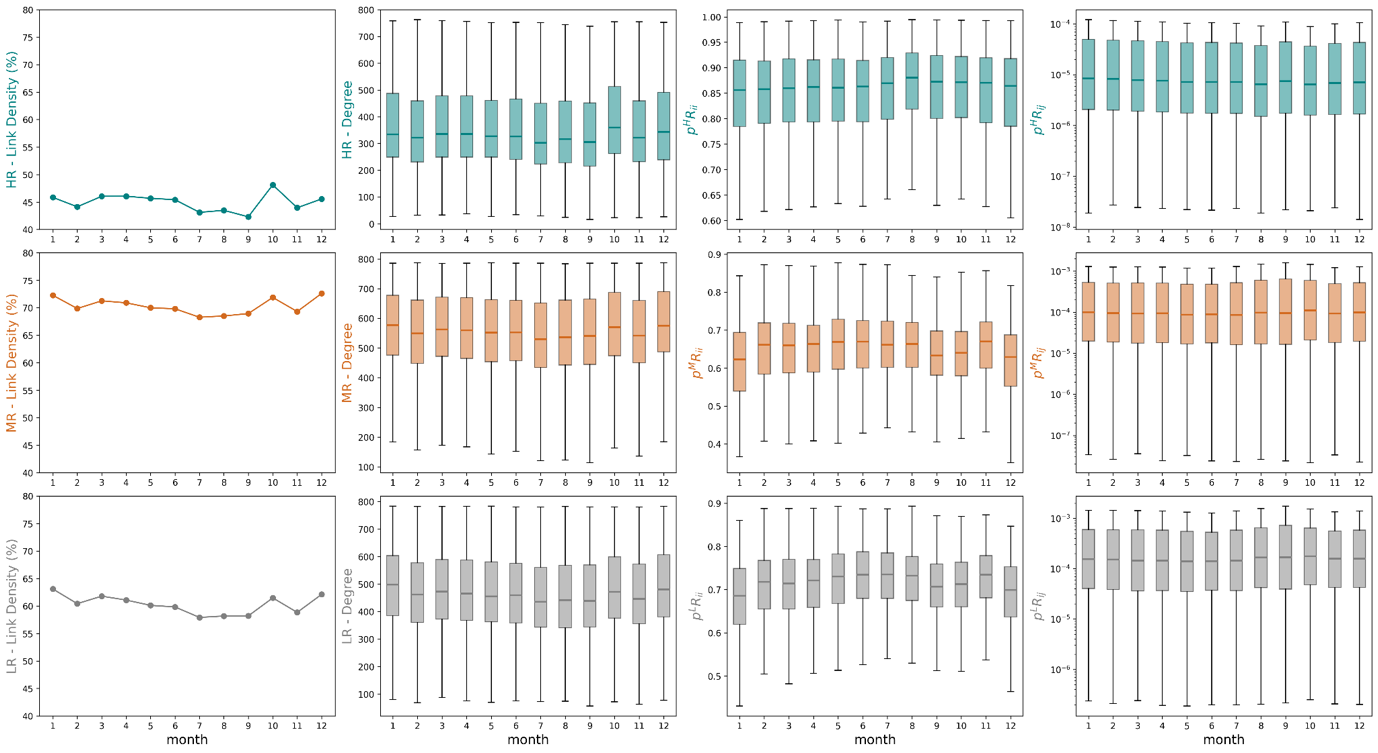


**Fig E in S1 Text:** Monthly-aggregated, daily-normalized coupling matrices for three mobility aggregation methods extracted from mobile phone data in Senegal, January-December 2013. Each row corresponds to one aggregation method (top: HR, middle: MR, bottom: LR). Columns display four network properties: **(Left)** Link density (%), calculated as the percentage of realized connections among all possible municipality pairs, with each point representing one month; **(Second column)** Degree distribution showing the number of connections per municipality, displayed as box plots (whiskers: 5th-95th percentile, box: 25th-75th percentile, line: median) for each month; **(Third column)** Self-connection probability ($\rho_{ii}$) distribution, representing the probability that individuals remain in their home municipality; **(Right)** Inter-connection probability ($\rho_{ij}$) distribution on logarithmic scale, representing coupling strength between different municipalities.

# **The metapopulation model**

The discrete-time SEIR model has the following form:

$$S_{i}\left( t+1 \right)=\left( 1-\lambda_{i} \right)S_{i}\left( t \right)$$

$$E_{i}\left( t+1 \right)=\left( 1-\epsilon\right)E_{i}\left( t \right)+\lambda_{i}S_{i}\left( t \right)$$

$$I_{i}\left( t+1 \right)=\left( 1-\mu\right)I_{i}\left( t \right)+\epsilon E_{i}\left( t \right)$$

$$R_{i}\left( t+1 \right)=R_{i}\left( t \right)+\mu I_{i}\left( t \right)$$

$\lambda_{i},\epsilon$, $\mu$ are the force of infection in a subpopulation $i$, the incubation rate and the recovery rate respectively. $S_{i}\left( t \right),E_{i}\left( t \right),I_{i}\left( t \right),R_{i}\left( t \right),$ denote the number of susceptible, exposed, infected and recovered individuals at time *t*. For every $i,N_{i}=S_{i}+E_{i}+I_{i}+R_{i}$ where $N_{i}$ is the number of residents in the municipality $i.$

Simulation details

Simulations were initialized with 10 infected individuals in a single seed location. We performed simulations for 92 epidemic seeds: all 46 urban municipalities and the top 10% of rural locations with the highest variation in outgoing and coupling probability between methods D and L. We set the average incubation period to $\epsilon^{-1}=1.5$ days and the average infectious period to $\mu^{-1}=3$ days. Stochastic simulations used a 1-day time step. For each scenario and seed location, we performed 1,000 simulation runs.

# **Epidemic simulations**


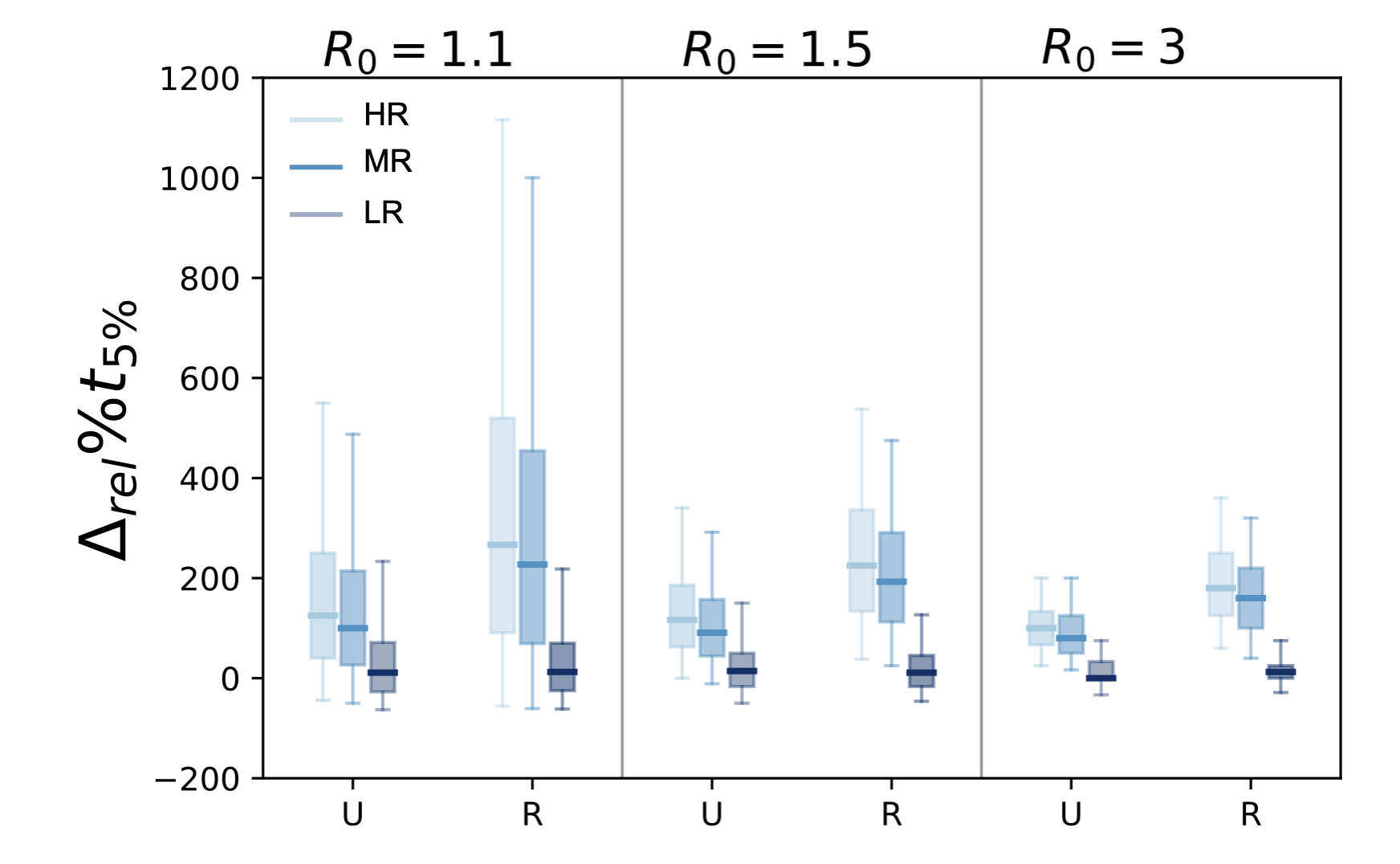


**Fig F in S1 Text.** Relative variation of $t_{5\%}.$ Box plots ranging from 5th to 95th percentile.

To better understand the differences between HR, MR, LR in the early-stage epidemics, here we present additional results on the time when the 5% of locations have been infected (**Fig F in S1 Text**). As shows **Fig F in S1 Text** the relative variation on $t_{5\%}$between MR and LR do not exceed 200% and the median is equal to 0, while between HR, MR and HR, LR it ranges from -100% to 1200%, with a median around 100%.

# **Complementary analysis**

For completeness, we present the complementary analysis relative to the main manuscript, showing all the epidemic parameters analyzed.

**
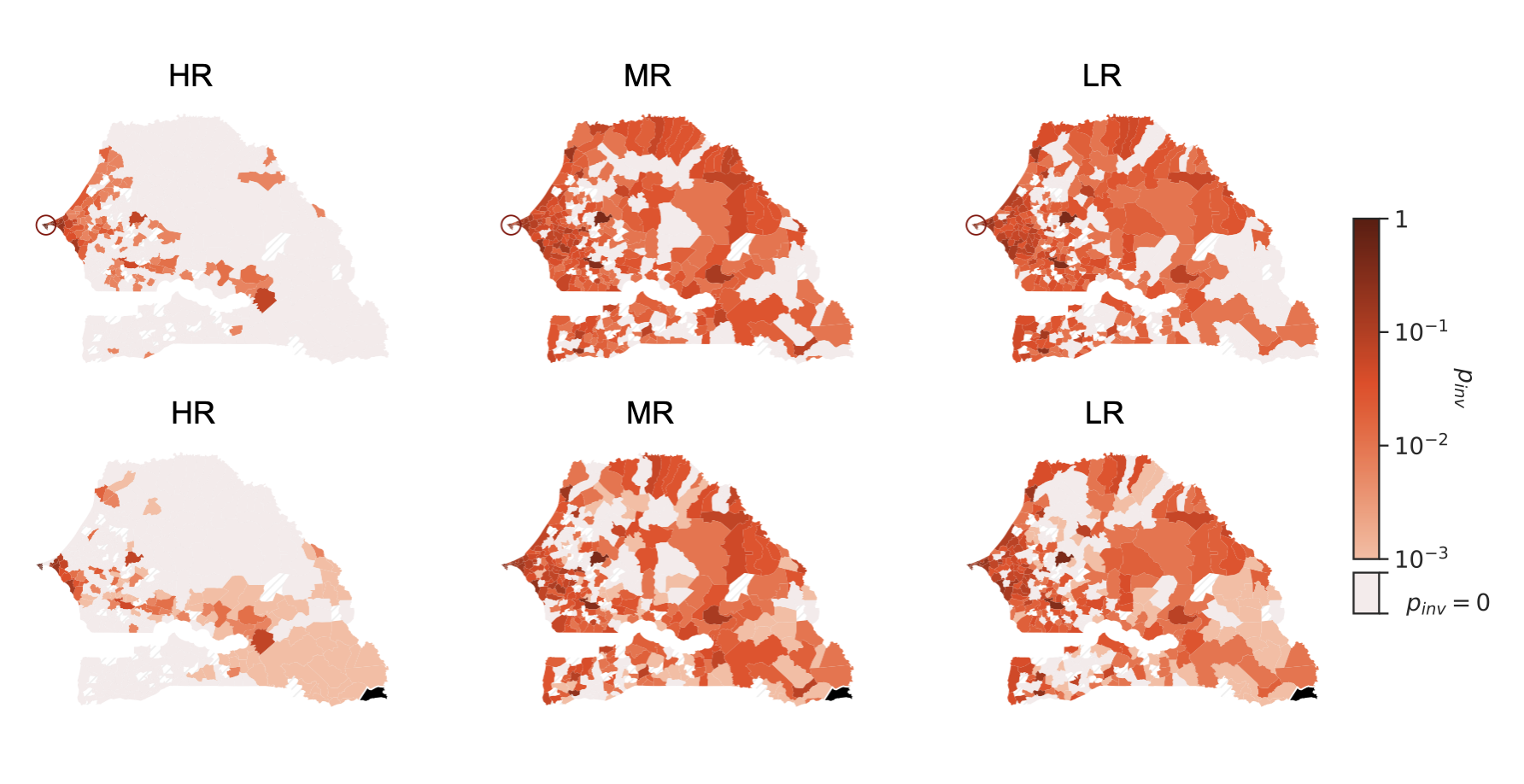
 Fig G in S1 Text.**  Map of the invasion probability at $t_{5\%}$ in each municipality with $R_{0}=1.1$. The municipality in black is the epidemic seed. The maps were generated in Python using administrative boundary shapefiles from the Global Administrative Areas database (GADM), available at <https://gadm.org>.


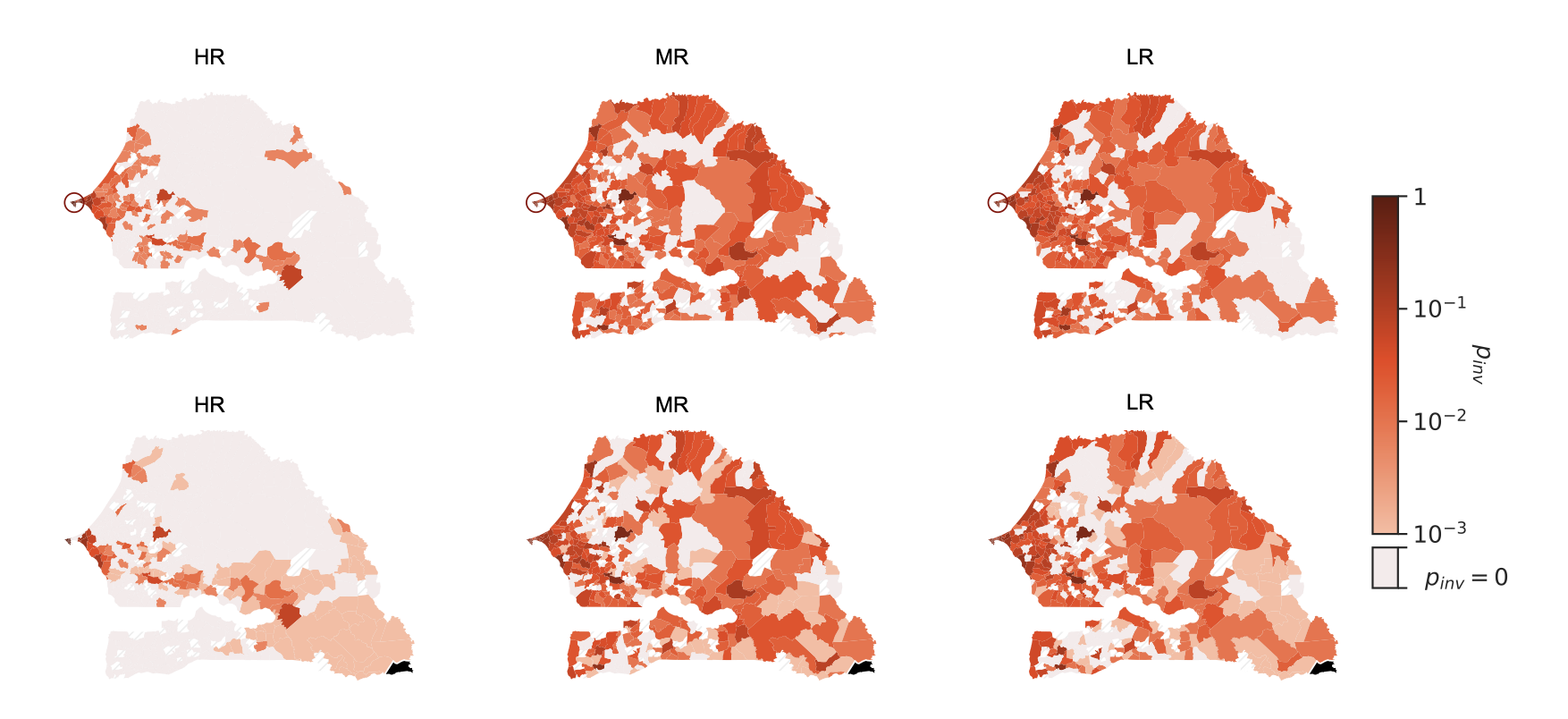


**Fig H in S1 Text.**  Map of the invasion probability at $t_{5\%}$ in each municipality with $R_{0}=1.5$. The municipality in black is the epidemic seed. The maps were generated in Python using administrative boundary shapefiles from the Global Administrative Areas database (GADM), available at <https://gadm.org>.

**
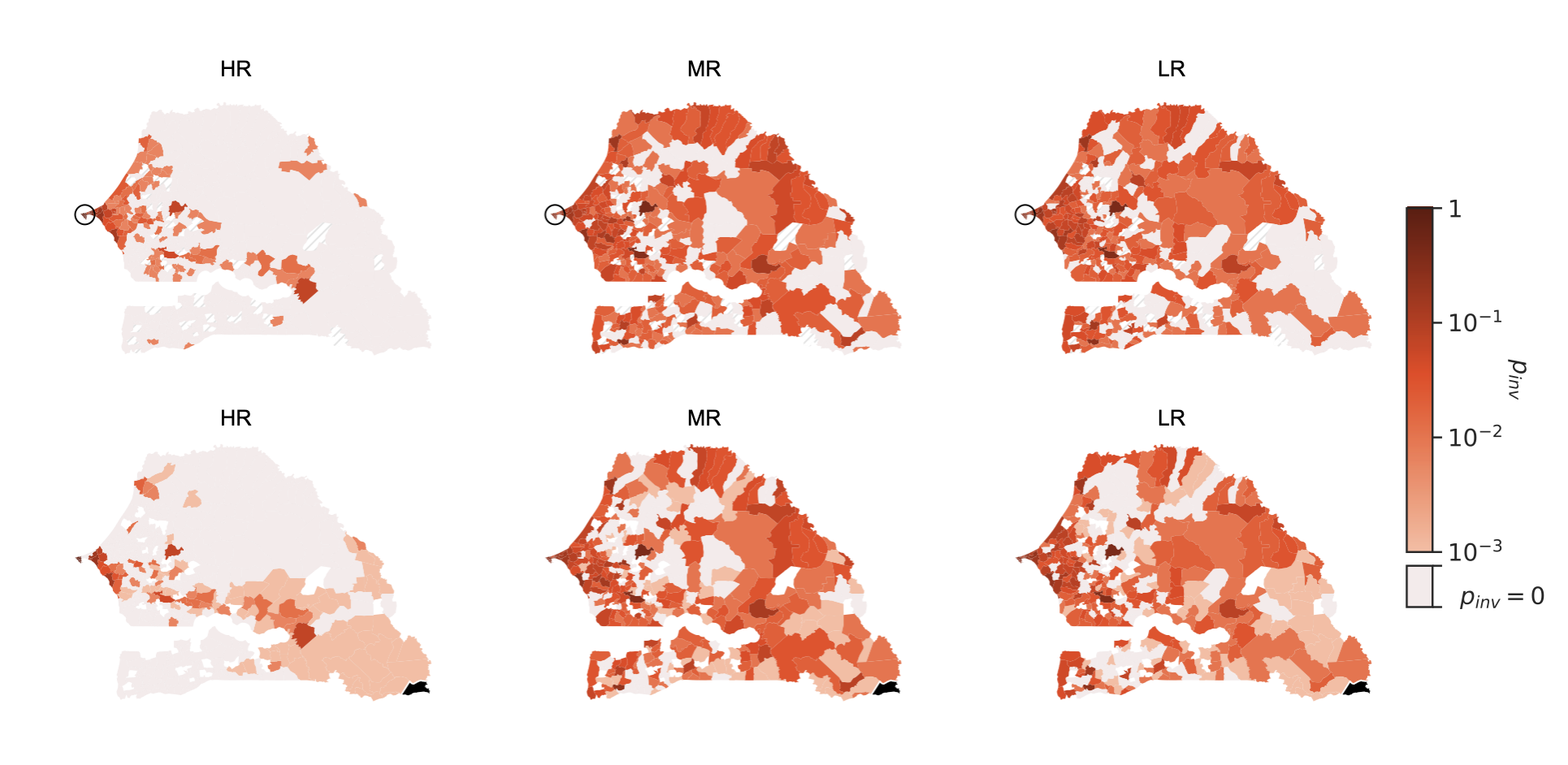
 Fig I in S1 Text.**  Map of the invasion probability at $t_{5\%}$ in each municipality with $R_{0}=3$. The municipality in black is the epidemic seed. The maps were generated in Python using administrative boundary shapefiles from the Global Administrative Areas database (GADM), available at <https://gadm.org>.


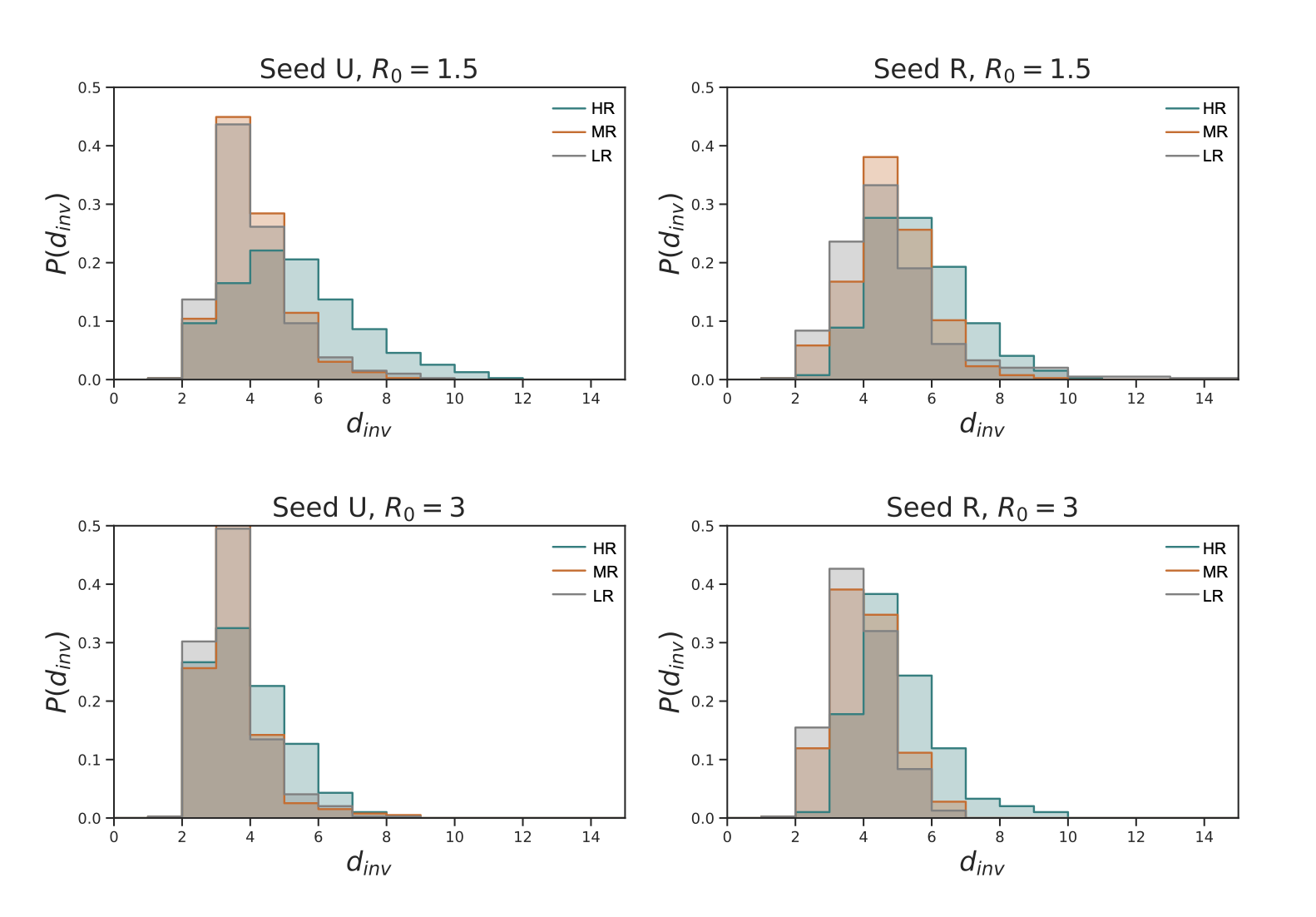


**Fig J in S1 Text.**  Invasion distance distributions in HR, MR and LR.

**
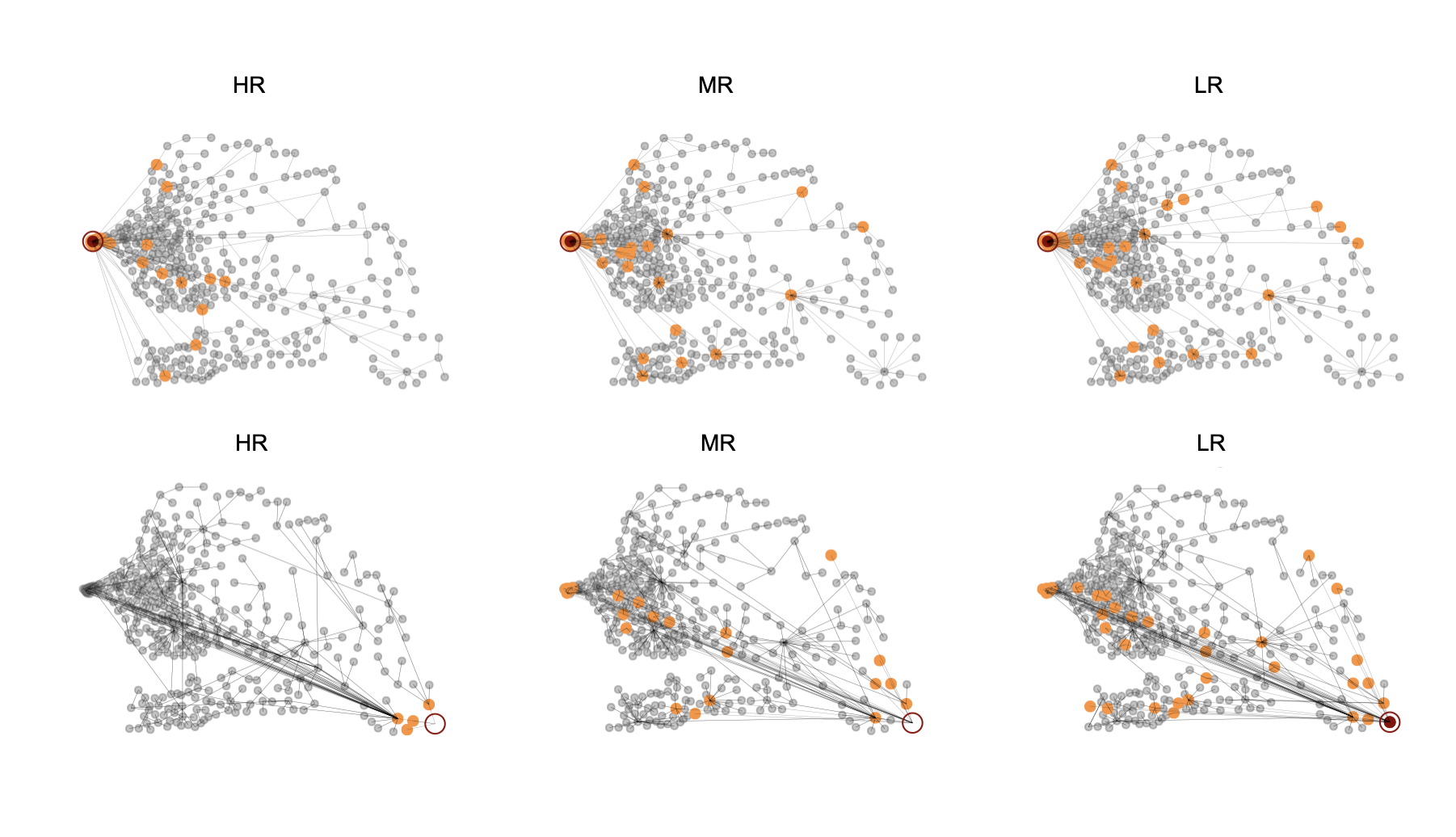
 Fig K in S1 Text.**  Invasion trees in HR, MR, LR at $R_{0}=1.5$. The red dot indicates the epidemic seed, and the orange dots indicate the municipalities directly infected by the seed. The maps were generated in Python using administrative boundary shapefiles from the Global Administrative Areas database (GADM), available at <https://gadm.org>.


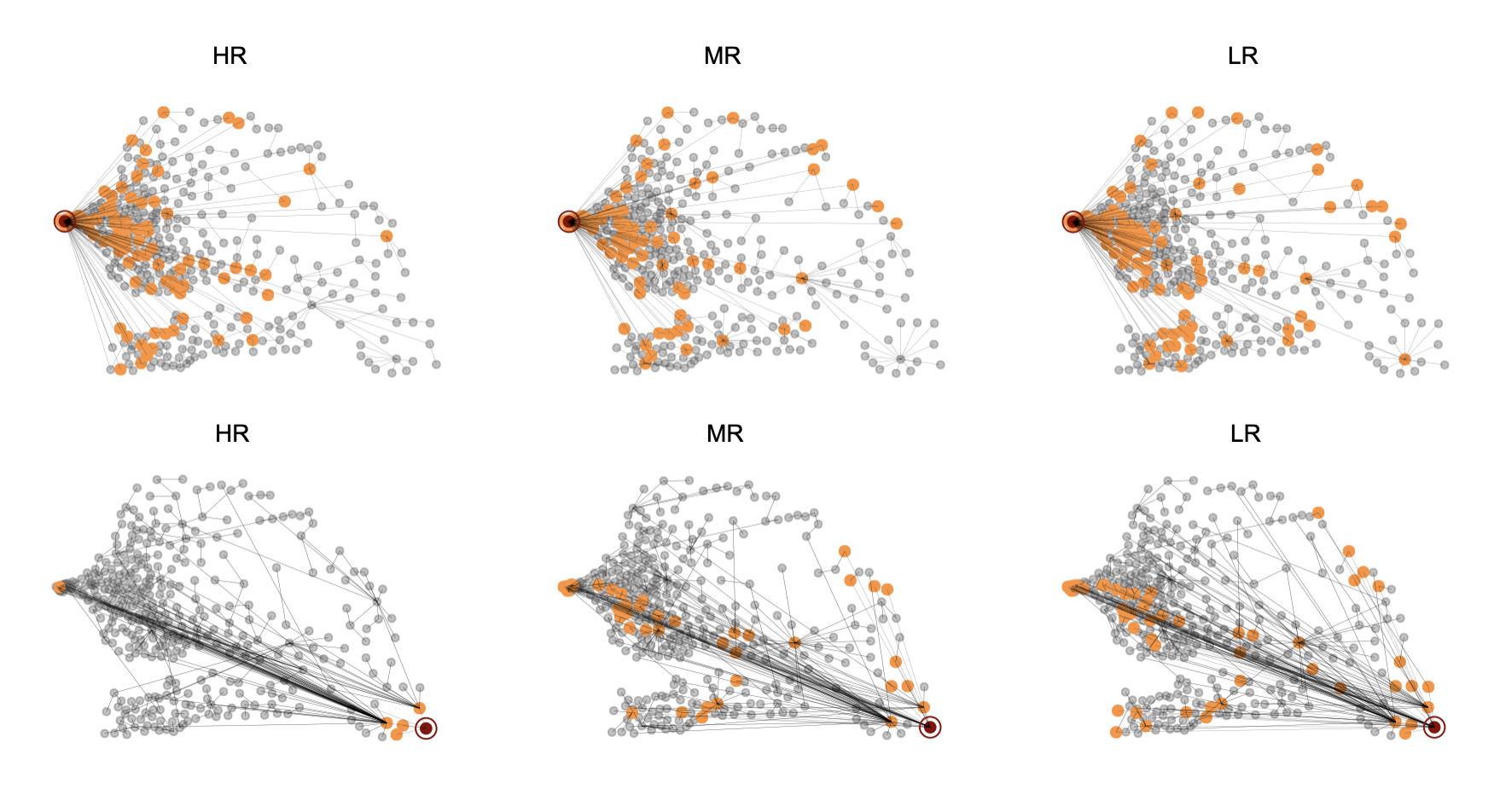


**Fig L in S1 Text.**  Invasion trees in HR, MR, LR at $R_{0}=3$. The red dot indicates the epidemic seed, and the orange dots indicate the municipalities directly infected by the seed. The maps were generated in Python using administrative boundary shapefiles from the Global Administrative Areas database (GADM), available at <https://gadm.org>.

**
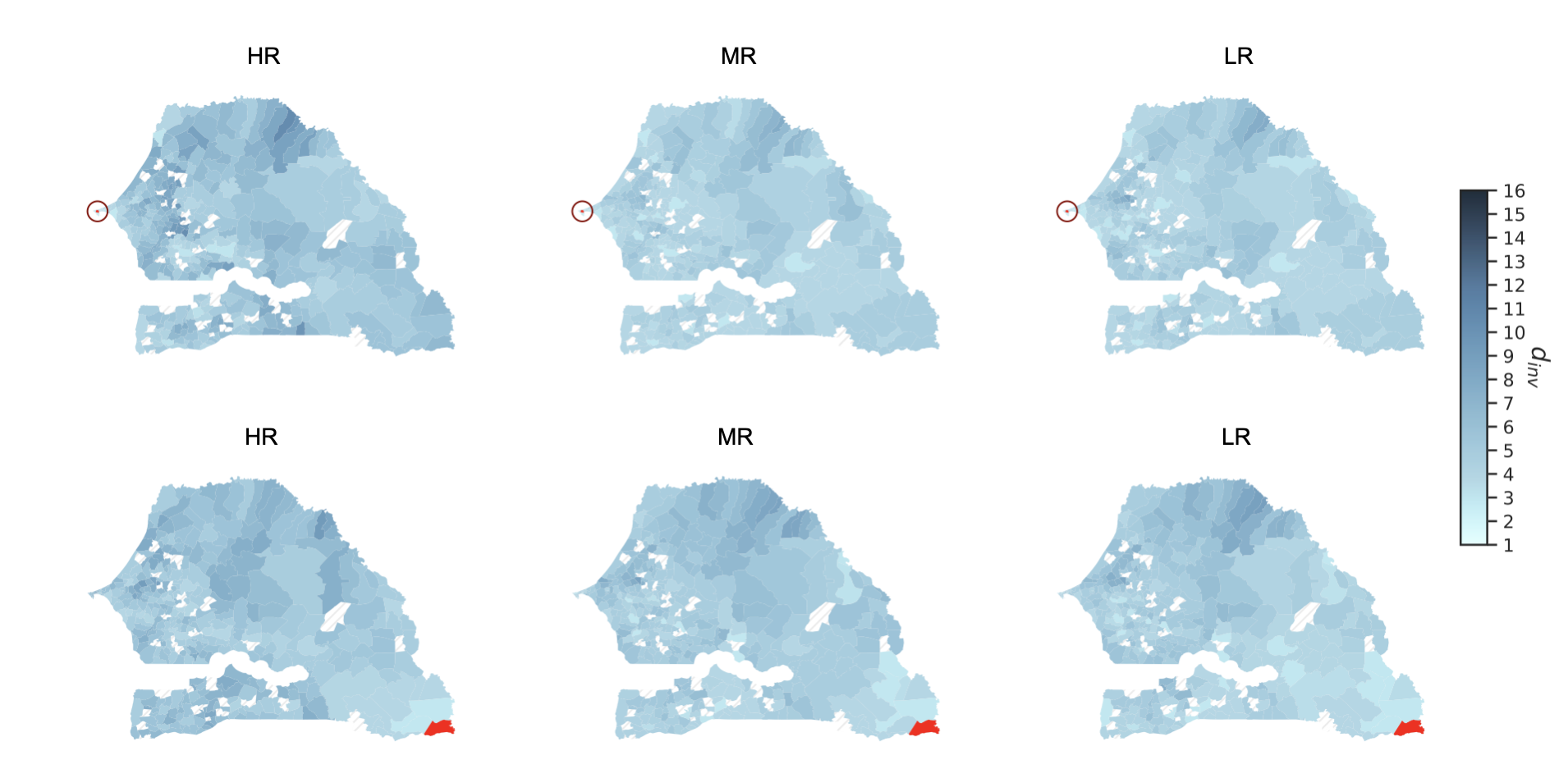
 Fig M in S1 Text.** Visualization on map of the invasion distance at$R_{0}=1.5$in HR, MR, LR. The red municipality is the epidemic seed. The maps were generated in Python using administrative boundary shapefiles from the Global Administrative Areas database (GADM), available at <https://gadm.org>.


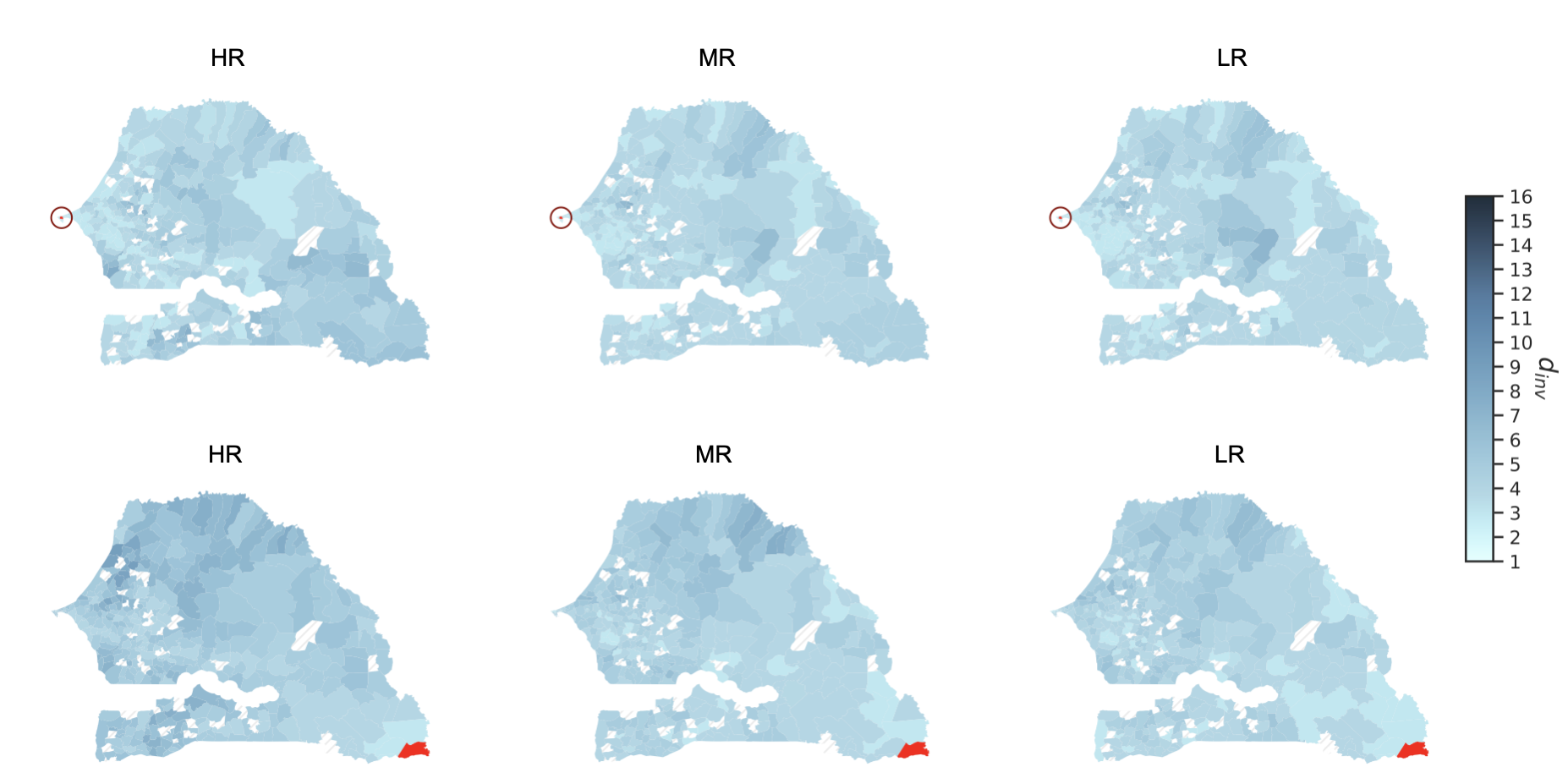


**Fig N in S1 Text.** Visualization on map of the invasion distance at $R_{0}=3$in HR, MR, LR. The red municipality is the epidemic seed. The maps were generated in Python using administrative boundary shapefiles from the Global Administrative Areas database (GADM), available at <https://gadm.org>.

# **Sensitivity analysis on HR, MR, LR**

## Corrected high-resolution matrix (HR’)

Following Lima et al.^2^, we defined a time-weighted variant:

$$p_{ij}^{HR'}=\frac{\sum_{u_{i}} HR'_{i,j}^{u}}{\sum_{k} \sum_{u_{i}} HR'_{i,k}^{u}}$$

where $HR'_{i,j}^{u}$ is the sum of time elapsed during displacements between $i$ and *j* by user *u*. For each pair of consecutive calls at locations $i$ and *j* with timestamps $t_{1}$ and $t_{2}$, we attribute the time difference $\left( t_{2}-t_{1} \right)$ to the displacement from $i$ to *j*.

This variant aims to correct the bias in D by accounting for the duration of movements, giving more weight to displacements that take longer to complete.

## Normalized high-resolution matrix ($\mathrm{HR}_{\mathrm{norm}}$)

To address heterogeneity in user activity patterns, we introduced an activity-normalized version of the displacement matrix:

$$p_{ij}^{{HR}_{norm}}=\frac{\sum_{u_{i}} {HR}_{norm,i,j}^{u}}{\sum_{k} \sum_{u_{i}} {HR}_{norm,i,k}^{u}}$$

where ${HR}_{norm,i,j}^{u}=\frac{{HR}_{i,j}^{u}}{N_{u}}$ and $N_{u}$ is the total number of activities (calls + SMS) by user *u*.

In the standard HR matrix, users with higher activity rates contribute disproportionately to coupling probabilities. Normalization by total activity ensures that each user's contribution is weighted proportionally to their overall mobile phone usage, thereby reducing bias from highly active users.

## Corrected medium-resolution matrix (${MR'})$

To more accurately represent time allocation for each user trajectory, we compute the actual time spent at each location rather than using the number of activities as in MR. We calculate time spent as follows:

1. For consecutive calls at different locations (call at location $i$ at time $t_{1}$, then then at location *j* at time $t_{2}$):
   - Time at origin $i$: $\left( t_{2}-t_{1} \right)/2$
   - Time at destination *j*: $\left( t_{2}-t_{1} \right)/2$;
2. For consecutive calls at the same location (both calls at location $i$ at times $t_{1}$ and $t_{2}$):
   - Time spent at $i$: $\left( t_{2}-t_{1} \right)$

The coupling probability is then defined as:

$$p_{ij}^{MR'}=\frac{\sum_{u_{i}} MR'_{i,j}^{u_{i}}}{\sum_{k} \sum_{u_{i}} MR'_{i,k}^{u_{i}}}$$

where $MR'_{i,j}^{u_{i}}$ is the actual time spent in location *j* by user $u_{i}$ living in $i$.

This method eliminates the assumption that call frequency is proportional to time spent, instead directly measuring the duration at each location. This correction is particularly important for addressing nighttime bias: while users make few calls during nighttime hours, they spend substantial time at home during this period. By measuring actual time rather than call frequency, *MR'* provides a more accurate representation of time allocation across locations.

## Corrected low-resolution matrix (${LR'})$

$$p_{ij}^{LR'}=\frac{\sum_{u_{i}} LR'_{i,j}^{u_{i}}}{\sum_{k} \sum_{u_{i}} LR'_{i,k}^{u_{i}}}$$

where $LR'_{i,j}^{u_{i}}$ is determined by identifying the most visited location during daytime hours (7am-7pm) only, rather than over the full 24-hour period as in LR.

By restricting the time window to daytime hours, we exclude nighttime periods when users are predominantly at home. This modification increases the probability that the most visited location represents the workplace or school rather than the home location. Consequently, LR*'* more accurately captures epidemiologically relevant non-residential locations where social contact and potential disease transmission occur.

## Sensitivity analysis results (HR’, MR’, LR’)


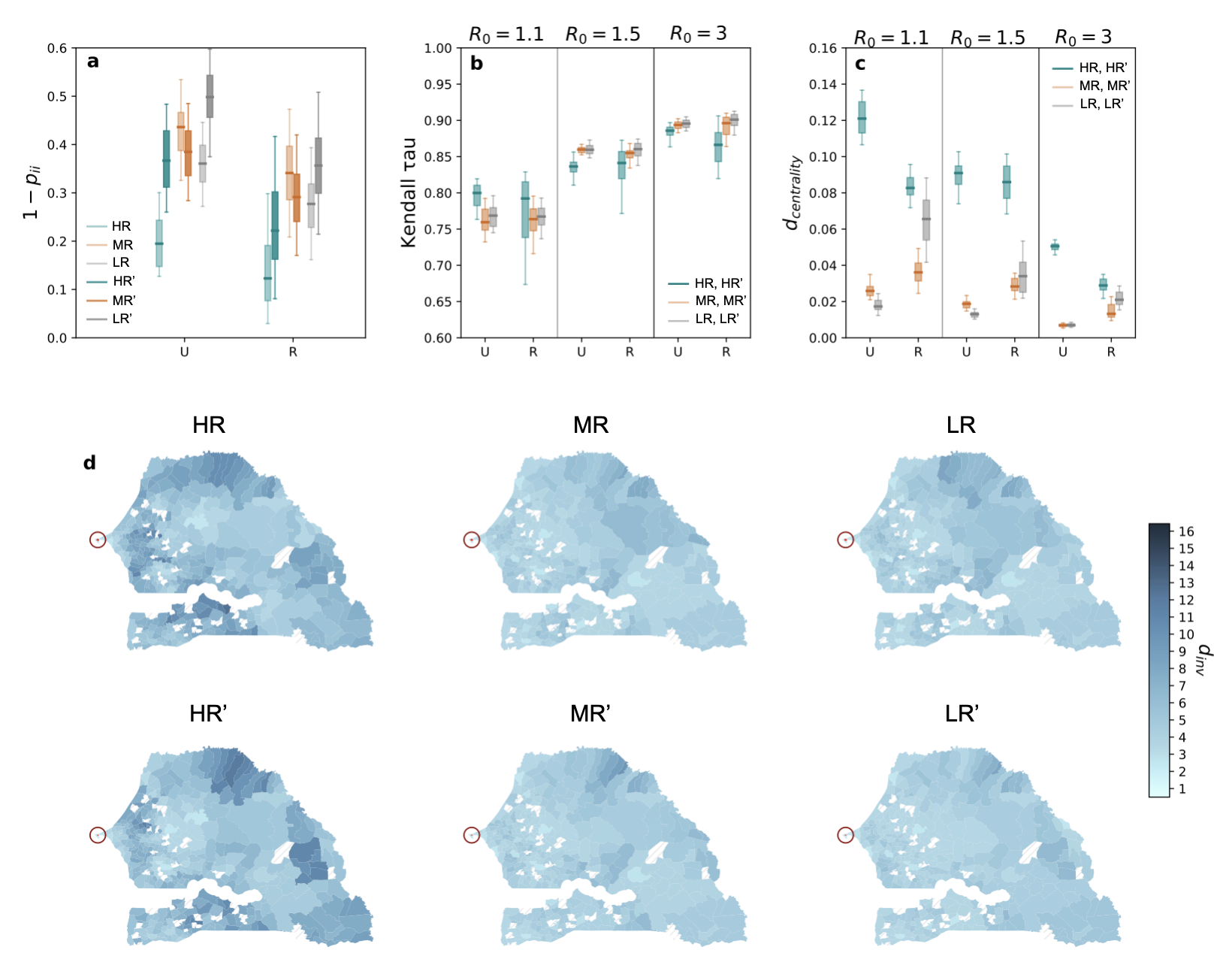


**Fig O in S1 Text. Sensitivity analysis. a)** Daily average outgoing probability for each month in 2013 in HR, MR, LR and HR', MR’, LR', stratified by Urban (U) and Rural (R) municipalities. **b)** Kendall tau correlation coefficient comparing HR vs. HR', MR vs. MR', and LR vs. LR', stratified by urban and rural seeds. **c)** Betweenness centrality distance index measured between epidemic invasion trees for each pair of methods. Box plots indicate the 95% reference range. Betweenness centrality is measured on invasion trees using one urban (U) and one rural (R) seed. **d)** Map visualization of epidemic invasion distance when the capital Dakar (red circle) is the epidemic seed and R₀ = 1.1. The maps were generated in Python using administrative boundary shapefiles from the Global Administrative Areas database (GADM), available at <https://gadm.org>. License information is available at <https://gadm.org/license.html>.

**
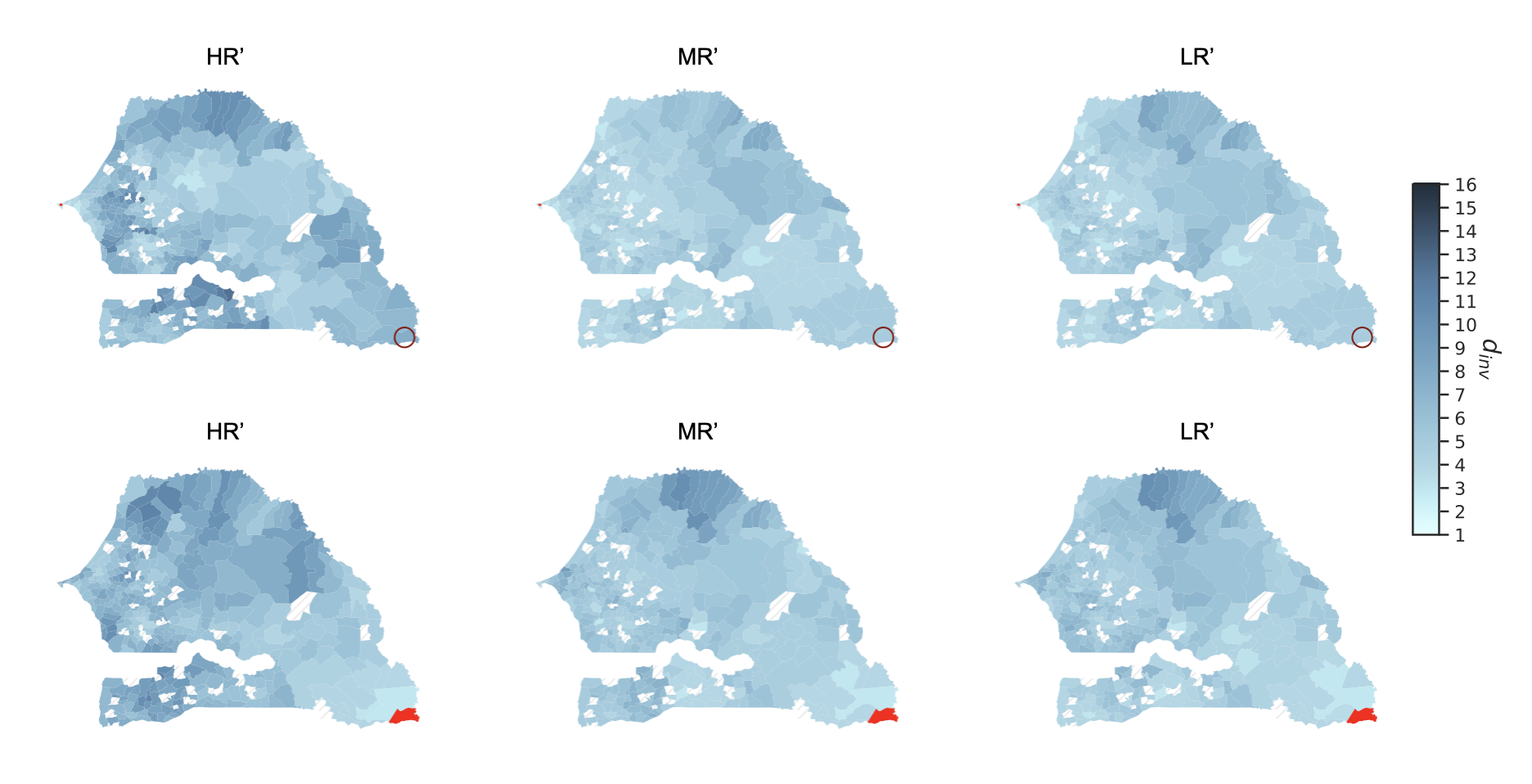
**

**Fig P in S1 Text.** Visualization on map of the invasion distance at $R_{0}=1.1$ in HR’, MR’, LR’. The red municipality is the epidemic seed. The maps were generated in Python using administrative boundary shapefiles from the Global Administrative Areas database (GADM), available at <https://gadm.org>. License information is available at <https://gadm.org/license.html>.

**
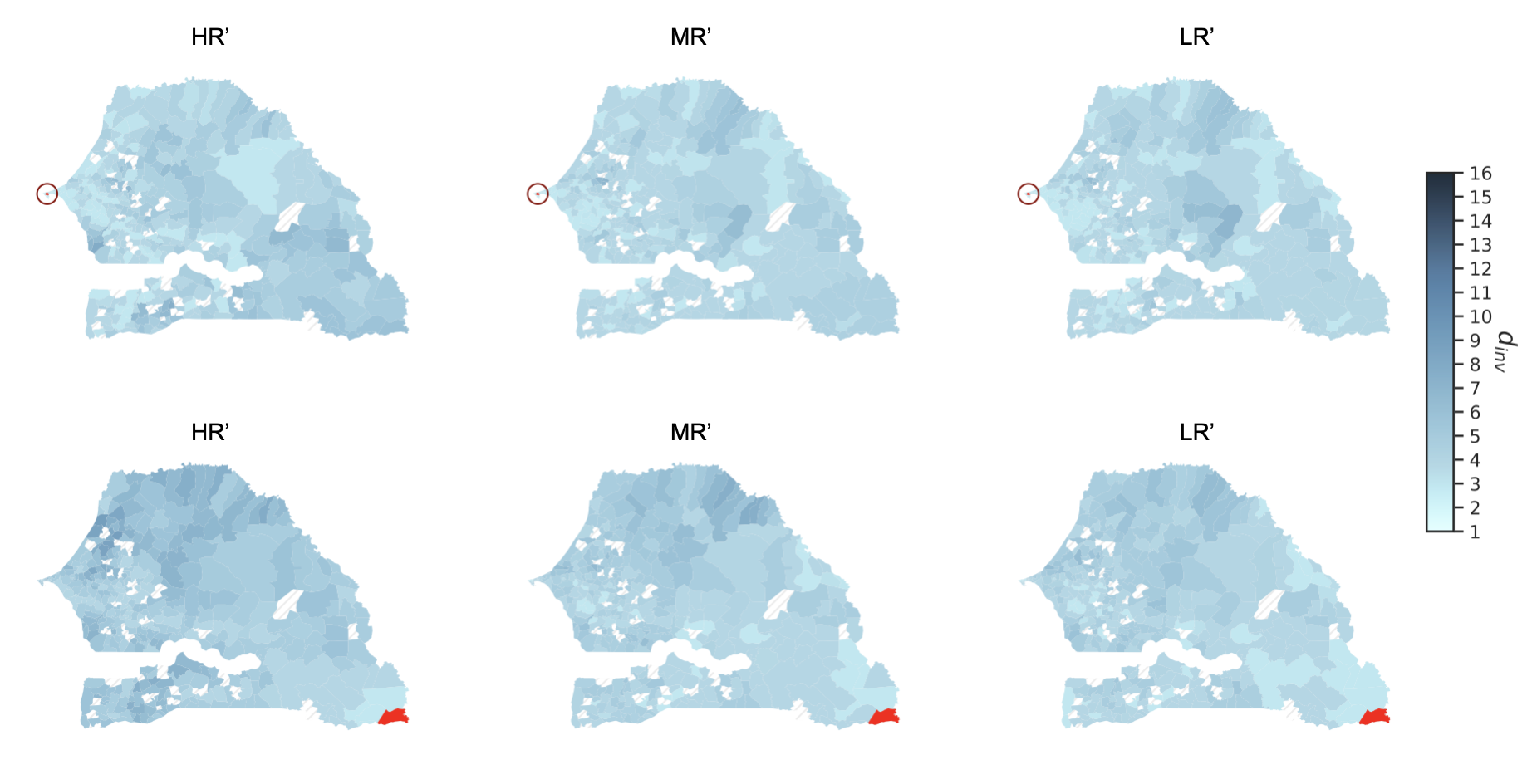
 Fig Q in S1 Text.** Visualization on map of the invasion distance at $R_{0}=1.5$ in HR’, MR’, LR’. The red municipality is the epidemic seed. The maps were generated in Python using administrative boundary shapefiles from the Global Administrative Areas database (GADM), available at <https://gadm.org>. License information is available at <https://gadm.org/license.html>.


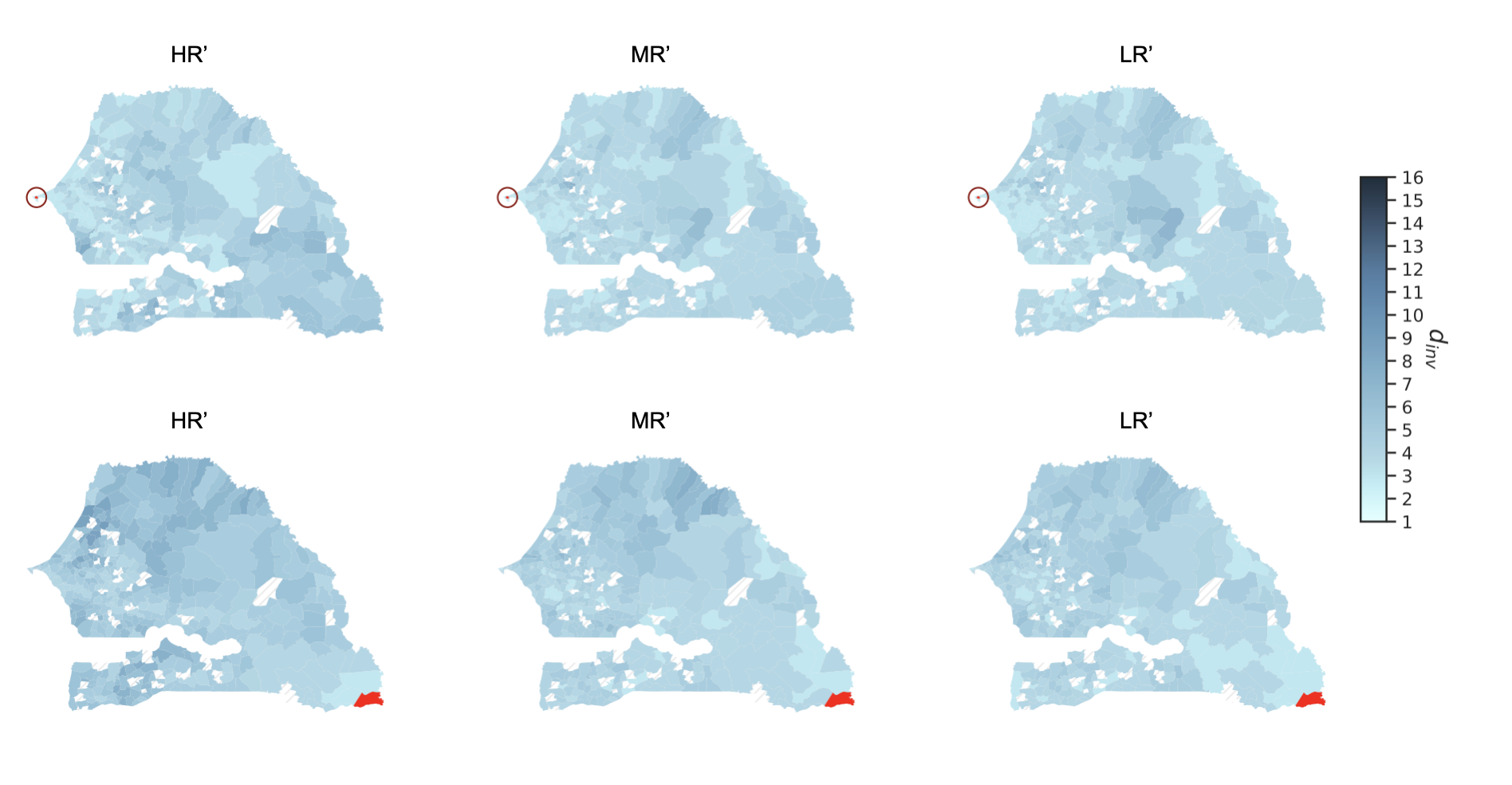


**Fig R in S1 Text.** Visualization on map of the invasion distance at $R_{0}=3$ in HR’, MR’, LR’. The red municipality is the epidemic seed. The maps were generated in Python using administrative boundary shapefiles from the Global Administrative Areas database (GADM), available at <https://gadm.org>. License information is available at <https://gadm.org/license.html>.

**
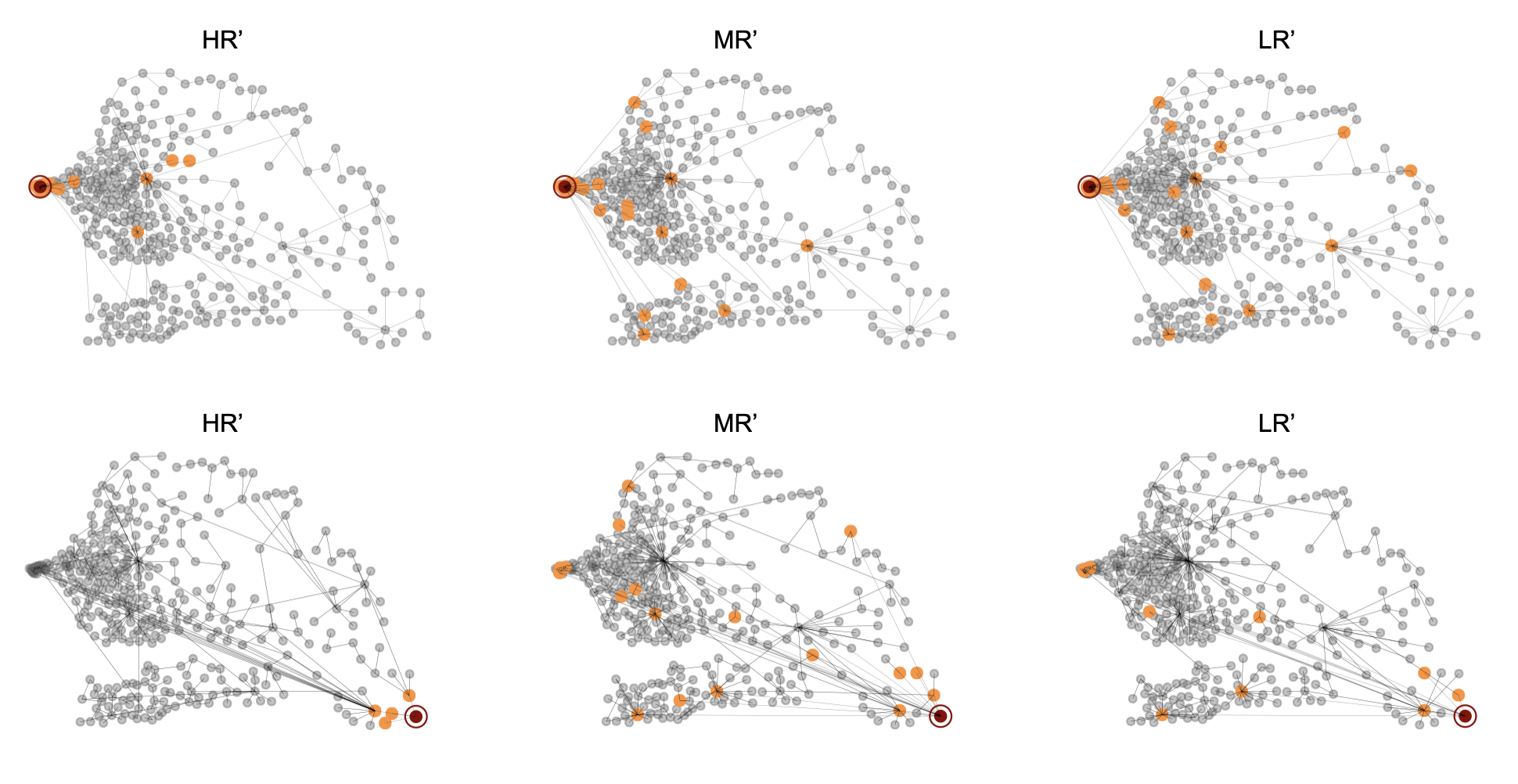
 Fig S in S1 Text.**  Invasion trees in HR’, MR’, LR’ at $R_{0}=1.1$. The red dot indicates the epidemic seed, and the orange dots indicate the municipalities directly infected by the seed. The maps were generated in Python using administrative boundary shapefiles from the Global Administrative Areas database (GADM), available at <https://gadm.org>. License information is available at <https://gadm.org/license.html>.

**
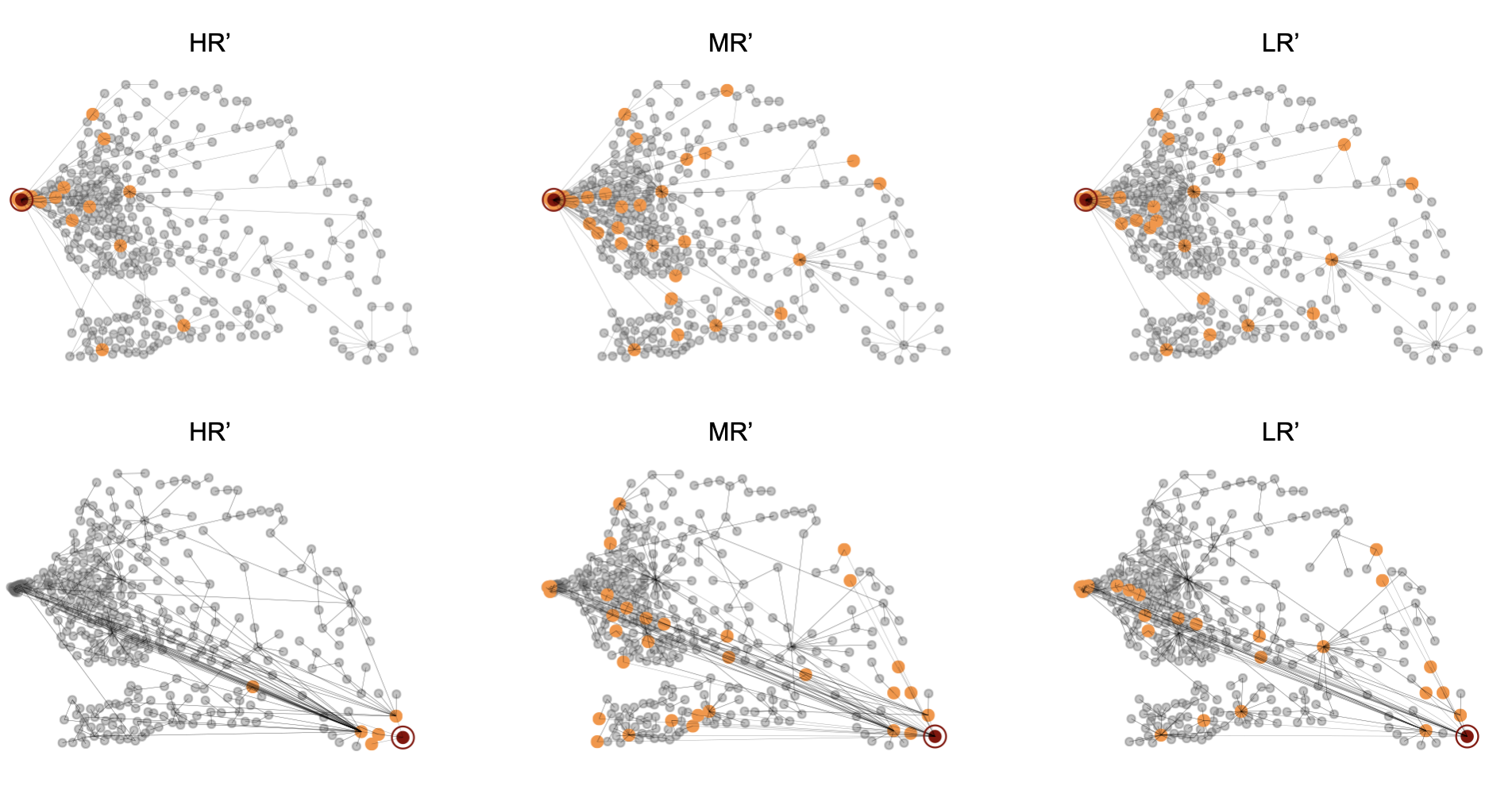
 Fig T in S1 Text.**  Invasion trees in HR’, MR’, LR’ at $R_{0}=1.5$. The red dot indicates the epidemic seed, and the orange dots indicate the municipalities directly infected by the seed. The maps were generated in Python using administrative boundary shapefiles from the Global Administrative Areas database (GADM), available at <https://gadm.org>. License information is available at <https://gadm.org/license.html>.

**
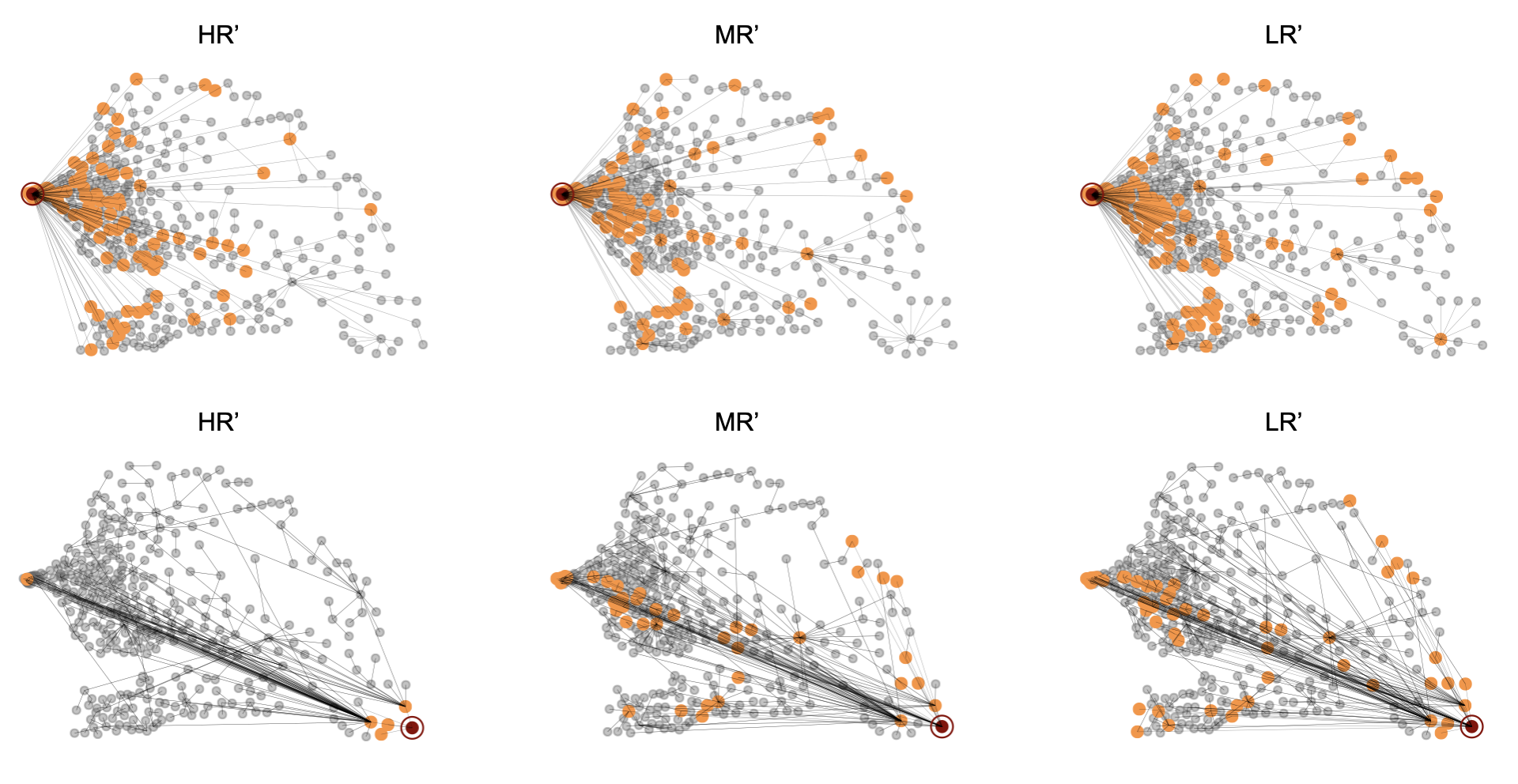
**

**Fig U in S1 Text.**  Invasion trees in HR’, MR’, LR’ at $R_{0}=3$. The red dot indicates the epidemic seed, and the orange dots indicate the municipalities directly infected by the seed. The maps were generated in Python using administrative boundary shapefiles from the Global Administrative Areas database (GADM), available at <https://gadm.org>. License information is available at <https://gadm.org/license.html>.

## Sensitivity analysis results ($\mathrm{HR}_{\mathrm{norm}}$)

We corrected the high-resolution matrix by defining $\mathrm{HR}_{\mathrm{norm}},$

which accounts for the heterogeneity of mobile phone users' activity patterns. Here we analyze the differences between HR and $\mathrm{HR}_{\mathrm{norm}}$ by comparing both the coupling matrices and the epidemic outcomes.

**
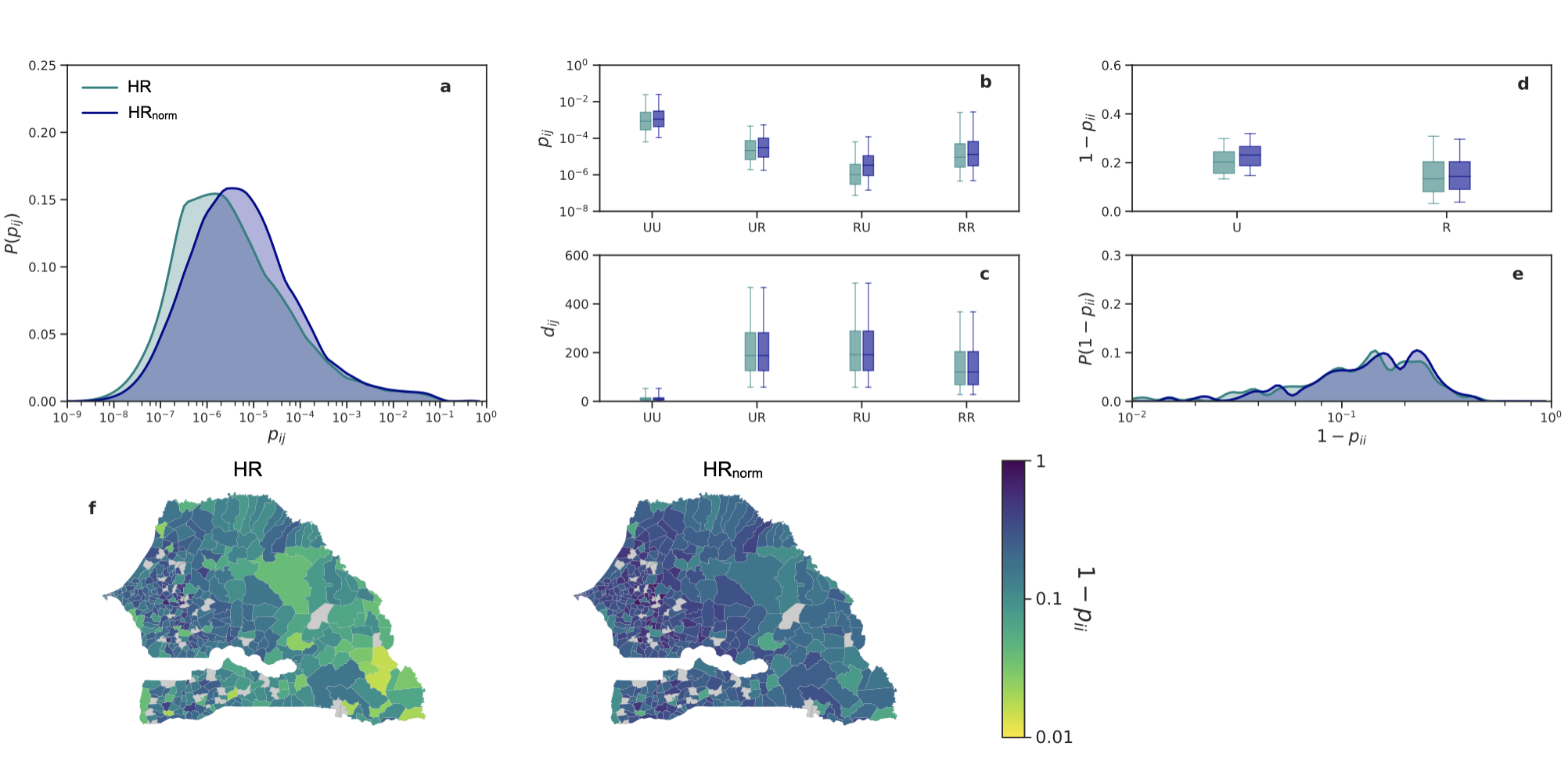
**

**Fig V in S1 Text.** **Differences between HR and** $\mathbf{HR}_{\mathbf{norm}}\boldsymbol{.}$**a)** Distribution of coupling probability in HR and $\mathrm{HR}_{\mathrm{norm}}$. **b), c), d)** Coupling probability, geographical distance, and outgoing probability distributions showing daily averages across all months in 2013, stratified by Urban (U) and Rural (R) municipalities. Box plots ranging from 5th to 95th percentile. **e)** Outgoing probability distribution in HR and $\mathrm{HR}_{\mathrm{norm}}$. **f)** Map visualization of outgoing probability in January for HR and $\mathrm{HR}_{\mathrm{norm}}$ for each municipality. The maps were generated in Python using administrative boundary shapefiles from the Global Administrative Areas database (GADM), available at <https://gadm.org>. License information is available at <https://gadm.org/license.html>.

# **References**

1. Wesolowski, A., Eagle, N., Noor, A. M., Snow, R. W. & Buckee, C. O. Heterogeneous Mobile Phone Ownership and Usage Patterns in Kenya. *PLoS ONE* **7**, (2012).
2. Lima, A., Pejovic, V., Rossi, L., Musolesi, M. & Gonzalez, M. Progmosis: Evaluating Risky Individual Behavior During Epidemics Using Mobile Network Data. *ArXiv150401316 Phys.* (2015).
