# Supplementary material for: Mobility data resolution needed to inform predictive models of spatial epidemic spread from mobile phone data
Source: PLoS Comput Biol. 2026 Jul 2;22(7):e1014427. doi: 10.1371/journal.pcbi.1014427 (PMC13327251; doi:10.1371/journal.pcbi.1014427)
Supplement: Attachment — Submitted filename: Response_to_reviewers.pdf [file pcbi.1014427.s003.pdf]

POINT-BY-POINT REPLY TO EDITOR AND REVIEWERS' REMARKS

Additional Editor Comments:

Please make all code and data available. If some data cannot be shared due to privacy issues, please explain.

We have made code and data publicly available on our GitHub repository at https://github.com/EPIcx-lab/Senegal-coupling. This repository includes:

- The monthly-aggregated, daily-normalized coupling matrices at municipality level (which do not contain individual-level information)
- The census data and administrative boundaries used in the study
- Implementation in C++ of the metapopulation epidemic model
- README file

Journal Requirements:

1) Please ensure that the CRediT author contributions listed for every co-author are completed accurately and in full. At this stage, the following Authors/Authors require contributions: Giulia Pullano, Shweta Bansal, Stefania Rubrichi, and Vittoria Colizza. Please ensure that the full contributions of each author are acknowledged in the "Add/Edit/Remove Authors" section of our submission form.
The list of CRediT author contributions may be found here: https://journals.plos.org/ploscompbiol/s/authorship#loc-author-contributions

We have updated the author contributions section as follows:
Giulia Pullano: Investigation, Data Curation, Formal analysis, Visualization, Methodology, Writing – original draft, Writing – review & editing
Shweta Bansal: Methodology, Writing – review & editing
Stefania Rubrichi: Data Curation, Resources, Funding acquisition, Methodology, Supervision, Writing – review & editing
Vittoria Colizza: Conceptualization, Funding acquisition, Project administration, Methodology, Supervision, Writing – review & editing

2) We ask that a manuscript source file is provided at Revision. Please upload your manuscript file as a .doc, .docx, .rtf or .tex. If you are providing a .tex file, please upload it under the item type u2018LaTeX Source Fileu2019 and leave your .pdf version as the item type u2018Manuscriptu2019.

We have uploaded the manuscript as a .docx file as requested.

3) Please provide an Author Summary. This should appear in your manuscript between the Abstract (if applicable) and the Introduction, and should be 150-200 words long. The aim should be to make your findings accessible to a wide audience that includes both scientists and non-scientists. Sample summaries can be found on our website under Submission Guidelines:
https://journals.plos.org/ploscompbiol/s/submission-guidelines#loc-parts-of-a-submission

We have added the Author Summary between the Abstract and Introduction.

4) Please upload all main figures as separate Figure files in .tif or .eps format. For more information about how to convert and format your figure files please see our guidelines:
https://journals.plos.org/ploscompbiol/s/figures

We have uploaded all main figures (Figures 1-6) as separate .tif files as per PLOS requirements. Each figure file is named according to its figure number (e.g., Figure1.tif, Figure2.tif, etc.).

5) We have noticed that you have uploaded Supporting Information files, but you have not included a list of legends. Please add a full list of legends for your Supporting Information files after the references list.

We have added the legend for the SI file in the main paper after the reference list.

6) We note that your Data Availability Statement is currently as follows: "no". Please confirm at this time whether or not your submission contains all raw data required to replicate the results of your study. Authors must share the “minimal data set” for their submission. PLOS defines the minimal data set to consist of the data required to replicate all study findings reported in the article, as well as related metadata and methods (https://journals.plos.org/plosone/s/data-availability#loc-minimal-data-set-definition).
For example, authors should submit the following data:
- The values behind the means, standard deviations and other measures reported;
- The values used to build graphs;
- The points extracted from images for analysis..
Authors do not need to submit their entire data set if only a portion of the data was used in the reported study.
If your submission does not contain these data, please either upload them as Supporting Information files or deposit them to a stable, public repository and provide us with the relevant URLs, DOIs, or accession numbers. For a list of recommended repositories, please see https://journals.plos.org/plosone/s/recommended-repositories.
If there are ethical or legal restrictions on sharing a de-identified data set, please explain them in detail (e.g., data contain potentially sensitive information, data are owned by a third-party organization, etc.) and who has imposed them (e.g., an ethics committee). Please also provide contact information for a data access committee, ethics committee, or other institutional body to which data requests may be sent. If data are owned by a third party, please indicate how others may request data access.

We have included our Data Availability Statement in the main manuscript as follows:
"The code and data for all analyses are publicly available at https://github.com/EPIcx-lab/Senegal-coupling. This repository includes monthly-aggregated, daily-normalized coupling matrices HR, MR, and LR at municipality level for each month in 2013, and the metapopulation model code, with census data, and Senegalese shapefiles used to design the model.”

7) Please amend your detailed Financial Disclosure statement. This is published with the article. It must therefore be completed in full sentences and contain the exact wording you wish to be published.
- State the initials, alongside each funding source, of each author to receive each grant. For example: "This work was supported by the National Institutes of Health (####### to AM; ###### to CJ) and the National Science Foundation (###### to AM)."
- State what role the funders took in the study. If the funders had no role in your study, please state: "The funders had no role in study design, data collection and analysis, decision to publish, or preparation of the manuscript.".
If you did not receive any funding for this study, please simply state: the authors received no specific funding for this work

We have amended the Financial Disclosure Statement to read: “This study was partially funded by: Agence Nationale de la Recherche project DATAREDUX (ANR-19-CE46-0008-03 to VC); Horizon Europe grants ESCAPE (101095619 to VC) and VERDI (101045989 to VC); EU Horizon 2020 grant MOOD (H2020-874850 to VC). G.P. was also supported by the Fritz-Family fellowship program from Georgetown University. The funders had no role in study design, data collection and analysis, decision to publish, or preparation of the manuscript.”

Reviewer #1:

This manuscript explores how the definition and aggregation of mobile phone–derived mobility data influence epidemic modeling outcomes. While the analysis is technically sound, the overall research question is somewhat limited in scope. The key issue in using mobile phone data for epidemiological modeling lies more in the privacy, ethical, and governance challenges surrounding data access and use. The manuscript would be stronger if it acknowledged and discussed these broader implications more explicitly.

We thank the reviewer for their helpful comments.
NOTE: For clarity and consistency, we revised the notation used throughout the manuscript. We now refer to the displacement-based coupling D as HR (high-resolution), the location-based coupling L as MR (medium-resolution), and the most-visited-location coupling C as LR (low-resolution). This change reflects the level of mobility information retained by each approach and does not alter the underlying definitions or results.

1. The final paragraph of the Introduction reads more like a methodological description than a study rationale. It introduces too many technical details that would be better placed in the Methods section. The Introduction would be clearer if it briefly summarized the motivation and study objective instead of detailing the data aggregation procedures.

We thank the reviewer for this constructive feedback. We have substantially revised the Introduction to address this concern and moved the last part of the original introduction in the Method section. The final two paragraphs now focus on study objectives, and the general methodological approach rather than full methodological details on the aggregation approaches.

2. The Methods section is dense and overlaps with Results. Please consider restructuring into clear subsections, moving formulas and parameter details to Supplementary Information.

We thank the reviewer for this constructive feedback. We have restructured the Methods section into the following subsections for improved clarity: (i) Mobile phone dataset, (ii) From individual trajectories to coupling matrices, (iii) Epidemic metapopulation model, (iv) Comparison between coupling matrices, and (v) Comparison between simulated epidemic outcomes. To reduce formula density, we moved the full model equations to the Supplementary Information, retaining only the formula for the force of infection in the main text. All simulation details related to the model have also been moved to the Supplementary Information. Finally, we simplified the text sections on the comparison of coupling matrices and simulated epidemic outcomes.

3. The study lacks of empirical validation. While the authors acknowledge this, at least some retrospective comparison with known epidemic or cross-validation using synthetic mobility data would strengthen credibility.

We thank the reviewers for raising the important point of empirical grounding. We fully agree that anchoring theoretical findings to real epidemic dynamics is essential. The goal of this study, however, is not to retrospectively fit or reproduce a specific epidemic, but to isolate the role of different mobility coupling definitions under controlled conditions. Synthetic epidemics allow us to disentangle this effect independently of disease-specific uncertainties, interventions, and reporting biases.

To address the reviewers' concern, we have strengthened the manuscript by grounding our results in epidemic invasion patterns observed during the COVID-19 epidemic in the country. We focused on the early epidemic spread and compared the rank of arrival times predicted by the model for each coupling definition with the observed invasion order during the early phase of the epidemic in 2020. As shown in the figure below (Figure 6), the MR and LR coupling definitions reproduce the observed spatial invasion substantially better than the HR displacement-based coupling. We tested two values of R0, R0=1.5 and R0=3, to explore different transmission potentials of COVID-19 in the country, and in both cases we obtained better predictions of the invasion pattern with the MR and LR couplings. For R0=1.5, this is measured by high-rank correlations between predicted and observed arrival times with Spearman's r = 0.65 (MR) and 0.77 (LR), whereas the HR coupling shows weak and non-significant agreement with the observed invasion order (Figure 6a). Consistent results are obtained using Kendall's τ (τ = 0.51 and 0.61 for MR and LR, respectively, versus nonsignificant association for HR). For R0=3, all tests are significant

but correlations remain higher for MR and LR compared to HR (Spearman's r = 0.77 (MR) and 0.77 (LR) compared with r=0.54 for HR (Figure 6b)). The COVID-19 invasion observed in Senegal in 2020 is reported in the map at department level in Figure 6c.

Importantly, these results indicate that coupling definitions preserving origin–destination relationships (MR and LR) better capture the large-scale spatial ordering of epidemic invasion, including rapid long-range transmission and the early involvement of major hubs, compared to model specifications using higher-resolution displacement-based mobility matrices.

A full data-driven analysis of COVID-19, including calibration to epidemiological time series and time-varying mobility, is the focus of a separate dedicated study currently in preparation. We believe separating these contributions allows each paper to address its scientific question with appropriate depth and clarity.

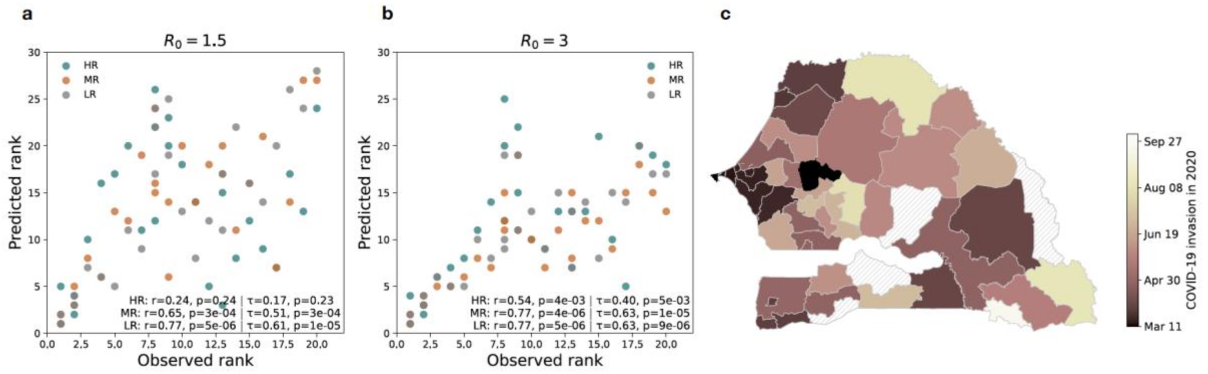

Figure 6. Comparison of observed and predicted COVID-19 invasion during the 2020 first wave. (a) Scatter plot comparing the ranking of observed early COVID-19 arrival times in 2020 with model-predicted arrival times across departments, for the HR, MR, and LR methods. Model predictions correspond to the scenario with R0=1.5 and Dakar as the seed location. **(b)** Same as panel (a), but for the scenario with R0=3 and Dakar as the seed location. Spearman's (r) and Kendall's (τ) correlation coefficients, together with their associated *p*-values, are reported in both panels. **(c)** Spatial distribution of the COVID-19 invasion in Senegal in 2020, shown as department-level arrival times. Dakar and Touba, colored in black, are the first ones to report confirmed COVID-19 cases on March 11, 2020. The maps were generated in Python using administrative boundary shapefiles from the Global Administrative Areas database (GADM), available at https://gadm.org.

Along with Figure 6, we also integrated this text in the Result section:

"To empirically ground our theoretical findings, we analyzed the invasion dynamics of the early COVID-19 pandemic in Senegal in 2020. The epidemic exhibited rapid long-range jumps between major urban hubs rather than gradual geographic diffusion (Figure 6c). The first confirmed cases were reported in Dakar, the capital and location of the international airport where the virus was first introduced. The virus was also detected in Touba, a major religious and economic hub located several hundred kilometers from Dakar. The rapid spread across distant locations highlights the importance of long-range mobility connections in shaping the early spatial spread of the epidemic. We compared the observed invasion order across departments with the arrival times predicted by the three mobility representations. Because early estimates of COVID-19 transmissibility varied widely (approximately R_0 = 1.5–3.5), we evaluated model predictions across this plausible range. The heterogeneous invasion pattern observed in Senegal is well reproduced by the MR and LR models but not by the HR model. For R_0 = 1.5, the predicted and observed arrival rankings are strongly correlated for MR and LR (Spearman rank correlations of r = 0.65 and 0.77, respectively), whereas HR shows weak and non-significant agreement (r = 0.24; Figure 6a). Consistent results are obtained using Kendall's τ (τ = 0.51 and 0.61 for MR and LR respectively, versus non-significant association for HR). At the higher end of the estimated range (R_0 = 3), MR and LR continued to reproduce the observed invasion ordering (Spearman's r = 0.77 for both models), while HR showed weaker agreement (r = 0.54; Figure 6b). The consistent performance of MR and LR across this empirically plausible range of R_0 values indicates that mobility representations capturing where individuals

spend time better reproduce the spatial ordering of epidemic invasion. These findings demonstrate that intermediate-range and long-range mobility coupling, rather than purely local displacement transmission, were the dominant drivers of the heterogeneous spatial spread observed during the early COVID-19 epidemic in Senegal." ."

4. The MRQAP correlation analysis shows matrices are "highly correlated," yet the epidemiological implications of these differences are not fully quantified. Provide additional quantitative metrics to illustrate the magnitude of divergence.

We thank the reviewer for this observation. While the MRQAP analysis demonstrates high statistical correlation between the matrices ($r_{LR,MR} = 1$, $r_{HR,MR} = 0.99$, $r_{HR,LR} = 0.99$), we acknowledge that correlation coefficients alone do not fully capture the epidemiological significance of differences in network structure. To address this concern, we have added a comprehensive network centrality analysis in the Supplementary Information (Figure S4) that quantifies how different aggregation methods affect the structural properties of mobility networks and the identification of epidemiologically critical locations:

1. **Degree Centrality:** Method HR shows systematically lower degree centrality values (median=0.50) compared to MR (median=0.85) and LR (median=0.76). This indicates that the displacement-based approach creates a more fragmented network where each location is directly connected to fewer other locations. As documented in Figure 3g of the main text, 37% of links present in MR are absent in HR, predominantly long-range connections. This network fragmentation has direct epidemiological consequences: locations in HR have fewer pathways for disease introduction, explaining the delayed arrival times and localized spread patterns observed in our epidemic simulations (Figure 4).
2. **Closeness Centrality:** Method HR shows systematically lower closeness centrality values (median=0.66) compared to MR (median=0.87) and LR (median=0.80), indicating that locations are structurally farther apart in the network topology, more hops are required to reach distant locations. This directly translates to the delayed epidemic arrival times observed in our simulations. While L and LR enable rapid long-range transmission through direct connections (e.g., Dakar to Touba in one hop), HR fragments these trips into multiple short-distance hops, increasing the effective network distance. This structural difference quantitatively explains why the maximum geographical distance achieved at $t_{5\%}$ is much smaller in HR (Figure 4c) and why HR produces unrealistic localized epidemic patterns that fail to match observed spatial invasion dynamics of COVID-19 diffusion.
3. **Betweenness Centrality:** While the median betweenness centrality is comparable across all three methods (~0.0004), HR exhibits higher values for few nodes compared to MR and LR. This pattern reveals a critical structure in HR: the fragmented network with reduced direct connections (low degree) forces epidemic pathways through a limited set of intermediate nodes, creating artificial bottlenecks. These nodes accumulate high betweenness centrality. However, those nodes are not epidemiologically important hubs in reality, but because the network topology leaves few alternative paths, they function as key hubs. This explains the radial diffusion patterns observed in HR, the epidemic is constrained to spread through these artificial bottlenecks rather than propagating through the direct long-range connections captured by MR and LR.
4. **PageRank:** While the median PageRank is similar across all methods (~0.0025), D shows a smaller confidence interval compared to MR and LR. The narrow distribution in HR indicates a failure to capture the hierarchical organization of mobility networks: HR cannot differentiate between major epidemic hubs (such as Dakar and Touba) and peripheral rural locations. All locations appear similar. In contrast, the wide PageRank distributions in MR and LR reflect realistic network heterogeneity with clear hierarchical structure: a few major hubs with very high PageRank (capital cities, major), and many peripheral locations (likely to be rural areas) with low PageRank.

We updated the Supplementary Information as follows:

"To complement the MRQAP correlation analysis and further quantify the structural differences between coupling networks constructed with methods HR, MR, and LR, we computed four standard network centrality metrics for all 394 municipalities (Figure S4). These metrics provide quantitative measures of node importance and network topology that have direct epidemiological interpretation: degree centrality identifies the number of direct pathways for disease introduction; closeness centrality measures how quickly infections can spread from a location to all others; betweenness centrality identifies critical bridge locations whose removal would fragment transmission pathways; and PageRank captures the hierarchical importance of locations considering both direct connections and connections to other important nodes. The

centrality analysis reveals the three aggregation methods produce networks with fundamentally different structural properties. Method HR shows lower degree (median=0.50) and closeness centrality (median=0.66) compared to MR (degree median=0.85, closeness median=0.87) and LR (degree median=0.76, closeness median=0.80), indicating a fragmented network with longer effective distances. While median betweenness centrality is comparable across methods (~0.0004), D exhibits extreme values (maximum ~0.0030) for certain nodes—more than double the maximum observed in MR and LR (maximum ~0.0015)—indicating artificial bottlenecks. PageRank shows similar medians across all methods (~0.0025), but HR displays a dramatically narrower distribution (IQR: 0.0024-0.0026) compared to MR (IQR: 0.0023-0.0027) and LR (IQR: 0.0023-0.0027), indicating failure to capture network hierarchy. In contrast, methods MR and LR show similar centrality distributions with higher degree and closeness values, moderate betweenness without extreme outliers, and heterogeneous PageRank distributions reflecting realistic hierarchical structure.

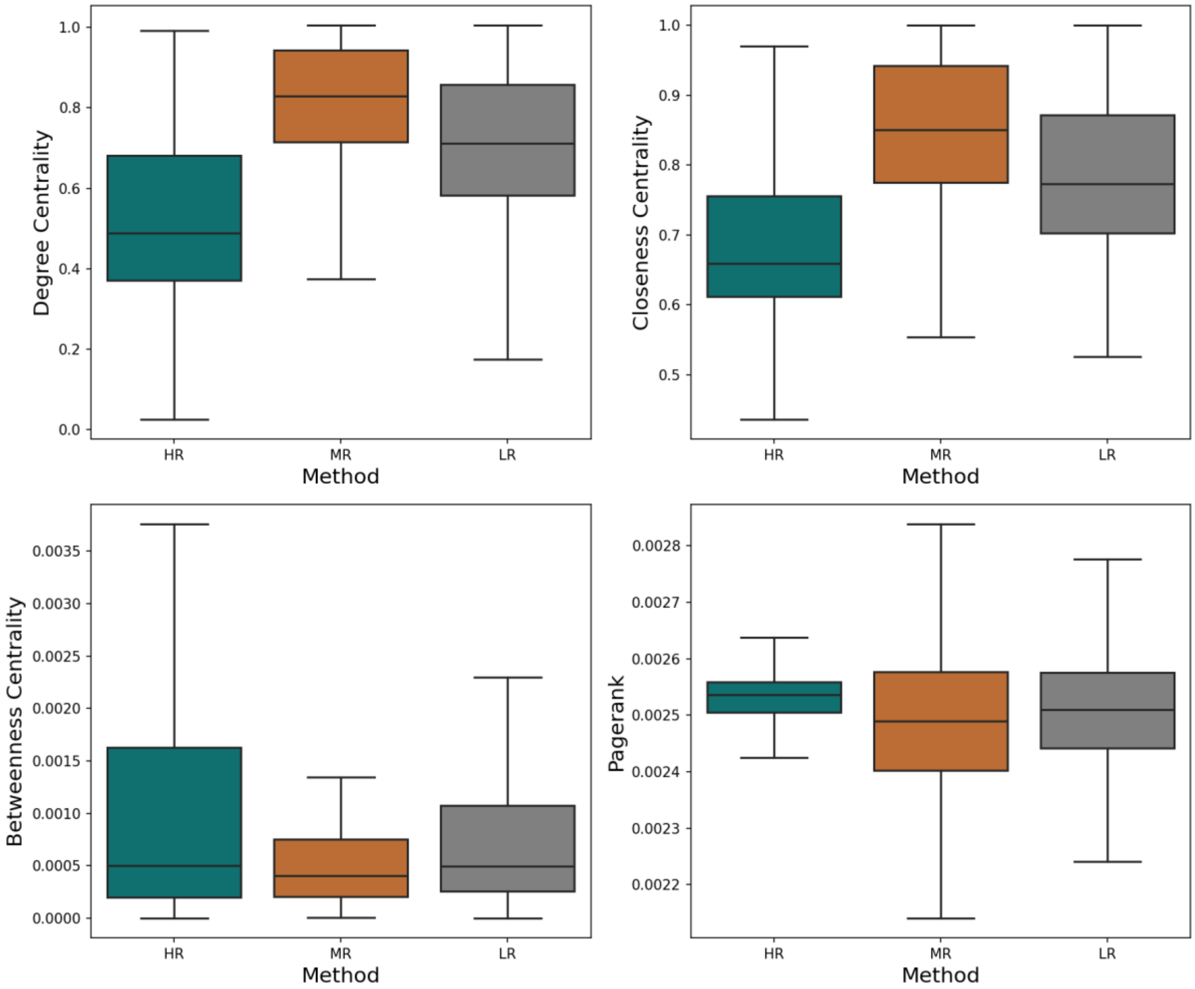

*"**Figure S4.** Network centrality metrics comparison across HR, MR, LR. Box plots showing the network centrality metrics across all 394 municipalities in the daily average coupling networks LR for each month in 2013. a) Degree centrality. b) Closeness centrality. c) Betweenness centrality. d) PageRank. Box plots indicate median and 95% CI."*

5. Figures 2–6 are information-dense and sometimes repetitive. The Results would benefit from a more streamlined structure.

We thank the reviewer for this suggestion. We have streamlined Figures 2–6 to reduce redundancy and improve structure. Specifically, we have made the following revisions:

- **Figure 2:** Removed panels c and d. Panel c, which showed distance between networks, was removed since all key distance-related analyses are already shown in Figure 3. Panel d was removed as the plot displayed information already captured in the maps in the figure.
- **Figure 3:** Removed panels a and b, as this information was already presented in Figure 2. In panel g, we also removed the matrix showing coupling probabilities, retaining only the distance-focused analysis which was the key result.
- **Figure 4:** Removed panel c, which displayed the time required for the infection to reach 5% of municipalities. This panel was overly complicated and did not provide additional insights beyond the results already presented.

We moved **Figure 6** (sensitivity analysis) to the Supplementary Materials.

6. The conclusion that "tracking individual displacements is less epidemiologically informative than focusing on most visited locations" is compelling but should be contextualized. Are these findings generalizable beyond Senegal, or dependent on network density and mobility scale? The statement that "privacy can be enhanced without losing epidemiological value" is conceptually strong but speculative; it should be qualified more carefully.

We appreciate the reviewer's comment. Mobility patterns do indeed differ between developing and developed contexts, with residents in low-income settings typically making fewer, shorter trips per day (mean ≈5 km), whereas those in high-income settings tend to make more frequent and longer ones (mean ≈10–15 km) [link]. That said, a substantial body of literature has established that human mobility is well described by parsimonious generative models, gravity and radiation models, across both high-income and low- and middle-income country (LMIC) settings (Barbosa al., Phys. Rep., 2018). Notably, Balcan et al. (PNAS, 2009) demonstrated that commuting flows can be reliably fitted at a global scale, suggesting that the fundamental structure of daily human circulation between home and regularly visited locations is a general feature of mobility that transcends development context.

The spatial scale of analysis may, however, affect the generalizability of our quantitative results. Using municipalities as spatial units allows us to capture substantial heterogeneity across locations; coarser aggregations would smooth over these differences and likely attenuate the observed magnitude of discrepancies between methods.

Importantly, the distinction between aggregation approaches is not primarily driven by network density: in our data, density remains relatively high across all methods (MR: 90%, LR: 84%, HR: 70%). Reducing the number of individual trajectories or trips would lower density uniformly across networks, but differences in long-range connectivity would persist. In high-resolution networks (HR), long-range connections are inherently underrepresented because only consecutive displacements are captured, whereas medium- and low-resolution approaches (MR, LR) retain these links through home–destination relationships. This structural difference, not overall density, explains why HR networks are less epidemiologically informative and represents a fundamental limitation of this method that is independent of setting: by chaining any two sequentially visited locations, it severs the epidemiologically meaningful connection between where people reside and where they spend significant time, thereby misrepresenting the coupling forces that drive epidemic invasion regardless of the characteristic trip distances or network properties of a given country.

To make this point explicit in the paper, we have added the following to the Discussion section:

"*While this study focuses on Senegal, we are aware that mobility patterns differ across socioeconomic contexts, with implications for how aggregation methods may behave. In low-income settings, daily mobility often consists of fewer, shorter trips (mean ≈5 km), whereas in high-income settings mobility typically exhibits more frequent and longer commuting trips (mean ≈10–15 km). That said, a substantial body of literature has established that human mobility is well described by parsimonious generative models, gravity and radiation models, across both high-income and low- and middle-income country* (LMIC) settings[16]. Notably, Balcan et al.[14] *demonstrated that commuting flows can be reliably fitted at a global scale, suggesting that the fundamental structure of daily human circulation between home and regularly visited locations is a general feature of mobility that transcends development contexts. These structural differences, moreover, do not alter our methodological conclusions, because the failure of HR arises from trip fragmentation rather than from any property of trip length itself: by chaining sequentially visited locations, HR severs the epidemiologically meaningful connection between where people reside and where they spend significant time, misrepresenting the coupling forces that drive epidemic invasion regardless of*

characteristic trip distances. While the specific magnitude of differences between methods may vary with local mobility patterns, infrastructure, and socioeconomic conditions, the core distinction between origin-destination preservation and trip fragmentation should generalize across settings. The spatial scale of analysis may nonetheless affect the generalizability of our quantitative results, as using municipalities as spatial units allows us to capture substantial heterogeneity across locations, whereas coarser aggregations would smooth over these differences and likely attenuate the observed magnitude of discrepancies between methods. Indeed, we expect differences between methods to diminish at coarser scales, where averaging obscures the heterogeneities that distinguish approaches, but empirical evaluation across scales remains an important direction for future work. Validation in diverse geographic and economic contexts would further strengthen the universality of these findings."

Finally, we agree that our previous statement regarding privacy was speculative. While our results show that low resolution data (LR) can retain epidemiological value, we did not formally quantify reductions in privacy risks. We have therefore revised the text to provide a more accurate formulation in Discussion section:

"In the post-COVID-19 era, direct access to individual trajectories has become increasingly restricted, while aggregated mobility indicators are more widely available. Our findings challenge the assumption that more granular data yields better epidemic predictions. Instead, aggregated indicators describing time spent at major activity locations appear sufficient for predictive spatial models. This insight supports the development of privacy-preserving data-sharing frameworks that reduce the sensitivity of mobility data while preserving their epidemiological value."

7. Please clarify whether R_0 = 1.1–3 corresponds to realistic ranges for the diseases simulated.

We selected values of the basic reproduction number to represent distinct and epidemiologically realistic transmissibility regimes, rather than targeting a specific disease. These values were chosen to represent a range of realistic epidemic contexts. Specifically, R_0 = 1.1 corresponds to controlled epidemic conditions, as observed for SARS-CoV-2 during periods of effective non-pharmaceutical interventions. R_0 = 1.5 reflects moderate transmissibility, consistent with typical estimates reported for seasonal influenza and the 2009 H1N1 pandemic. Finally, R_0 = 3 represents high-transmissibility scenarios, compatible with estimates reported for SARS-CoV-2 in susceptible populations and for Ebola virus disease during intense community transmission. This range allows us to assess the robustness of our conclusions across epidemiologically plausible epidemic scenarios.

To clarify this upfront, we have added a corresponding explanation in the Abstract as follows:

"We develop a metapopulation epidemic model that explicitly accounts for transmission from residents, visitors, and returning travelers, then simulate diseases with varying transmission characteristics, representing controlled scenarios with effective non-pharmaceutical intervention, seasonal influenza, and high-transmissibility diseases like early-stage COVID-19 and Ebola."

And in the Introduction:

"We then quantify how these differences affect simulated epidemic dynamics using a metapopulation model under three transmissibility regimes: low transmission (R_0 = 1.1), representing controlled epidemic conditions such as SARS-CoV-2 under strong interventions; moderate transmission (R_0 = 1.5), consistent with seasonal influenza–like dynamics; and high transmission (R_0 = 3.0), representing highly transmissible pathogens such as early SARS-CoV-2 or Ebola during intense community transmission. ."

And in the Method section:

"We explored three epidemic scenarios corresponding to different transmission intensities: (i) low transmissibility ($\square_0 = 1.1$), representing controlled epidemic conditions, as observed for SARS-CoV-2 during periods of effective non-pharmaceutical interventions; (ii) medium transmissibility ($\square_0 = 1.5$), representing moderate transmissibility, consistent with typical estimates reported for seasonal influenza and the 2009 H1N1 pandemic; (iii) high transmissibility ($\square_0 = 3$), compatible with estimates reported for SARS-CoV-2 in susceptible populations and for Ebola virus disease during intense community transmission"

Reviewer #2:

Based on mobile phone data encompassing 9,569,425 users in Senegal from January to December 2013, the authors quantified corresponding coupling matrices to evaluate the influence of mobility definitions on disease spread. This study presents interesting investigation. Intuitively, one might assume that higher spatial resolution would enhance the predictive power of epidemic models. However, the results of this study suggest the opposite, which is somewhat counterintuitive. In fact, the required resolution depends heavily on the specific scientific question being addressed and the perspective from which it is examined.

We thank the reviewer for their helpful comments.

NOTE: For clarity and consistency, we revised the notation used throughout the manuscript. We now refer to the displacement-based coupling *D* as HR (high-resolution), the location-based coupling *L* as MR (medium-resolution), and the most-visited-location coupling *C* as LR (low-resolution). This change reflects the level of mobility information retained by each approach and does not alter the underlying definitions or results.

Additionally, the following issues warrant further clarification or elaboration by the authors:
1. Definition of Coupling Force: Could the authors provide a detailed explanation of what "coupling" refers to in this context? Why are the number of calls or text messages used as proxies for coupling? The direct relationship between call/SMS frequency and disease transmission remains unclear.

We thank the reviewer for this important clarification. To clarify notation, we renamed D as HR (high-resolution), L as MR (medium-resolution), and C as LR (low-resolution). In epidemiological metapopulation models, "coupling" refers to the connectivity between spatial locations that drives pathogen transmission. The coupling force between two locations quantifies the probability for disease spread from one location to another through human movement. We use the number of calls or text messages as proxies for coupling because each mobile phone communication event (call or SMS) provides the geo-location of the user at cell tower level and the moment of the activity. By collecting individual daily activities and aggregating at municipality level, we can track:

- **Individual daily displacements**: Sequential connections to different municipalities to reveal movement patterns between locations.
- **Home location**: Identified as the location with the highest number of nighttime activities by user
- **Visited locations and time spent at these locations**: Identified as the location with some number of activities by user during a 24h time period. Most visited locations are the locations with the highest number of activities during a 24h time period.

The relationship between call/SMS frequency and coupling (alias disease transmission) depends on the mobility aggregation approach:

1. **For high-resolution coupling (HR)**: The coupling quantifies the potential for disease spread through the number of physical displacements between two locations. Each displacement (two consecutive phone activities) represents an opportunity for an infected individual to travel from one location to another, potentially introducing infection in a new visited place.
2. **For medium-resolution- and low-resolution coupling (MR and LR)**: The number of mobile phone activities serves as a proxy for time spent in a location. Individuals who spend more time in a location (indicated by more frequent activities there) have greater opportunity for transmitting infection there. The coupling thus quantifies disease transmission from home locations to each visited (MR) or most visited (LR) place.

To clarify this concept, we have added the following explanation to the Methods section: **From individual trajectories to coupling matrices*:***

"Coupling matrices quantifies the connectivity strength between spatial locations that enables pathogen transmission through human movement. Each element p_{ij} represents the monthly averaged probability that an individual residing in i travels to location j in a given day. Consequently, p_{ij} captures the opportunity for exposure of individuals residing in location i to individuals present in location j and potential transmission in j. Mobile phone communication events (calls and SMS) provide geo-location data at cell tower resolution. Each event marks the user's physical presence at a specific time and place, allowing us to track individual daily consecutive displacements and time spent in different locations as a proxy of the number of activities in a location. By aggregating individual mobility patterns across all users, we extracted three coupling matrices at different resolution levels, computing 12 daily average matrices for each month in 2013 at the municipality level for each method."

2. Magnitude Discrepancy in Matrix D: What accounts for the order-of-magnitude difference between the elements of matrix D and those of the other two matrices?

We thank the Reviewer for this observation. The central challenge in epidemic modeling is: (1) identifying which locations are epidemiologically relevant for disease transmission (where transmission occurs) and (2) determining how these locations are coupled through human movement to enable spatial spread. Different aggregation methods answer these two questions differently. The displacement-based approach HR treats every location where a phone activity occurs as a potential transmission site, with coupling defined by consecutive movements. In contrast, the location-based matrix MR and commuting-based matrix LR anchor spatial transmission between home location and the locations visited by the user, through coupling defined by home-to-activity flows. These conceptual differences result in different network structures generated by the HR aggregation versus the home-centric aggregation in MR and LR and are reflected in the observed magnitude discrepancies between matrices.

Matrix HR is constructed from consecutive displacements between mobile phone activities, without using home location. This produces two systematic effects:

1. Overestimation of probability of staying in the same location (p_{ii}).
   Every time a person remains in the same location between two consecutive phone events, this is counted 1 as a displacement from i → i. non-movement events are frequent and accumulate on the diagonal, leading to very large p_{ii} values. This results in an underestimation of the outgoing probability (1- p_{ii}).
2. Underestimation of coupling probabilities (p_{ij}).
   True trips (e.g., home → work) may be fragmented into multiple short steps (home → street → bus stop → workplace). As a result, the weight is spread across several short-range links instead of concentrated on a single i (home)→ j (work) link, reducing the connections of the real origin-destination.

In contrast, matrices MR and LR explicitly connect each individual's home location to their visited locations (MR) or daily most visited locations (LR). Their diagonal terms represent only users who never leave home, and each activity outside the home produces a direct i → j link without intermediate steps. This results in fewer but stronger long-range connections, and therefore larger coupling probabilities. These structural differences explain the observed order-of-magnitude discrepancy across matrices and reflect the distinct mobility assumptions each method captures.

To clarify this aspect, we added in the Results in "Location and commuting-based matrices better represent epidemic-relevant mobility" session:

"Because HR is constructed from consecutive mobile phone events, periods in which individuals remain in the same location generate self-loops, substantially reducing the probability of moving. Trips are partitioned into multiple short consecutive segments, causing the corresponding movement probability to be distributed across several intermediate links rather than concentrated on a single origin–destination connection, as occurs in MR and LR. Because of these two effects, coupling probabilities in HR are smaller and diagonal elements in the HR matrices are larger, whereas non-diagonal elements are smaller, producing the observed differences between HR and the matrices MR and LR."

3a. Unexpected Performance of High-Resolution Model D: Why does matrix D, despite its higher resolution, yield the least realistic epidemic simulations? Could this be related to the structure of the transmission model or the specific transmission route of the pathogen (e.g., aerosol transmission)?

We thank the reviewer for this comment that touches on several important aspects of spatially-explicit epidemic modeling.

The poorer performance of matrix HR (formerly D) is not due to the resolution per se, but rather because it captures mobility patterns that are epidemiologically less relevant for spatial disease spread in a metapopulation framework. Matrix HR records all consecutive displacements, including many short-range movements that do not meaningfully contribute to between-location transmission for infectious diseases. As shown in our results and explained in detail in the answer to the previous comment, HR overestimates the probability of not moving (p_{ii}) and underestimates long-range connectivity, failing to capture the critical mobility patterns that drive spatial invasion for emerging infections.

Our findings are robust across pathogens with different transmission characteristics (R_0 = 1.1 to 3.0). For diseases transmitted through close human contact, whether respiratory or otherwise, what matters epidemiologically is sustained co-location in the same place, not the path taken to get there. An individual spending 8 hours at work can transmit infection to colleagues regardless of whether they took a direct route or made several stops along the way. Matrix HR records all those intermediate movements, adding detail about pathways that does not improve prediction of where sustained transmission occurs.

To make clearer why matrix HR, despite its higher resolution, yields the least realistic epidemic simulations, we added in the Discussion section:

"The poor performance of method HR stems from a mismatch between data granularity and epidemiological relevance. Rather than resolution itself being problematic, D captures mobility patterns that are epidemiologically less meaningful for spatial transmission. By fragmenting continuous trips into multiple artificial steps between consecutive calls, HR fails to identify true origin-destination pairs, the actual locations where sustained contact enables transmission. This fragmentation has three critical consequences: (i) it substantially underestimates long-distance connectivity by breaking journeys into short hops, (ii) it assigns equal weight to each displacement regardless of time spent, failing to distinguish epidemiologically important locations (homes, workplaces, schools) from places individuals merely pass through."

3b. Since D captures path-based mobility, would an edge-based network modeling approach be more suitable for it?

Matrix HR captures path-based mobility (journeys between consecutive locations), while MR and LR are more node-centered (presence at destinations) and connect directly the home location with the visited locations, ignoring the path. However, the choice of modeling framework (edge-based versus node-based networks) does not resolve the epidemiological issue. Even with an edge-based network approach, matrix HR would still produce spatial transmission along movement paths (during transit between locations) which is epidemiologically unrealistic for most diseases at regional scales.

However, despite HR being epidemiologically less appropriate, displacement-based approaches are widely used in metapopulation epidemic modeling in the literature and as aggregation approaches from data providers to extract origin-destination matrices. This is why we wanted to demonstrate that in the context of spatial epidemic spread at regional scales, HR is not appropriate for capturing the heterogeneity in long-range geographical diffusion, even though it is commonly employed. For metapopulation models focused on spatial invasion, where transmission occurs during sustained presence at destination nodes (homes, workplaces, schools), methods MR and LR are more suitable. This study gives the opportunity to assess that not all aggregated location data that data providers share is suitable for metapopulation modeling and network transmission modeling more broadly.

Matrix HR would instead be more appropriate for different research questions, including migration studies where changing home locations over time is the focus, and therefore the connections along the set of consecutive home locations observed during migration become relevant.
To make clearer the generalizability of results across models, we added in the Discussion section:

"Finally, to test the robustness of our findings, we performed a sensitivity analysis by introducing time-corrected versions of the three coupling matrices: HR', MR', and LR'. Those matrices are designed to reduce the bias introduced by heterogeneity in users' calling activity. HR' redefines displacement weight as the time elapsed between consecutive moves; MR' replaces call counts with the effective time spent in each visited location; and LR' applies the same correction to the most visited location matrix, ensuring coupling strengths reflect time allocation rather than call frequency (see Supplementary Information for full detail).

While MR' and LR' showed minimal differences from their original counterparts in terms of epidemic outcomes, HR ' demonstrated substantial improvements over HR, producing spatial diffusion patterns more similar to MR and LR (Figure S15-19). However, HR' still exhibited limitations, with invasion distance distributions remaining higher than in MR and LR due to approximately 40% of epidemiologically relevant links present in MR and LR being absent in HR' (Figure S15). This suggests that even refined displacement-based approaches may not adequately capture epidemiological couplings between locations for diseases where daily mobility patterns drive transmission."

3c. For many respiratory infectious diseases with complex transmission pathways, how significant is the role of such coupling forces?

We acknowledge that many factors contribute to epidemic dynamics including environmental conditions, population susceptibility, pathogen transmissibility, built environment characteristics. However, coupling forces expressed in terms of human mobility represent the mechanism for the spatial transmission of directly transmitted infectious diseases, offering novel nonlocal opportunities for exposure and transmission.] A substantial body of literature on influenza, COVID-19 influenza, SARS, and other respiratory pathogens has demonstrated that connectivity and regional mixing patterns are key drivers of the geographical dynamics of importation cases into new locations. (Charu et al. PLOS Comp Bio, 2017, Pullano et al., JMIR Public Health and Surveillance, 2025, Colizza, BMC Medicine, 2007, Findlater et al., Trends in Parasitology, 2018). In the context of Senegal, invasion dynamics of early COVID-19 observed in 2020 also exhibited jumps between epidemic hubs location and we have shown how those can be explained by coupling forces between Dakar and main cities like Touba and Thiès.

Our study specifically focuses on optimizing the coupling network structure needed to capture these spatial invasion dynamics. While other factors certainly modulate transmission intensity once disease arrives in a location, the coupling forces determine where and when disease will arrive, a fundamental question for spatial epidemic prediction and preparedness.

To make clearer the generalizability of results across models, we added in the Introduction section:

"While many factors may contribute to epidemic dynamics -including environmental conditions, population susceptibility, pathogen transmissibility and the built environment - human mobility represents the primary mechanism explaining the spatial invasion patterns observed across diseases including influenza[4-6], COVID-19[7-9], and SARS[10-12]."

4. Temporal Resolution of Coupling Matrices: The authors used monthly coupling matrices. Why was a daily resolution not adopted, especially since the SEIR model operates on a daily time step? Would a daily matrix provide better temporal alignment with the disease dynamics?

We thank the reviewer for raising this important point regarding the temporal resolution of the coupling matrices. In the simulation, we used 12 coupling matrices representing the daily average mobility patterns for each month in 2013. This approach is well established in literature (Ruktanonchai et al. PLOS Computation Biology, 2016, Wesolowski et al., Science, 2013) and ensures that the mobility inputs remain aligned with the daily dynamics of disease transmission while smoothing out day-to-day fluctuations, such as weekly cycles or holiday-specific anomalies, since our goal was to capture general patterns rather than events specific to a particular period. Using daily averages instead of daily matrices also helped avoid technical issues related to data noise including cell towers malfunctioning in specific days. To make this distinction clearer in the manuscript, we have referred to these matrices as "Monthly-aggregated, daily-normalized coupling matrices" rather than simply "monthly matrices." In our previous work (Pullano et al., *JMIR Public Health and Surveillance*, 2025), we also demonstrated that spatial invasion patterns remain stable when coupling matrices are held constant over long periods versus updated in real-time, indicating that real-time temporal fluctuations in mobility are not the primary driver of invasion and static representation of the mobility are sufficient to capture the invasion path.

Reviewer #3:

The paper addresses an important and timely question, namely how to incorporate mobility data into epidemic models when mobile phone data are available. The authors explore three implementations with varying levels of granularity and privacy, embed them in a metapopulation infectious disease model, and assess the resulting differences. The topic is highly relevant for the epidemic modeling community, and the methodological foundations are generally sound. However, in its current form, the manuscript is challenging to follow. I encourage the authors to substantially revise the presentation of the results. Below I outline several major points that could help strengthen the paper.

We thank the reviewer for their helpful comments.

NOTE: For clarity and consistency, we revised the notation used throughout the manuscript. We now refer to the displacement-based coupling *D* as HR (high-resolution), the location-based coupling *L* as MR (medium-resolution), and the most-visited-location coupling *C* as LR (low-resolution). This change reflects the level of mobility information retained by each approach and does not alter the underlying definitions or results.

1) Even if full calibration with real data is beyond the scope of the study, the central claim that L and C are sufficient to reproduce realistic patterns of diffusion would benefit from a brief comparison between simulated epidemic patterns and real observations for Senegal. This would ground the main conclusions and increase the impact of the work.

We thank the reviewers for raising the important point of empirical grounding. We fully agree that anchoring theoretical findings to real epidemic dynamics is essential. The goal of this study, however, is not to retrospectively fit or reproduce a specific epidemic, but to isolate the role of different mobility coupling definitions under controlled conditions. Synthetic epidemics allow us to disentangle this effect independently of disease-specific uncertainties, interventions, and reporting biases.

To address the reviewers' concern, we have strengthened the manuscript by grounding our results in epidemic invasion patterns observed during the COVID-19 epidemic in the country. We focused on the early epidemic spread and compared the rank of arrival times predicted by the model for each coupling definition with the observed invasion order during the early phase of the epidemic in 2020. As shown in the figure below (Figure 6), the MR and LR coupling definitions reproduce the observed spatial invasion substantially better than the HR displacement-based coupling. We tested two values of R0, R0=1.5 and R0=3, to explore different transmission potentials of COVID-19 in the country, and in both cases, we obtained better predictions of the invasion pattern with the MR and LR couplings. For R0=1.5, this is measured by high rank correlations between predicted and observed arrival times with Spearman's r = 0.65 (MR) and 0.77 (LR), whereas the HR coupling shows weak and non-significant agreement with the observed invasion order (Figure 6a). Consistent results are obtained using Kendall's τ (τ = 0.51 and 0.61 for MR and LR, respectively, versus non-significant association for HR). For R0=3, all tests are significant but correlations remain higher for MR and LR compared to HR (Spearman's r = 0.77 (MR) and 0.77 (LR) compared with r=0.54 for HR (Figure 6b)). The COVID-19 invasion observed in Senegal in 2020 is reported in the map at department level in Figure 6c.

Importantly, these results indicate that coupling definitions preserving origin–destination relationships (MR and LR) better capture the large-scale spatial ordering of epidemic invasion, including rapid long-range transmission and the early involvement of major hubs, compared to model specifications using higher-resolution displacement-based mobility matrices.

A full data-driven analysis of COVID-19, including calibration to epidemiological time series and time-varying mobility, is the focus of a separate dedicated study currently in preparation. We believe separating these contributions allows each paper to address its scientific question with appropriate depth and clarity.

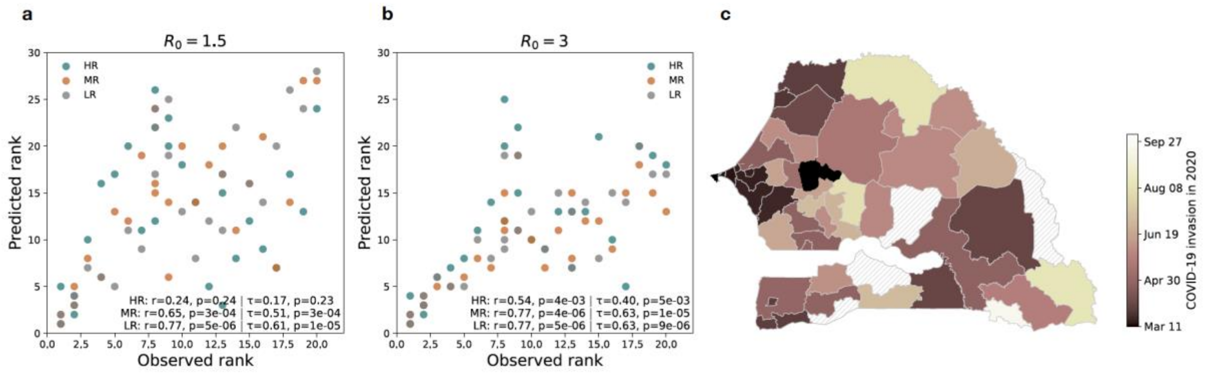

Figure 6. Comparison of observed and predicted COVID-19 invasion during the 2020 first wave. (a) Scatter plot comparing the ranking of observed early COVID-19 arrival times in 2020 with model-predicted arrival times across departments, for the HR, MR, and LR methods. Model predictions correspond to the scenario with R0=1.5 and Dakar as the seed location. **(b)** Same as panel (a), but for the scenario with R0=3 and Dakar as the seed location. Spearman's (r) and Kendall's (τ) correlation coefficients, together with their associated *p*-values, are reported in both panels. **(c)** Spatial distribution of the COVID-19 invasion in Senegal in 2020, shown as department-level arrival times. Dakar and Touba, colored in black, are the first ones to report confirmed COVID-19 cases on March 11, 2020. The maps were generated in Python using administrative boundary shapefiles from the Global Administrative Areas database (GADM), available at https://gadm.org.

Along with Figure 6, we also integrated this text in the Result section:

"In the context of Senegal, we analyzed the invasion dynamics of early COVID-19 observed in 2020, which exhibited jumps between epidemic hub locations (Figure 6c). The epidemic started in Dakar, the capital and location of the international airport, where the virus was first introduced. Touba, despite being distant from Dakar, reported confirmed cases on the same day as the capital. This pattern of simultaneous or close in time emergence in distant locations, rather than gradual geographic diffusion, represents a key feature that our models needed to capture. The heterogeneous COVID-19 invasion pattern shown in Figure 6c was robustly reproduced by the MR and LR models but not by the HR model. Our comparison of predicted and observed times of arrival during the early phase of the COVID-19 epidemic at the department level demonstrates that MR and LR more accurately captured the spatial spread dynamics. For R_0 = 1.5, which falls within the lower range of early pandemic estimates (1.5-3.5), we found Spearman rank correlations of r = 0.65 (MR) and 0.77 (LR), whereas the HR coupling shows weak (r = 0.24) and non-significant agreement with the observed invasion order (Figure 6a). We obtained consistent results using Kendall's τ, with τ = 0.51 and 0.61 for MR and LR respectively, versus non-significant association for HR. At the higher end of the estimated range, R_0 = 3, we found similarly strong performance for MR and LR models, with Spearman's r = 0.77 for both, compared with r = 0.54 for HR (Figure 6b). The robust agreement across this range of R_0 values validates our findings under realistic transmission scenarios for early COVID-19. These findings demonstrate that intermediate-range and long-range mobility coupling, rather than purely local transmission, were the dominant drivers of the heterogeneous spatial invasion pattern we observed in Senegal's early COVID-19 epidemic."

2) A more consolidated summary in the discussion section of the measures used to compare the three mobility matrices, as well as of the key findings, would improve readability. Some of these elements are already present in the results section, and moving them into a single coherent discussion would help readers approach that section independently.

We thank the reviewer for this suggestion. We reviewed the Discussion section and fully rewrote it to include brief summaries of both methods and findings in the first two paragraphs.

3) The treatment of temporal variation in mobility is not fully clear. The authors state that they extract twelve monthly mobility matrices for each implementation. A more detailed discussion of the temporal patterns of these matrices would be valuable and might provide additional epidemiological insights. In addition, it is not explained how the monthly matrices are used in the comparisons. For example, are L

and C compared month by month, or are values aggregated? For Figure 2a the methodology is explicit, but the same level of clarity is needed throughout. The current description of the hierarchical clustering does not fully resolve this point.

We thank the reviewer for this important observation.

We have now clarified how the figures refer to the monthly-aggregated, daily-normalized coupling matrices and not an aggregated static version. The updated labels in Figure 2 and 3 now state:

*"**Figure 2. Coupling forces. a)** Coupling probability distribution in HR, MR, and LR. Distribution includes matrices for all months in 2013. **b)** Outgoing probability (1 - p_{ii}) distribution in HR, MR, and LR. Distribution includes matrices for all months in 2013 **c)** Coupling probability stratified by municipality type. Box plots show the 95% reference range across all months for links connecting Urban-Urban (UU), Urban-Rural (UR), Rural-Urban (RU), and Rural-Rural (RR) municipalities. **d)** Spatial distribution of outgoing probability. Maps show the outgoing probability for each municipality in HR, MR, and LR for January 2013."*

*"**Figure 3. Differences between HR, MR and LR. a, b)** Coupling probability distributions for common links in all 12 matrices as a function of relative variation threshold (x). Subsets include only links where the relative variation Δ between methods exceeds x. Lines show coupling probabilities for HR and MR (a) or MR and LR (b); dashed lines indicate median geographical distance of links for each subset. **c, d)** Characteristics of link subsets as a function of relative variation threshold (x) for all 12 matrices. Solid line: percentage of links in each subset. Dotted line: percentage of locations (nodes) represented in each subset. Stacked bars: distribution of links by municipality type (UU: Urban-Urban, UR: Urban-Rural, RU: Rural-Urban, RR: Rural-Rural). **e-f)** Matrices showing unique links unique to each method for all 12 matrices. Yellow matrix: percentage of links present in the column method but absent in the row method. Violet matrix: among unique links, percentage connecting municipalities at distances greater than the median."*

We also added a comprehensive supplementary figure (Figure S5) that explicitly shows the month-by-month analysis of all three mobility matrices (HR, MR, LR) across the entire 12-month period. As Figure S5 shows, network metrics remained stable throughout the year, demonstrating that our findings are not driven by seasonal variations. Link density varied minimally across months, with HR consistently showing the lowest connectivity (43-48%), MR the highest (68-73%), and LR intermediate values (60-65%), maintaining the hierarchical ordering HR < MR < LR across all months. The degree distributions and self-connection probability (p_ii) showed consistent patterns month-to-month, with MR and LR maintaining stable p_ii values (0.6-0.8) while HR exhibited slightly higher and more variable values (0.75-0.95). Most importantly, the order-of-magnitude differences in inter-connection probability (p_ij) persisted across all months, with HR consistently showing lower coupling probabilities (median ~10^-6) compared to MR and LR (median ~10^-4 to 10^-3), confirming that the differences between methods represent fundamental characteristics of each aggregation approach rather than temporal fluctuations.

The Supplementary Material has now been updated as follows:

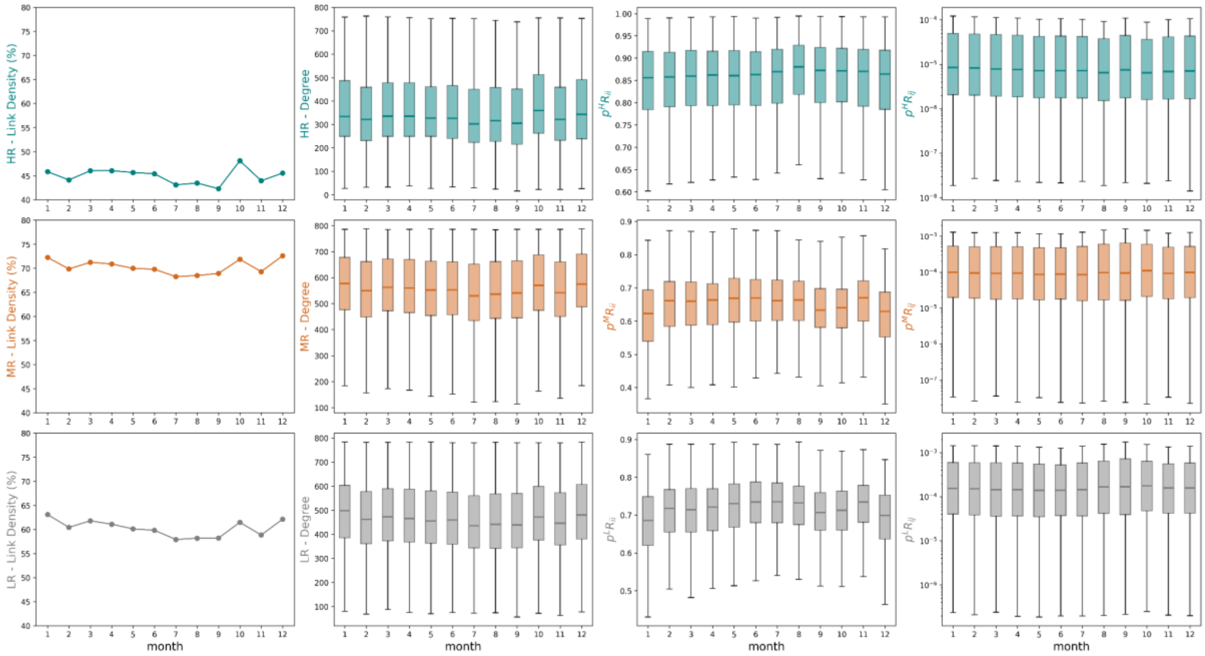

"Figure S5: Monthly-aggregated, daily-normalized coupling matrices for three mobility aggregation methods extracted from mobile phone data in Senegal, January-December 2013. Each row corresponds to one aggregation method (top: HR, middle: MR, bottom: LR). Columns display four network properties: (Left) Link density (%), calculated as the percentage of realized connections among all possible municipality pairs, with each point representing one month; (Second column) Degree distribution showing the number of connections per municipality, displayed as box plots (whiskers: 5th-95th percentile, box: 25th-75th percentile, line: median) for each month; (Third column) Self-connection probability (p_ii) distribution, representing the probability that individuals remain in their home municipality; (Right) Inter-connection probability (p_ij) distribution on a logarithmic scale, representing coupling strength between different municipalities."

"As Figure S5 shows, network metrics remained stable throughout the year, demonstrating that our findings are not driven by seasonal variations. Link density varied minimally across months, with HR consistently showing the lowest connectivity (43-48%), MR the highest (68-73%), and LR intermediate values (60-65%), maintaining the hierarchical ordering HR < LR < MR across all months. The degree distributions and self-connection probability showed consistent patterns month-to-month, with MR and LR maintaining stable values (0.6-0.8) while D exhibited slightly higher and more variable values (0.75-0.95). Most importantly, the order-of-magnitude differences in inter-connection probability persisted across all months, with HR consistently showing lower coupling probabilities (median ~10^{-6}) compared to MR and LR (median ~10^{-4} to 10^{-3}), confirming that the differences between methods represent fundamental characteristics of each aggregation approach rather than temporal fluctuations."

4) The discussion could also benefit from a broader reflection on how mobility patterns may differ between developing and developed countries. This would help contextualize the results and underline their relevance.

We thank the reviewer for this valuable suggestion. Mobility patterns do indeed differ between developing and developed contexts, with residents in low-income settings typically making fewer, shorter trips per day (mean ≈5 km), whereas those in high-income settings tend to make more frequent and longer ones (mean ≈10–15 km) [link]. That said, a substantial body of literature has established that human mobility is well described by parsimonious generative models, gravity and radiation models, across both high-income and low- and middle-income country (LMIC) settings (Barbosa al., Phys. Rep., 2018). Notably, Balcan et al. (PNAS, 2009) demonstrated that commuting flows can be reliably fitted at a global scale, suggesting that the fundamental structure of daily human circulation between home and regularly visited locations is a general feature of mobility that transcends development context.

The spatial scale of analysis may, however, affect the generalizability of our quantitative results. Using municipalities as spatial units allows us to capture substantial heterogeneity across locations; coarser aggregations would smooth over these differences and likely attenuate the observed magnitude of discrepancies between methods.

Importantly, the distinction between aggregation approaches is not primarily driven by network density: in our data, density remains relatively high across all methods (MR: 90%, LR: 84%, HR: 70%). Reducing the number of individual trajectories or trips would lower density uniformly across networks, but differences in long-range connectivity would persist. In high-resolution networks (HR), long-range connections are inherently underrepresented because only consecutive displacements are captured, whereas medium- and low-resolution approaches (MR, LR) retain these links through home–destination relationships. This structural difference, not overall density, explains why HR networks are less epidemiologically informative and represents a fundamental limitation of this method that is independent of setting: by chaining any two sequentially visited locations, it severs the epidemiologically meaningful connection between where people reside and where they spend significant time, thereby misrepresenting the coupling forces that drive epidemic invasion regardless of the characteristic trip distances or network properties of a given country.

Here is the paragraph that was added to the Discussion section:

"While this study focuses on Senegal, we are aware that mobility patterns differ across socioeconomic contexts, with implications for how aggregation methods may behave. In low-income settings, daily mobility often consists of fewer, shorter trips (mean ≈5 km), whereas in high-income settings, mobility typically exhibits more frequent and longer commuting trips (mean ≈10–15 km). That said, a substantial body of literature has established that human mobility is well described by parsimonious generative models, gravity and radiation models, across both high-income and low- and middle-income country (LMIC) settings[31]. Notably, Balcan et al.[6] *demonstrated that commuting flows can be reliably fitted at a global scale, suggesting that the fundamental structure of daily human circulation between home and regularly visited locations is a general feature of mobility that transcends development contexts. These structural differences, moreover, do not alter our methodological conclusions, because the failure of HR arises from trip fragmentation rather than from any property of trip length itself: by chaining sequentially visited locations, HR severs the epidemiologically meaningful connection between where people reside and where they spend significant time, misrepresenting the coupling forces that drive epidemic invasion regardless of characteristic trip distances. While the specific magnitude of differences between methods may vary with local mobility patterns, infrastructure, and socioeconomic conditions, the core distinction between origin-destination preservation and trip fragmentation should generalize across settings. The spatial scale of analysis may nonetheless affect the generalizability of our quantitative results, as using municipalities as spatial units allows us to capture substantial heterogeneity across locations, whereas coarser aggregations would smooth over these differences and likely attenuate the observed magnitude of discrepancies between methods. Indeed, we expect differences between methods to diminish at coarser scales, where averaging obscures the heterogeneities that distinguish approaches, but empirical evaluation across scales remains an important direction for future work. Validation in diverse geographic and economic contexts would further strengthen the universality of findings."*

Below I list several specific points that currently make the manuscript difficult to read. This list is not exhaustive. I strongly recommend a thorough review of the manuscript to improve clarity and precision.

• "The novelty in the model is the normalization factor of the force of infection that depends on the effective population in a given place, which is composed of all these three categories of people." Please specify the three categories, as only two are mentioned earlier.

This novelty aspect is now stated in the Methods as follows:

"The effective population represents a novel aspect in metapopulation modeling[4,5]. It includes both residents who remain in their home municipality and all individuals visiting from other locations during the day."

• Figure 1: please include a description of the subpanels in the caption.

We updated the caption as follows: "Figure 1. Mobility resolutions. (a) Individual trajectory showing mobile phone user movements throughout the day from home to work in the morning (top) and from

work to home in the afternoon (bottom), with color indicating number of activities (calls, messages) in each municipality. (b) Resulting epidemiological coupling networks for three aggregation methods: HR) High-resolution method[11] linking all consecutive call locations; MR) Medium-resolution method[12] linking home to all visited locations weighted by time spent; LR) Low-resolution method[13] linking home only to the most-visited location."

• "Connections between links depend on the aggregation process, so the resulting networks have different topologies". I believe "links" should be replaced with "nodes".

We replaced "links" with "nodes".

• "elements in D differ from the other two methods of an order of magnitude". A more precise quantification is needed, for example by comparing means or medians. From Figure 2a the difference appears to exceed one order of magnitude.

More exact details have been provided in the Results section as follow:
"Despite these high correlations, the median coupling probability in HR is approximately one order of magnitude lower than in MR and LR (1.5×10^{-4} in LR, 1.0×10^{-4} in MR, 8.4×10^{-6} in HR, Figure 2a). Largest differences are on links that connect Urban/Rural municipalities (Figure 2c)."

• "While C and L have a quite similar probability distribution of the coupling probabilities (Figure 3a)". The reference seems to be Figure 2a.

We replaced "Figure 3a" with "Figure 2a".

• "in D the median of the distribution is around 1 order of magnitude lower (Figure 2a)." From the figure this difference appears larger.

• "Figure 2b shows also differences between D, L and C on the outgoing probability in any location." The term "outgoing probability to any location" could be confused with the summed "outgoing probability". Using "coupling probabilities", as in Figure 2a, may improve clarity.

We replaced "outgoing probability to any location" with "coupling probabilities".

• "Such difference increases on municipalities far from the urban areas (Figure 2d,e,f)." The reference appears to be Figure 2b. Figures 2d, 2e and 2f refer to the summed outgoing probability. Clarifying this would help readers.

Figure 2 has been remade as requested by Review #1 and the urban/rural aspect is discussed in Figure 2c. In the text is reported like this:
"Conversely, outgoing probabilities are higher in HR compared with MR and LR (0.86 in HR, 0.62 in MR, 0.69 in LR; Figure 2b). Differences are mainly driven by rural areas in the southwest of Senegal (Figure 2d)."

• "In this latter case, the outgoing probability in D is around two orders of magnitude smaller compared with L and C." This statement should be supported with quantitative information. If it refers to Figure 2e, the difference appears smaller than one order of magnitude.

We added in the text:
"Outgoing probabilities is higher in HR compared with MR and LR (0.86 in HR, 0.62 in MR, 0.69 in LR(HR ≈ 0.86, LR ≈ 0.69, MR ≈MR≈ 0.62; Figure 2b)

• "We focused the comparison of the three matrices on i) common links and ii) links detected in one method and not in another one." Please indicate explicitly that this refers to Figure 3.

We updated the text as follows: "As a second analysis to quantify differences across metrices, we focused the comparison on i) common links and ii) links detected in one method and not in another one (Figure 3)."

• "Considering subsets of links that have relative variation higher than a certain cut-off,". Please specify the cut-off expression matching the label in the graph/.

We updated the text as follows: "When considering links with relative variation above a threshold ($\Delta^{HR,MR}(p_{ij})>x$), the most discrepant links show coupling probabilities in HR up to 1000 times lower than in MR (Figure 3a)."

• "Instead, C and L are quite similar." Although this is evident from the figures, a quantitative comparison would make the point more robust.

This part of the text was rewritten in particular, the comparison between MR and LR states is as follows:

"Coupling probabilities in LR are at most 10 times different from MR, and these differences remain stable across geographical distances (Figure 3b,d)."

• "Arrival time in D and C are in accord to observations on spatial transmission driven by commuting flows modelled by9 with a similar metapopulation approach, but with different source to infer coupling forces." This comparison should be elaborated, since it is used as a form of validation. It would also be useful to clarify whether mobility fluxes from reference 9 are directly comparable to those in Senegal.

Since a proper validation on COVID-19 early spread in Senegal has been performed, this part of the text has been removed.

• "Considering L and C, the median of the relative variation on the arrival times is 0 for each 0 (Figure 4a)". From Figure 4a the value does not appear to be zero, so this may require correction.

This sentence has been corrected. The median of the relative variation on the arrival times is around 40.

• Figure 4 lacks subfigure labels and red dots for the spreading seed. The caption refers to Kendall tau as a "probability", which should be amended.

Figure 4 labels for each subfigure have been corrected and clarified.

• "Little differences exist between L, L' and C, C', while relevant ones are found between D and D'. We found that in L' the outgoing probability decreases compared with L." These statements seem inconsistent. Please clarify whether the change in L' relative to L is meaningful.

The sentence was restructured as follows:

"While MR' and LR' showed minimal differences from their original counterparts in terms of epidemic outcomes, HR ' demonstrated substantial improvements over HR, producing spatial diffusion patterns more similar to MR and LR (Figures S15-19). However, HR' still exhibited limitations, with invasion distance distributions remaining higher than in MR and LR due to approximately 40% of epidemiologically relevant links present in MR and LR being absent in HR' (Figure S15)."

• "Adequately aggregating human movements become particularly relevant for improving reliability of projections of infection diseases models. On the other hand, it is also become". Please check verb forms.

This sentence has been corrected.

• "In fact, people leaving in a location are exposed to an infection". I believe "living" is intended.

We updated the word "leaving" with "living".

• Formula after "where is the coupling probability between patches i and j,". The subscript "i" appears inconsistent with other uses of the same symbol. The following formula also contains a double "+", which should be corrected.

We corrected the formula and updated all "i" in the text with "*i*".

• “We also evaluated the Kendall tau correlation coefficient for exploring if the ranking of the epidemic arrival time.” Please complete the sentence.

We updated the sentence as follows: “We also evaluated the Kendall tau correlation coefficient for exploring the ranking of the epidemic arrival times.”

Overall, the study addresses an important problem and provides valuable methodological insights. The scientific basis of the work is strong. Once the presentation is revised for clarity, consistency and precision, the manuscript will be considerably improved and more accessible to readers.
