# Supplementary material for: Mobility data resolution needed to inform predictive models of spatial epidemic spread from mobile phone data
Source: PLoS Comput Biol. 2026 Jul 2;22(7):e1014427. doi: 10.1371/journal.pcbi.1014427 (PMC13327251; doi:10.1371/journal.pcbi.1014427)
Supplement: Attachment — Submitted filename: Response_to_reviewers_auresp_2.pdf [file pcbi.1014427.s004.pdf]

POINT-BY-POINT REPLY TO EDITOR AND REVIEWERS' REMARKS

Additional Editor Comments:

Please address the reviewers' final comments and resubmit soon, thanks.

Note: If the reviewer comments include a recommendation to cite specific previously published works, please review and evaluate these publications to determine whether they are relevant and should be cited. There is no requirement to cite these works unless the editor has indicated otherwise.

Reviewer #1:

Thank you for addressing my comments. I have no further concerns.

Reviewer #2:

In the formula, \(I_i\) represents the total population of region \(i\). However, the effective number of infections \(\tilde{I}_i\) should inherently be time-dependent, as it includes not only the infected individuals currently present in region \(i\) (including both non-moving local infected individuals and infected visitors from other regions), but also those who were previously present in region \(i\) but have since returned to their place of residence. Nevertheless, in the current definition in the article, \(\tilde{I}_i = p_{ii}I_i + \sum_j p_{ji}I_j\) does not seem to explicitly account for this category of ``returned'' infected individuals. This may lead to an underestimation of the actual infection pressure in region \(i\), particularly in scenarios with high mobility or frequent short-distance commuting.

We thank the reviewer for this comment and for the opportunity to further clarify the interpretation of the model framework and the effective infectious population $\hat{I}_i$. In our model, I_i denotes the number of infectious individuals whose home region is i, while the coupling matrix element p_{ij} represents probability of residents of region i present in region j during a given model time step (one day). Accordingly, the quantity

$$\hat{I}_i = p_{ii} I_i + \sum_j p_{ji} I_j$$

represents the effective number of infectious individuals physically present in region i at day t, including both non-traveling local residents and infectious visitors from other regions in that day.

The reviewer raises the case of infectious individuals who were previously present in region i and then returned to their home region. In our modeling framework, these individuals are not omitted; rather, their contribution is explicitly incorporated through the third term of the force of infection, λ_{ij}^{r}, which accounts for residents of i who visit region j, get infected there, and return to i within the same time step. This pathway, together with local transmission (λ_{ii}) and infection by incoming visitors (λ_{ji}^{v}), captures the transmission dynamics across all co-location configurations at each time step.

This three-term structure follows the equilibrium solution of the mobility dynamics, as formally derived in Balcan et al. (2009, PNAS, SI Sections 2.3–2.4), that is valid when the mobility cycle is shorter to the disease timescale. In our setting, individuals return to their home municipality within the same day (τ^{-1} = 1 day), while the latency period is $\varepsilon^{-1} \approx 1.5$ days and the infectious period is $\mu^{-1} \approx 3$ days with a total generation time of 4.5 days, satisfying this condition. Our model is therefore re-applying the equilibrium solution at each timestep; it does not track cumulative past presence, but assumes that the force of infection is determined by the instantaneous mobility configuration at each time step. This same framework has been used in recent comparable studies (Sabbatini et al., BMC Infectious Diseases, 2024; Pullano et al., JMIR Public Health and Surveillance, 2025).

To avoid possible confusion, we have revised the section "Epidemic metapopulation model" in the manuscript as follows:

"**Epidemic metapopulation model**

To quantify the impact of the coupling matrices on the modelled epidemic diffusion, we developed a stochastic, discrete-time and non-Markovian metapopulation model[4,5]. The model accounts for disease transmission arising from spatial co-location of individuals due to daily mobility patterns. The Senegalese population was spatially divided in 394 municipalities, with links between municipalities defined by the time-varying coupling matrices p_{ij}.

The force of infection in location i at time t is given by:

$$\lambda_i = \lambda_{ii} + \sum_{i \neq j} \lambda_{ji}^{v} + \sum_{i \neq j} \lambda_{ij}^{r}$$

with

$$\lambda_{ii} = \beta p_{ii}^{2} \frac{I_i}{\widehat{N_\imath}};\ \lambda_{ji}^{v} = \beta\, p_{ii} p_{ji} \frac{I_j}{\widehat{N_\imath}};\ \lambda_{ij}^{r} = \beta\, p_{ij} \frac{\widehat{I_j}}{\widehat{N_j}}$$

where p_{ij} is the coupling probability between location i and j at time t and β the transmissibility rate. The time index t is omitted from the formulas for clarity.

The three terms of the force of infections correspond to three distinct transmission contributions: (λ_{ii}) residents who remain in their home municipality and get infected locally; (λ_{ji}^{v}) infected visitors from other municipalities temporarily present in i; and (λ_{ij}^{r}) residents of i who are visiting location j for the day, get infected in j and return to i. All three pathways are implicitly accounted for through the coupling probabilities p_{ij}, consistently with the first-order equilibrium solution derived analytically in Balcan et al[14].

The effective population at time t is:

$$\widehat{N_\imath} = p_{ii} N_i + \sum_{j} p_{ji} N_j$$

and the effective number of infections in i at time t is:

$$\widehat{I_\imath} = p_{ii} I_i + \sum_{j} p_{ji} I_j$$

$\widehat{N_\imath}$ *and* $\widehat{I_\imath}$ are time-dependent quantities recomputed at each discrete time step to reflect the instantaneous mixing configuration of the population across locations."

Reviewer #3:

I thank the authors for addressing all my comments. I believe that the manuscript clarity has been improved and its scientific value further strengthened. I therefore recommend the paper for publication.
