# Supplementary material for: Mobility data resolution needed to inform predictive models of spatial epidemic spread from mobile phone data
Source: PLoS Comput Biol. 2026 Jul 2;22(7):e1014427. doi: 10.1371/journal.pcbi.1014427 (PMC13327251; doi:10.1371/journal.pcbi.1014427)
Supplement: Peer review history

# Peer review: Decision Letter 0

**DOI:** 10.1371/journal.pcbi.1014427.r001

**Authors:** Shi Zhao, Denise Kühnert

1 Dec 2025

PCOMPBIOL-D-25-02287

Mobility resolution needed to inform predictive epidemic models for spatial transmission from mobile phone data

PLOS Computational Biology

Dear Dr. Colizza,

Thank you for submitting your manuscript to PLOS Computational Biology. After careful consideration, we feel that it has merit but does not fully meet PLOS Computational Biology's publication criteria as it currently stands. Therefore, we invite you to submit a revised version of the manuscript that addresses the points raised during the review process.

Please submit your revised manuscript by Jan 31 2026 11:59PM. If you will need more time than this to complete your revisions, please reply to this message or contact the journal office at ploscompbiol@plos.org. When you're ready to submit your revision, log on to https://www.editorialmanager.com/pcompbiol/ and select the 'Submissions Needing Revision' folder to locate your manuscript file.

Please include the following items when submitting your revised manuscript:

* A rebuttal letter that responds to each point raised by the editor and reviewer(s). You should upload this letter as a separate file labeled 'Response to Reviewers'. This file does not need to include responses to formatting updates and technical items listed in the 'Journal Requirements' section below.

* A marked-up copy of your manuscript that highlights changes made to the original version. You should upload this as a separate file labeled 'Revised Manuscript with Track Changes'.

* An unmarked version of your revised paper without tracked changes. You should upload this as a separate file labeled 'Manuscript'.

If you would like to make changes to your financial disclosure, competing interests statement, or data availability statement, please make these updates within the submission form at the time of resubmission. Guidelines for resubmitting your figure files are available below the reviewer comments at the end of this letter

We look forward to receiving your revised manuscript.

Kind regards,

Shi Zhao

Academic Editor

PLOS Computational Biology

Denise Kühnert

Section Editor

PLOS Computational Biology

**Additional Editor Comments:**

Please make all code and data available. If some data cannot be shared due to privacy issues, please explain.

**Journal Requirements:**

1) Please ensure that the CRediT author contributions listed for every co-author are completed accurately and in full.

At this stage, the following Authors/Authors require contributions: Giulia Pullano, Shweta Bansal, Stefania Rubrichi, and Vittoria Colizza. Please ensure that the full contributions of each author are acknowledged in the "Add/Edit/Remove Authors" section of our submission form.

The list of CRediT author contributions may be found here: https://journals.plos.org/ploscompbiol/s/authorship#loc-author-contributions

2) We ask that a manuscript source file is provided at Revision. Please upload your manuscript file as a .doc, .docx, .rtf or .tex. If you are providing a .tex file, please upload it under the item type u2018LaTeX Source Fileu2019 and leave your .pdf version as the item type u2018Manuscriptu2019.

3) Please provide an Author Summary. This should appear in your manuscript between the Abstract (if applicable) and the Introduction, and should be 150-200 words long. The aim should be to make your findings accessible to a wide audience that includes both scientists and non-scientists. Sample summaries can be found on our website under Submission Guidelines:

https://journals.plos.org/ploscompbiol/s/submission-guidelines#loc-parts-of-a-submission

4) Please upload all main figures as separate Figure files in .tif or .eps format. For more information about how to convert and format your figure files please see our guidelines:

https://journals.plos.org/ploscompbiol/s/figures

5) We have noticed that you have uploaded Supporting Information files, but you have not included a list of legends. Please add a full list of legends for your Supporting Information files after the references list.

6) We note that your Data Availability Statement is currently as follows: "no". Please confirm at this time whether or not your submission contains all raw data required to replicate the results of your study. Authors must share the “minimal data set” for their submission. PLOS defines the minimal data set to consist of the data required to replicate all study findings reported in the article, as well as related metadata and methods (https://journals.plos.org/plosone/s/data-availability#loc-minimal-data-set-definition).

For example, authors should submit the following data:

- The values behind the means, standard deviations and other measures reported;

- The values used to build graphs;

- The points extracted from images for analysis..

Authors do not need to submit their entire data set if only a portion of the data was used in the reported study.

If your submission does not contain these data, please either upload them as Supporting Information files or deposit them to a stable, public repository and provide us with the relevant URLs, DOIs, or accession numbers. For a list of recommended repositories, please see https://journals.plos.org/plosone/s/recommended-repositories.

If there are ethical or legal restrictions on sharing a de-identified data set, please explain them in detail (e.g., data contain potentially sensitive information, data are owned by a third-party organization, etc.) and who has imposed them (e.g., an ethics committee). Please also provide contact information for a data access committee, ethics committee, or other institutional body to which data requests may be sent. If data are owned by a third party, please indicate how others may request data access.

7) Please amend your detailed Financial Disclosure statement. This is published with the article. It must therefore be completed in full sentences and contain the exact wording you wish to be published.

- State the initials, alongside each funding source, of each author to receive each grant. For example: "This work was supported by the National Institutes of Health (####### to AM; ###### to CJ) and the National Science Foundation (###### to AM)."

- State what role the funders took in the study. If the funders had no role in your study, please state: "The funders had no role in study design, data collection and analysis, decision to publish, or preparation of the manuscript.".

If you did not receive any funding for this study, please simply state: u201cThe authors received no specific funding for this work.u201d

**Reviewers' comments:**

Reviewer's Responses to Questions

**Comments to the Authors:**

**Please note here if the review is uploaded as an attachment.**

Reviewer #1: This manuscript explores how the definition and aggregation of mobile phone–derived mobility data influence epidemic modeling outcomes. While the analysis is technically sound, the overall research question is somewhat limited in scope. The key issue in using mobile phone data for epidemiological modeling lies more in the privacy, ethical, and governance challenges surrounding data access and use. The manuscript would be stronger if it acknowledged and discussed these broader implications more explicitly.

1. The final paragraph of the Introduction reads more like a methodological description than a study rationale. It introduces too many technical details that would be better placed in the Methods section. The Introduction would be clearer if it briefly summarized the motivation and study objective instead of detailing the data aggregation procedures.

2. The Methods section is dense and overlaps with Results. Please consider restructuring into clear subsections, moving formulas and parameter details to Supplementary Information.

3. The study lack of empirical validation. While the authors acknowledge this, at least some retrospective comparison with known epidemic or cross-validation using synthetic mobility data would strengthen credibility.

4. The MRQAP correlation analysis shows matrices are “highly correlated,” yet the epidemiological implications of these differences are not fully quantified. Provide additional quantitative metrics to illustrate the magnitude of divergence.

5. Figures 2–6 are information-dense and sometimes repetitive. The Results would benefit from a more streamlined structure.

6. The conclusion that “tracking individual displacements is less epidemiologically informative than focusing on most visited locations” is compelling but should be contextualized. Are these findings generalizable beyond Senegal, or dependent on network density and mobility scale? The statement that “privacy can be enhanced without losing epidemiological value” is conceptually strong but speculative; it should be qualified more carefully.

7. Please clarify whether ₀ = 1.1–3 corresponds to realistic ranges for the diseases simulated.

Reviewer #2: Based on mobile phone data encompassing 9,569,425 users in Senegal from January to December 2013, the authors quantified corresponding coupling matrices to evaluate the influence of mobility definitions on disease spread. This study presents interesting investigation. Intuitively, one might assume that higher spatial resolution would enhance the predictive power of epidemic models. However, the results of this study suggest the opposite, which is somewhat counterintuitive. In fact, the required resolution depends heavily on the specific scientific question being addressed and the perspective from which it is examined. Additionally, the following issues warrant further clarification or elaboration by the authors:

1. Definition of Coupling Force: Could the authors provide a detailed explanation of what "coupling" refers to in this context? Why are the number of calls or text messages used as proxies for coupling? The direct relationship between call/SMS frequency and disease transmission remains unclear.

2. Magnitude Discrepancy in Matrix D: What accounts for the order-of-magnitude difference between the elements of matrix D and those of the other two matrices?

3. Unexpected Performance of High-Resolution Model D: Why does matrix D, despite its higher resolution, yield the least realistic epidemic simulations? Could this be related to the structure of the transmission model or the specific transmission route of the pathogen (e.g., aerosol transmission)? Since D captures path-based mobility, would an edge-based network modeling approach be more suitable for it? In contrast, L and C appear more node-centered, which aligns well with node-based modeling frameworks. For many respiratory infectious diseases with complex transmission pathways, how significant is the role of such coupling forces?

4. Temporal Resolution of Coupling Matrices: The authors used monthly coupling matrices. Why was a daily resolution not adopted, especially since the SEIR model operates on a daily time step? Would a daily matrix provide better temporal alignment with the disease dynamics?

Reviewer #3: The paper addresses an important and timely question, namely how to incorporate mobility data into epidemic models when mobile phone data are available. The authors explore three implementations with varying levels of granularity and privacy, embed them in a metapopulation infectious disease model, and assess the resulting differences. The topic is highly relevant for the epidemic modeling community, and the methodological foundations are generally sound. However, in its current form, the manuscript is challenging to follow. I encourage the authors to substantially revise the presentation of the results. Below I outline several major points that could help strengthen the paper.

• Even if full calibration with real data is beyond the scope of the study, the central claim that L and C are sufficient to reproduce realistic patterns of diffusion would benefit from a brief comparison between simulated epidemic patterns and real observations for Senegal. This would ground the main conclusions and increase the impact of the work.

• A more consolidated summary in the discussion section of the measures used to compare the three mobility matrices, as well as of the key findings, would improve readability. Some of these elements are already present in the results section, and moving them into a single coherent discussion would help readers approach that section independently.

• The treatment of temporal variation in mobility is not fully clear. The authors state that they extract twelve monthly mobility matrices for each implementation. A more detailed discussion of the temporal patterns of these matrices would be valuable and might provide additional epidemiological insights. In addition, it is not explained how the monthly matrices are used in the comparisons. For example, are L and C compared month by month, or are values aggregated? For Figure 2a the methodology is explicit, but the same level of clarity is needed throughout. The current description of the hierarchical clustering does not fully resolve this point.

• The discussion could also benefit from a broader reflection on how mobility patterns may differ between developing and developed countries. This would help contextualize the results and underline their relevance.

Below I list several specific points that currently make the manuscript difficult to read. This list is not exhaustive. I strongly recommend a thorough review of the manuscript to improve clarity and precision.

• “The novelty in the model is the normalization factor of the force of infection that depends on the effective population in a given place, which is composed of all these three categories of people.” Please specify the three categories, as only two are mentioned earlier.

• Figure 1: please include a description of the subpanels in the caption.

• “Connections between links depend on the aggregation process, so the resulting networks have different topologies”. I believe “links” should be replaced with “nodes”.

• “elements in D differ from the other two methods of an order of magnitude”. A more precise quantification is needed, for example by comparing means or medians. From Figure 2a the difference appears to exceed one order of magnitude.

• “While C and L have a quite similar probability distribution of the coupling probabilities (Figure 3a)”. The reference seems to be Figure 2a.

• “in D the median of the distribution is around 1 order of magnitude lower (Figure 2a).” From the figure this difference appears larger.

• “Figure 2b shows also differences between D, L and C on the outgoing probability in any location.” The term “outgoing probability to any location” could be confused with the summed “outgoing probability”. Using “coupling probabilities”, as in Figure 2a, may improve clarity.

• “Such difference increases on municipalities far from the urban areas (Figure 2d,e,f).” The reference appears to be Figure 2b. Figures 2d, 2e and 2f refer to the summed outgoing probability. Clarifying this would help readers.

• “In this latter case, the outgoing probability in D is around two orders of magnitude smaller compared with L and C.” This statement should be supported with quantitative information. If it refers to Figure 2e, the difference appears smaller than one order of magnitude.

• “We focused the comparison of the three matrices on i) common links and ii) links detected in one method and not in another one.” Please indicate explicitly that this refers to Figure 3.

• “Considering subsets of links that have relative variation higher than a certain cut-off,”. Please specify the cut-off expression matching the label in the graph/.

• “Instead, C and L are quite similar.” Although this is evident from the figures, a quantitative comparison would make the point more robust.

• “Arrival time in D and C are in accord to observations on spatial transmission driven by commuting flows modelled by9 with a similar metapopulation approach, but with different source to infer coupling forces.” This comparison should be elaborated, since it is used as a form of validation. It would also be useful to clarify whether mobility fluxes from reference 9 are directly comparable to those in Senegal.

• “Considering L and C, the median of the relative variation on the arrival times is 0 for each 0 (Figure 4a)”. From Figure 4a the value does not appear to be zero, so this may require correction.

• Figure 4 lacks subfigure labels and red dots for the spreading seed. The caption refers to Kendall tau as a “probability”, which should be amended.

• “Little differences exist between L, L' and C, C', while relevant ones are found between D and D'. We found that in L' the outgoing probability decreases compared with L.” These statements seem inconsistent. Please clarify whether the change in L' relative to L is meaningful.

• “Adequately aggregating human movements become particularly relevant for improving reliability of projections of infection diseases models. On the other hand, it is also become”. Please check verb forms.

• “In fact, people leaving in a location are exposed to an infection”. I believe “living” is intended.

• Formula after “where is the coupling probability between patches i and j,”. The subscript “i” appears inconsistent with other uses of the same symbol. The following formula also contains a double “+”, which should be corrected.

• “We also evaluated the Kendall tau correlation coefficient for exploring if the ranking of the epidemic arrival time.” Please complete the sentence.

Overall, the study addresses an important problem and provides valuable methodological insights. The scientific basis of the work is strong. Once the presentation is revised for clarity, consistency and precision, the manuscript will be considerably improved and more accessible to readers.

[NOTE: If reviewer comments were submitted as an attachment file, they will be attached to this email and accessible via the submission site. Please log into your account, locate the manuscript record, and check for the action link "View Attachments". If this link does not appear, there are no attachment files.]

**Figure resubmission:**

While revising your submission, we strongly recommend that you use PLOS’s NAAS tool (https://ngplosjournals.pagemajik.ai/artanalysis) to test your figure files. NAAS can convert your figure files to the TIFF file type and meet basic requirements (such as print size, resolution), or provide you with a report on issues that do not meet our requirements and that NAAS cannot fix.

After uploading your figures to PLOS’s NAAS tool - https://ngplosjournals.pagemajik.ai/artanalysis, NAAS will process the files provided and display the results in the "Uploaded Files" section of the page as the processing is complete. If the uploaded figures meet our requirements (or NAAS is able to fix the files to meet our requirements), the figure will be marked as "fixed" above. If NAAS is unable to fix the files, a red "failed" label will appear above. When NAAS has confirmed that the figure files meet our requirements, please download the file via the download option, and include these NAAS processed figure files when submitting your revised manuscript.

**Reproducibility:**

To enhance the reproducibility of your results, we recommend that authors of applicable studies deposit laboratory protocols in protocols.io, where a protocol can be assigned its own identifier (DOI) such that it can be cited independently in the future. Additionally, PLOS ONE offers an option to publish peer-reviewed clinical study protocols. Read more information on sharing protocols at https://plos.org/protocols?utm_medium=editorial-email&utm_source=authorletters&utm_campaign=protocols

# Author comment: Author response to Decision Letter 1

**DOI:** 10.1371/journal.pcbi.1014427.r002

12 Mar 2026

Attachment Submitted filename: Response_to_reviewers.pdf

# Peer review: Decision Letter 1

**DOI:** 10.1371/journal.pcbi.1014427.r003

**Authors:** Shi Zhao, Denise Kühnert, Shi Zhao, Denise Kühnert

29 Apr 2026

PCOMPBIOL-D-25-02287R1

Mobility data resolution needed to inform predictive models of spatial epidemic spread from mobile phone data

PLOS Computational Biology

Dear Dr. Colizza,

Thank you for submitting your manuscript to PLOS Computational Biology. After careful consideration, we feel that it has merit but does not fully meet PLOS Computational Biology's publication criteria as it currently stands. Therefore, we invite you to submit a revised version of the manuscript that addresses the points raised during the review process.

Please submit your revised manuscript by Jun 29 2026 11:59PM. If you will need more time than this to complete your revisions, please reply to this message or contact the journal office at ploscompbiol@plos.org. When you're ready to submit your revision, log on to https://www.editorialmanager.com/pcompbiol/ and select the 'Submissions Needing Revision' folder to locate your manuscript file.

Please include the following items when submitting your revised manuscript:

* A letter that responds to each point raised by the editor and reviewer(s). You should upload this letter as a separate file labeled 'Response to Reviewers'. This file does not need to include responses to formatting updates and technical items listed in the 'Journal Requirements' section below.

* A marked-up copy of your manuscript that highlights changes made to the original version. You should upload this as a separate file labeled 'Revised Manuscript with Track Changes'.

* An unmarked version of your revised paper without tracked changes. You should upload this as a separate file labeled 'Manuscript'.

If you would like to make changes to your financial disclosure, competing interests statement, or data availability statement, please make these updates within the submission form at the time of resubmission. Guidelines for resubmitting your figure files are available below the reviewer comments at the end of this letter.

We look forward to receiving your revised manuscript.

Kind regards,

Shi Zhao

Academic Editor

PLOS Computational Biology

Denise Kühnert

Section Editor

PLOS Computational Biology

**Additional Editor Comments :**

Please address the reviewers' final comments and resubmit soon, thanks.

**********

Note: If the reviewer comments include a recommendation to cite specific previously published works, please review and evaluate these publications to determine whether they are relevant and should be cited. There is no requirement to cite these works unless the editor has indicated otherwise.

**Reviewers' comments:**

Reviewer's Responses to Questions

Reviewer #1: Thank you for addressing my comments. I have no further concerns.

Reviewer #2: In the formula, \(I_i\) represents the total population of region \(i\). However, the effective number of infections \(\tilde{I}_i\) should inherently be time-dependent, as it includes not only the infected individuals currently present in region \(i\) (including both non-moving local infected individuals and infected visitors from other regions), but also those who were previously present in region \(i\) but have since returned to their place of residence. Nevertheless, in the current definition in the article, \(\tilde{I}_i = p_{ii}I_i + \sum_j p_{ji}I_j\) does not seem to explicitly account for this category of ``returned'' infected individuals. This may lead to an underestimation of the actual infection pressure in region \(i\), particularly in scenarios with high mobility or frequent short-distance commuting.

Reviewer #3: I thank the authors for addressing all my comments. I believe that the manuscript clarity has been improved and its scientific value further strengthened. I therefore recommend the paper for publication.

**********

**Have the authors made all data and (if applicable) computational code underlying the findings in their manuscript fully available?**

The PLOS Data policy requires authors to make all data and code underlying the findings described in their manuscript fully available without restriction, with rare exception (please refer to the Data Availability Statement in the manuscript PDF file). The data and code should be provided as part of the manuscript or its supporting information, or deposited to a public repository. For example, in addition to summary statistics, the data points behind means, medians and variance measures should be available. If there are restrictions on publicly sharing data or code —e.g. participant privacy or use of data from a third party—those must be specified.

Reviewer #1: None

Reviewer #2: Yes

Reviewer #3: Yes

**********

PLOS authors have the option to publish the peer review history of their article (what does this mean?). If published, this will include your full peer review and any attached files.

If you choose “no”, your identity will remain anonymous but your review may still be made public.

**Do you want your identity to be public for this peer review?**  For information about this choice, including consent withdrawal, please see our Privacy Policy.

Reviewer #1: No

Reviewer #2: No

Reviewer #3: No

[NOTE: If reviewer comments were submitted as an attachment file, they will be attached to this email and accessible via the submission site. Please log into your account, locate the manuscript record, and check for the action link "View Attachments". If this link does not appear, there are no attachment files.]

**Figure resubmission:**

-->While revising your submission, we strongly recommend that you use PLOS’s NAAS tool (https://ngplosjournals.pagemajik.ai/artanalysis) to test your figure files. NAAS can convert your figure files to the TIFF file type and meet basic requirements (such as print size, resolution), or provide you with a report on issues that do not meet our requirements and that NAAS cannot fix.

After uploading your figures to PLOS’s NAAS tool - https://ngplosjournals.pagemajik.ai/artanalysis, NAAS will process the files provided and display the results in the "Uploaded Files" section of the page as the processing is complete. If the uploaded figures meet our requirements (or NAAS is able to fix the files to meet our requirements), the figure will be marked as "fixed" above. If NAAS is unable to fix the files, a red "failed" label will appear above. When NAAS has confirmed that the figure files meet our requirements, please download the file via the download option, and include these NAAS processed figure files when submitting your revised manuscript.

**Reproducibility:**

To enhance the reproducibility of your results, we recommend that authors of applicable studies deposit laboratory protocols in protocols.io, where a protocol can be assigned its own identifier (DOI) such that it can be cited independently in the future. Additionally, PLOS ONE offers an option to publish peer-reviewed clinical study protocols. Read more information on sharing protocols at https://plos.org/protocols?utm_medium=editorial-email&utm_source=authorletters&utm_campaign=protocols

# Author comment: Author response to Decision Letter 2

**DOI:** 10.1371/journal.pcbi.1014427.r004

11 May 2026

Attachment Submitted filename: Response_to_reviewers_auresp_2.pdf

# Editor report: Decision Letter 2

**DOI:** 10.1371/journal.pcbi.1014427.r005

**Authors:** Shi Zhao, Denise Kühnert, Shi Zhao, Denise Kühnert, Shi Zhao, Denise Kühnert

9 Jun 2026

Dear Dr. Colizza,

We are pleased to inform you that your manuscript 'Mobility data resolution needed to inform predictive models of spatial epidemic spread from mobile phone data' has been provisionally accepted for publication in PLOS Computational Biology.

Before your manuscript can be formally accepted you will need to complete some formatting changes, which you will receive in a follow up email. A member of our team will be in touch with a set of requests.

Please note that your manuscript will not be scheduled for publication until you have made the required changes, so a swift response is appreciated.

IMPORTANT: The editorial review process is now complete. PLOS will only permit corrections to spelling, formatting or significant scientific errors from this point onwards. Requests for major changes, or any which affect the scientific understanding of your work, will cause delays to the publication date of your manuscript.

Should you, your institution's press office or the journal office choose to press release your paper, you will automatically be opted out of early publication. We ask that you notify us now if you or your institution is planning to press release the article. All press must be co-ordinated with PLOS.

Thank you again for supporting Open Access publishing; we are looking forward to publishing your work in PLOS Computational Biology.

Best regards,

Shi Zhao

Academic Editor

PLOS Computational Biology

Denise Kühnert

Section Editor

PLOS Computational Biology

***********************************************************

# Editor report: Acceptance letter

**DOI:** 10.1371/journal.pcbi.1014427.r006

**Authors:** Shi Zhao, Denise Kühnert, Shi Zhao, Denise Kühnert, Shi Zhao, Denise Kühnert

PCOMPBIOL-D-25-02287R2

Mobility data resolution needed to inform predictive models of spatial epidemic spread from mobile phone data

Dear Dr Colizza,

I am pleased to inform you that your manuscript has been formally accepted for publication in PLOS Computational Biology. Your manuscript is now with our production department and you will be notified of the publication date in due course.

The corresponding author will soon be receiving a typeset proof for review, to ensure errors have not been introduced during production. Please review the PDF proof of your manuscript carefully, as this is the last chance to correct any errors. Please note that major changes, or those which affect the scientific understanding of the work, will likely cause delays to the publication date of your manuscript.

Soon after your final files are uploaded, unless you have opted out, the early version of your manuscript will be published online. The date of the early version will be your article's publication date. The final article will be published to the same URL, and all versions of the paper will be accessible to readers.

For Research, Software, and Methods articles, you will receive an invoice from PLOS for your publication fee after your manuscript has reached the completed accept phase. If you receive an email requesting payment before acceptance or for any other service, this may be a phishing scheme. Learn how to identify phishing emails and protect your accounts at https://explore.plos.org/phishing.

Thank you again for supporting PLOS Computational Biology and open-access publishing. We are looking forward to publishing your work!

With kind regards,

Zsofia Freund

PLOS Computational Biology | Carlyle House, Carlyle Road, Cambridge CB4 3DN | United Kingdom ploscompbiol@plos.org | Phone +44 (0) 1223-442824 | ploscompbiol.org | @PLOSCompBiol
